# Genetic Diversity and Selective Pressure in Hepatitis C Virus Genotypes 1–6: Significance for Direct-Acting Antiviral Treatment and Drug Resistance

**DOI:** 10.3390/v7092857

**Published:** 2015-09-16

**Authors:** Lize Cuypers, Guangdi Li, Pieter Libin, Supinya Piampongsant, Anne-Mieke Vandamme, Kristof Theys

**Affiliations:** 1 KU Leuven—University of Leuven, Department of Microbiology and Immunology, Rega Institute for Medical Research, Clinical and Epidemiological Virology, Minderbroedersstraat 10, Leuven 3000, Belgium; liguangdi.research@gmail.com (G.L.); pieter.libin@vub.ac.be (P.L.); annemie.vandamme@uzleuven.be (A.-M.V.); kristof.theys@rega.kuleuven.be (K.T.); 2 Metabolic Syndrome Research Center, the Second Xiangya Hospital, Central South University, Changsha 410011, China; 3 Artificial Intelligence Lab, Vrije Universiteit Brussel, Pleinlaan 2, Brussels 1050, Belgium; 4 Department of Electrical Engineering ESAT, STADIUS Center for Dynamical Systems, Signal Processing and Data Analytics, KU Leuven, University of Leuven, Kasteelpark Arenberg 10, Heverlee 3001, Belgium; supinya.piampongsant@esat.kuleuven.be; 5 Center for Global Health and Tropical Medicine, Microbiology Unit, Institute for Hygiene and Tropical Medicine, University Nova of Lisboa, Rua da Junqueira 100, Lisbon 1349-008, Portugal

**Keywords:** diversity, genotypes, HCV, direct-acting antiviral treatment, primers, drug resistance

## Abstract

Treatment with pan-genotypic direct-acting antivirals, targeting different viral proteins, is the best option for clearing hepatitis C virus (HCV) infection in chronically infected patients. However, the diversity of the HCV genome is a major obstacle for the development of antiviral drugs, vaccines, and genotyping assays. In this large-scale analysis, genome-wide diversity and selective pressure was mapped, focusing on positions important for treatment, drug resistance, and resistance testing. A dataset of 1415 full-genome sequences, including genotypes 1–6 from the Los Alamos database, was analyzed. In 44% of all full-genome positions, the consensus amino acid was different for at least one genotype. Focusing on positions sharing the same consensus amino acid in all genotypes revealed that only 15% was defined as pan-genotypic highly conserved (≥99% amino acid identity) and an additional 24% as pan-genotypic conserved (≥95%). Despite its large genetic diversity, across all genotypes, codon positions were rarely identified to be positively selected (0.23%–0.46%) and predominantly found to be under negative selective pressure, suggesting mainly neutral evolution. For NS3, NS5A, and NS5B, respectively, 40% (6/15), 33% (3/9), and 14% (2/14) of the resistance-related positions harbored as consensus the amino acid variant related to resistance, potentially impeding treatment. For example, the NS3 variant 80K, conferring resistance to simeprevir used for treatment of HCV1 infected patients, was present in 39.3% of the HCV1a strains and 0.25% of HCV1b strains. Both NS5A variants 28M and 30S, known to be associated with resistance to the pan-genotypic drug daclatasvir, were found in a significant proportion of HCV4 strains (10.7%). NS5B variant 556G, known to confer resistance to non-nucleoside inhibitor dasabuvir, was observed in 8.4% of the HCV1b strains. Given the large HCV genetic diversity, sequencing efforts for resistance testing purposes may need to be genotype-specific or geographically tailored.

## 1. Introduction

Despite 20 years of intensive research, a vaccine to prevent infection with the hepatitis C virus (HCV) remains elusive, while two million new HCV infections are estimated to occur worldwide every year [1]. Currently, 170 million people are chronically infected with HCV, at risk for cirrhosis (20%), end-stage liver disease (6%), and hepatocellular carcinoma (HCC) (4%) [2]. A preventive vaccine would need to induce broad reactive immunity in order to cope with the extensive genomic diversity of HCV [3]. HCV diversity is classified into seven genetically distinct genotypes (HCV 1–7) [4] that differ by more than 30% at nucleotide (NT) level, and into more than 50 subtypes that differ between 15% and 25% at nucleotide level within genotypes [5,6]. Substantial differences exist in the geographic distribution of HCV genotypes, with genotypes 1, 2, and 3 circulating worldwide, although with different predominance according to geographical areas [7]. HCV genotype 4 appears to be mainly found in Africa and the Middle East, and genotypes 5 and 6 are confined to Southern Africa and South East Asia, respectively. HCV belongs to the Flaviviridae family, and has a single-stranded RNA genome encoding a large polyprotein of 3011 amino acids (AA), which is processed into four structural and six non-structural (NS) proteins. A major barrier for the development of vaccines, broadly active antivirals, and assays, is the high genetic diversity of HCV and its potential to quickly adapt to different environments [8].

HCV is under constant immunological pressure. Neutralizing antibody response of the host is targeting mainly the viral envelope proteins E1 and E2. The virus manages to escape due to the large plasticity in the highly variable regions in these proteins [9], and effective targeting of conserved regions in the genome may improve vaccine design [10]. An alternative approach is a T-cell based vaccine that induces potent T-cell immune responses by antigen delivery with replication-defective recombinant viral vectors [11].

While vaccine design is still under experimental stage, development of direct-acting antivirals (DAAs) has progressed into clinical practice. The advent of DAAs dramatically improved treatment success rates compared to the previous standard-of-care (SOC) treatment with pegylated interferon-α (pegIFN-α) and ribavirin [12]. The first generation of NS3/4A protease inhibitors, telaprevir and boceprevir, achieved sustained virological response rates (SVR) above 70% [13] but emergence of drug resistance, severe adverse effects, and limited pan-genotypic activity remain barriers to efficacious treatment [14]. Both telaprevir and boceprevir have only been approved for HCV genotype 1 infected patients, and have now become contraindicated [15]. Recent generations of DAAs include the NS3/4A inhibitors simeprevir and paritaprevir, the NS5B polymerase inhibitor sofosbuvir, the NS5A inhibitors ledipasvir and daclatasvir, and the first interferon-free combination of these three drug classes,“Viekira Pak”. According to the most recent guidelines [15], genotype-specific treatment regimens need to be considered since the antiviral activity of DAAs differs according to HCV genotype. NS3/4A protease inhibitors simeprevir and paritaprevir are approved for treatment of HCV genotype 1 and 4 infected patients, while NS5B polymerase inhibitor sofosbuvir can be used in all genotypes. NS5A inhibitor daclatasvir was approved for all HCV genotypes as well, in contrast to ledipasvir, which showed no antiviral activity in genotypes 2 and 3 (Table S1). While success rates above 90% are achieved due to higher genetic barriers to resistance and broader antiviral activity [16,17,18], these newer drugs are also characterized by high costs that render the prospect of treating millions of infected people worldwide daunting, even for wealthier countries. Moreover, the first occurrence of transmission of a telaprevir resistant HCV strain [19] and reports of high incidence of re-infection following spontaneous clearance or cure (sustained virological response or SVR) in specific risk groups [20,21,22] may create a situation in which new pan-genotypic DAAs will be needed. Additionally, since medication adherence is expected to be lower in real-world settings, emergence or spread of drug resistant variants may potentially lead to treatment failure and affect treatment options. So far, the impact of genetic diversity on the presence of drug resistance, either at baseline or under treatment, is not well described.

HCV drug resistance testing prior to treatment initiation or at the time of treatment failure is currently not recommended for most HCV patients. Nevertheless, testing the presence of natural polymorphism 80K is required for treating HCV1a before using protease inhibitor simeprevir, whereas drug resistance testing in HIV infected patients is recommended both prior to treatment and at treatment failure [23]. Resistant variants were selected rapidly during DAA monotherapy with first generation protease inhibitors [24], with the variant pattern depending on the drug and the viral subtype. Naturally occurring resistance variants with decreased sensitivity to protease inhibitors have only been reported at low prevalence [25], although 80K has been reported at high prevalence in HCV1a (20%–34%) [26,27]. For NS5A inhibitors, the prevalence of naturally occurring resistance variants mostly ranges from 10% to 14%, and their presence is largely associated with lower SVR rates [28]. Despite the high genetic barrier of NS5B polymerase inhibitors, substitutions with low frequencies at various amino acid positions were associated with treatment failure in a subset of patients [29].

Genetic sequencing protocols with high reproducibility and sensitivity for all circulating HCV genotypes, either for epidemiological or diagnostic purposes, require the selection of polymerase chain reaction (PCR) primers that anneal to conserved genomic regions [30]. The genetic regions genotyped vary according to the purpose: 5’untranslated region (UTR), core and NS5B regions for classification purposes and epidemiological studies, hypervariable region-1 (HVR1) of protein E2 for studies on evolution and transmission, NS3-NS5B for DAA drug resistance assessment, and the NS5A interferon sensitivity determining region (ISDR) when investigating treatment response to IFN-containing regimens [31].

Expanding and intensified sequencing efforts worldwide have resulted in an increasing number of HCV genotypic data in public databases, which are useful to update the current knowledge of genetic diversity between and within all HCV genotypes [32]. Studying selective pressure is informative to assess HCV’s potential to escape the immune system and treatment [33]. A more complete analysis of positions important for DAA binding and activity will help assessing the risk for DAA treatment failure either due to pre-existing naturally occurring resistance-related polymorphisms or the ease with which resistance-associated substitutions can be expected [34]. Overall, an integrated map of genome-wide genetic diversity and evolutionary pressure, which highlights their significance for DAA treatment and drug resistance, could contribute to the development of antiviral drugs and drug resistance testing assays.

## 2. Results

### 2.1. HCV Genome-Wide Sequence Diversity

**Figure 1:**
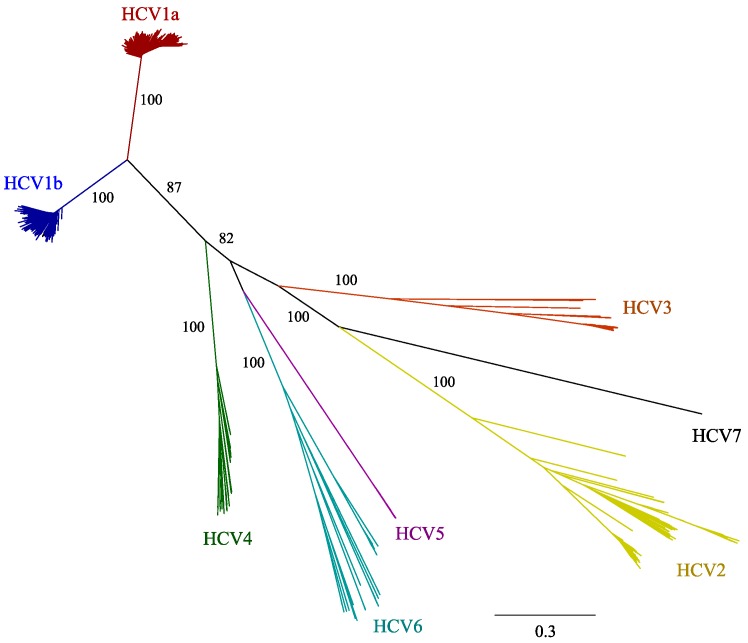
Phylogenetic tree of HCV full-genome sequences. A maximum-likelihood tree of HCV genotypes 1–7 was built using the GTR gamma model of substitution and the robustness of the tree was evaluated using 1000 bootstrap replicates. Bootstrap values above 70% are indicated at each main lineage, and the evolutionary distance scale bar indicates the number of nucleotide substitutions per site along each lineage.

Figure 1 shows the evolutionary relationships between HCV full-genome sequences, with branch lengths proportional to the evolutionary distance. The median within-genotype diversity was 14.55% (IQR: 14.11%–18.65%) at nucleotide level and 9.71% (IQR: 9.51%–11.58%) at amino acid level. The highest nucleotide diversity values were observed for HCV6 (23.37%), followed by HCV4 (20%), HCV3 (14.59%), HCV2 (14.51%), HCV5 (13.97%), and HCV1 (13.53%) (Table S2). Median within-genotype amino acid diversity values are available in Table S3.

Median inter-genotype diversity was 32.39% (IQR: 31.24–33.72) at nucleotide level and 25.02% (IQR: 23.95–28.39) at amino acid level (Table S4). The lowest nucleotide diversity was observed between HCV genotypes 1 and 4 (29.03%), and the highest between HCV genotypes 2 and 3 (35.46%).

**Figure 2:**
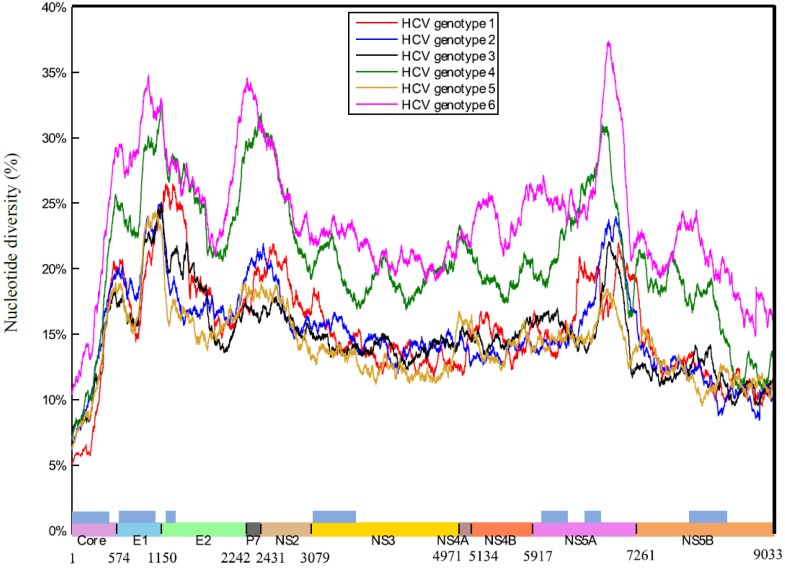
Full-genome sliding window plot for within-genotype nucleotide diversity (%). A sliding window of 300 nucleotide positions with a step size of one nucleotide position was used. The six genotypes were plotted separately in color-coded solid lines (see figure legend). The genomic region of each protein is indicated at the bottom of the figure. Light-blue colored bars indicate genomic regions which are commonly sequenced.

Figure 2 shows genome-wide nucleotide diversity of six HCV genotypes using a sliding window approach. Most commonly sequenced regions are indicated, including the core protein, the HVR1 region of protein E2, and the NS3 and NS5 proteins [30]. However, for the development of appropriate primers, knowledge on shared consensus nucleotides across the six HCV genotypes at specific positions is of additional use (Table S5). Within-genotype amino acid diversity is visualized by a full-genome sliding window plot in Figure S1. Nucleotide and amino acid variability trends were similar across genotypes, with higher diversity within HCV6 and HCV4, although not consistent across individual proteins or protein-coding regions (Tables S2 and S3). The lowest variability was observed for the genetic region encoding the core protein (median diversity NT: 8.72% and AA: 4.36%) while the envelope protein E2 displayed the highest overall diversity (median diversity NT: 20.43% and AA: 18.23%), although for some genotypes the p7 protein is the most variable (Tables S2 and S3).

### 2.2. Frequency of Consensus Nucleotides and Amino Acids

**Figure 3:**
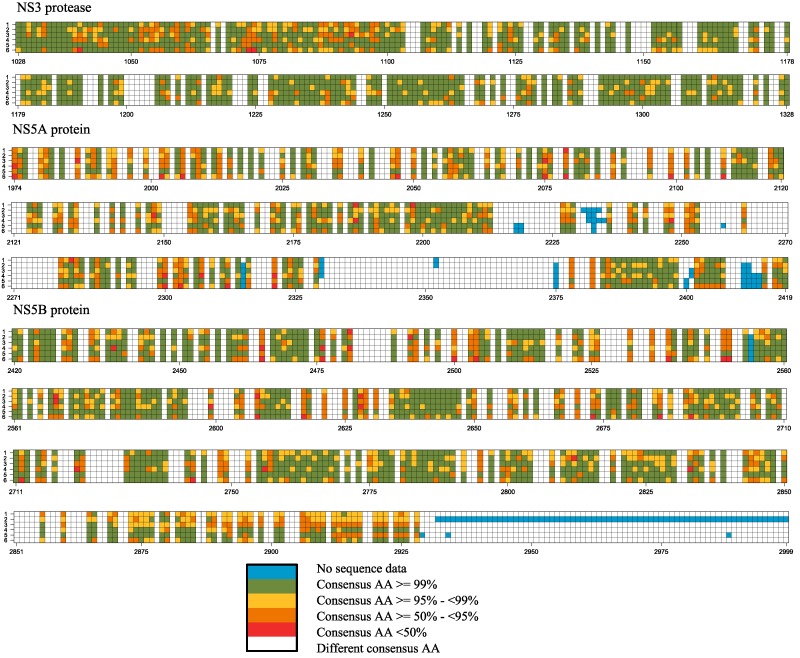
Discretized frequencies of pan-genotypic consensus positions in the NS3, NS5A, and NS5B proteins. The distribution of positions that shared a consensus amino acid across genotypes 1–6, aligned against the reference sequence H77, is shown for HCV proteins NS3, NS5A, and NS5B. Genotype 1 is placed at the top and each square represents a single position. Positions that shared a consensus amino acid across all six genotypes were colored according to the frequency of the consensus amino acid in the respective genotype (for frequency x: category x < 50% in red, 50% ≤ x < 95% in orange, 95% ≤ x < 99% in yellow and x ≥ 99% in green). Positions with different consensus amino acids are colored white and positions with no sequence data or a deletion are indicated in blue. It can be seen that the NS5B of HCV2 genomes are shorter compared to other genotypes.

The consensus amino acid and nucleotide at each position in the full-genome were determined for the genotypes separately. Positions displaying pan-genotypic consensus residues were color-coded according to the four frequency-dependent categories (Figures S2 and S3), as defined in Materials and Methods. Figure 3 shows the amino acid consensus representation for proteins NS3 (more specifically NS3 protease), NS5A, and NS5B. A pan-genotypic consensus position was observed in 56.49% of the 3000 studied genome positions. Based on the frequency-dependent categories, in total, 14.97% of all genome-wide positions was defined as pan-genotypic highly conserved (x≥99% in all six genotypes), and 23.87% as pan-genotypic conserved (frequency of x≥95% in all genotypes but not x≥99% in all genotypes). A detailed position-specific description of consensus residues for each genotype at nucleotide and amino acid level is available in Tables S5 and S6. The proportion of pan-genotypic consensus positions for each viral protein is summarized in Table 1. A high proportion of shared consensus amino acids was observed for the core protein, in contrast to HVR1 in protein E2, where only 37% of the positions shared the same consensus amino acid across all six HCV genotypes (data not shown). Only one of the 27 residues located in HVR1 was defined as pan-genotypic conserved; none were pan-genotypic highly conserved.

**Table 1:** Proportion of pan-genotypic consensus positions in the full-genome and HCV proteins. For each of the ten HCV proteins, as well as for the full-genome, the proportion (%) of positions with a consensus amino acid shared between the six genotypes is indicated. Additionally, proportions of positions defined as pan-genotypic conserved (frequency of x ≥ 95% in all genotypes and 99% > x ≥ 95% in at least one genotype) and as pan-genotypic highly conserved (frequency of x ≥ 99% in all genotypes) were also summarized in the table.

Proportion of Total Positions	Core	E1	E2	p7	NS2	NS3	NS4A	NS4B	NS5A	NS5B	Full-Genome
**Pan-genotypic consensus positions**	80.6	38.5	60.3	25.4	39.6	70.4	53.7	55.6	46.4	55.3	56.5
**Pan-genotypic conserved (95% ≤ x < 99%)**	33.0	14.1	20.7	4.8	15.2	31.4	20.4	27.2	20.4	24.8	23.9
**Pan-genotypic highly conserved (x ≥ 99%)**	31.4	12.5	15.2	6.3	11.1	22.0	14.8	12.3	7.4	12.1	15.0

### 2.3. Positions under Positive Selective Pressure

Selective pressure was analyzed for each genotype separately, using three different methods. When analyzing selective pressure by classifying sites in categories (using the random sites model M2 in NY, see Materials and Methods; comparable conclusions were obtained with the M3 model), we found that the majority (89.66%) of the full-genome sites were classified as under negative selective pressure, while only a median of 0.49% (IQR: 0.36%–3.08%) of sites were classified as positively selected. The highest proportions of positively selected sites were observed for genotypes 1 and 4, with 17% and 5%, respectively.

**Figure 4:**
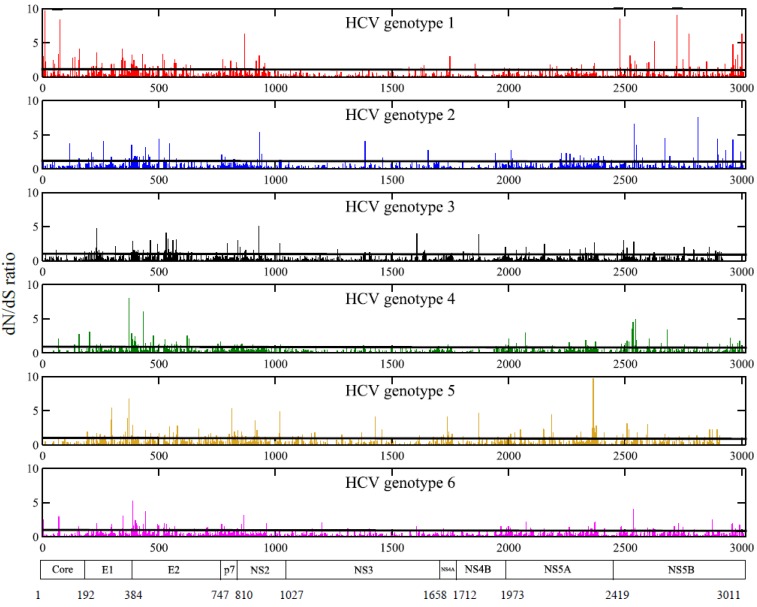
dN/dS ratio at the full-length genome of all six HCV genotypes. Only positions characterized by a dN/dS ratio above 1 (and p-value < 0.05) using SLAC, were defined as positively selected (Table S6). A limited number of positions of the full-genome were identified as positively selected positions. X-axis: amino acid positions along the genome; Y-axis: dN/dS ratio; HCV proteins are shown at the bottom. For each HCV genotype, a line was drawn on the graph to indicate the dN/dS ratio equal to 1.

A lower number of positions under positive selective pressure was also identified using the SLAC model that evaluates significant signal of selective pressure per site (0.23%, IQR: 0.14%–0.47%, Figure 4). While 3.1% of genome positions in HCV1 were being positively selected, positively selected sites in genotype 5 could not be detected. Between proteins, the highest median proportions of positively selected positions were found in envelope proteins E1 (0.52%, IQR: 0.13%–0.42%) and E2 (1.24%, IQR: 0.82%–2.07%), while for small proteins like p7 and NS4A no positively selected sites were found (Table S7). Different sites were identified in different genotypes (Table S8).

Additionally, codon positions in the alignment were evaluated using FEL (see Materials and Methods) to identify positions under positive and negative selective pressure. Overall, evidence of positive selective pressure was detected in 0.46% (IQR: 0.25%–0.71%) of the full-genome positions. Similar trends were observed for HCV genotype 1 (2.16%) and for the individual proteins (E1: 0.78%, E2: 2.07%, p7: 0%, and NS4A: 0%), regarding the number of positively selected sites. Overall 31.70% (IQR: 24.86%–36.13%) of the positions were significantly under negative selective pressure (dN/dS ratio between 0 and 1, and *p*-value < 0.05).

### 2.4. Large-Scale Analysis of Amino Acid Variability at Positions Important for DAA Therapy

Efficient targeting of viral proteins by DAAs requires that key drug binding positions display minimal variability within and between different HCV genotypes. The amino acid distribution at positions known to affect DAA binding, or associated with reduced drug susceptibility, are listed in Table 2, Table 3 and Table 4. Table 5 summarizes all positions harboring a resistance-related amino acid in at least one HCV genotype. Additionally, for all resistance-related positions, the amino acid variability was studied for HCV subtypes 1a and 1b separately (Table 5). Of 27 drug binding positions for NS3/4A protease inhibitors, 13 (48%) were found to be pan-genotypic (highly) conserved, defined by consensus amino acids shared across all genotypes and showing frequencies of at least 95% within each genotype (Table 2). Considering only the 15 resistance-related positions, five (33%) positions were pan-genotypic (highly) conserved, and resistance-related amino acids at six positions were the consensus residues in specific genotypes: 36L (HCV2-5), 80K (HCV5), 122T/R/N (HCV2 and HCV4-6), 168Q/E (HCV3 and 5), 170V (HCV4 and 6), and 175L (HCV1-5).

**Table 2:** Amino acid frequencies at positions important for NS3/4A protease inhibitor drug susceptibility. For each position, the reference sequence H77 amino acid and the distribution of amino acids in each HCV genotype is listed. Frequencies are indicated in superscript, and the amino acids are ranked according to decreasing frequency. Positions where the consensus amino acid is not shared across all six genotypes are shaded in red. Positions defined as pan-genotypic weakly conserved are shaded in yellow, and positions associated with NS3 protease drug resistance are shaded in light grey.

NS3	36	41	42	43	54	55	56	57	80	81	107	117	122	132	136	137	138	139	155	156	157	158	159	168	170	174	175
H77	V	Q	T	F	T	V	Y	H	Q	D	V	R	S	I	K	G	S	S	R	A	A	V	C	D	I	S	L
HCV1	V^97.9^	Q^98.7^	T^6^^0^^.7^	F^99.2^	T^97.3^	V^97.6^	Y^92.4^	H^99.3^	Q^72.1^	D^99.2^	V^99.3^	R^97.5^	S^88.5^	I^7^^0^^.2^	K^99.1^	G^99.3^	S^1^^0^^0^	S^1^^0^^0^	R^98.7^	A^1^^0^^0^	A^99.3^	V^99.3^	C^99.3^	D^98.7^	I^72.3^	S^59.8^	L^61^
	L^1.^^0^	T^0^^.7^	S^38.3^	L^0^^.7^	S^1.9^		F^6.8^			L^0^^.7^	T^0^^.7^	C^1.6^	G^7.6^	V^28.8^	G^0^^.7^	S^0^^.7^			A^0^^.7^		V^0^^.7^	C^0^^.7^		F^0^^.7^	V^27.1^	N^36.3^	M^38.3^
	S^0^^.7^	H^0^^.6^	F^0^^.7^	S^0^^.1^	V^0^^.7^	I^1.2^	H^0^^.7^	G^0^^.7^	K^24.2^	Q^0^^.1^		H^0^^.8^	T^1.8^	L^3.8^	R^0^^.2^				K^0^^.4^				T^0^^.7^	E^0^^.5^	P^0^^.7^	G^2.^^0^	E^0^^.7^
	M^0^^.3^		A^0^^.3^		X^0^^.1^	A^0^^.5^	X^0^^.1^		L^1.9^			Q^0^^.1^	N^1.3^	S^0^^.7^					P ^0^^.2^					G^0^^.1^		A^0^^.8^	X^0^^.1^
	I^0^^.1^					Y^0^^.7^			D^0^^.8^				R^0^^.7^													L^0^^.7^	
									N^0^^.6^				X^0^^.1^													T^0^^.2^	
									R^0^^.3^																	D^0^^.1^	
									M^0^^.1^																	F^0^^.1^	
																										H^0^^.1^	
HCV2	L^99.4^	Q^1^^0^^0^	S^92.6^	F^1^^0^^0^	T^98.2^	V^99.4^	Y^77.8^	H^1^^0^^0^	G^1^^0^^0^	D^1^^0^^0^	V^1^^0^^0^	R^1^^0^^0^	R^77.2^	L^91.4^	K^1^^0^^0^	G^99.4^	S^1^^0^^0^	S^1^^0^^0^	R^1^^0^^0^	A^1^^0^^0^	A^1^^0^^0^	V^95.1^	C^1^^0^^0^	D^1^^0^^0^	I^95.7^	S^72.2^	L^98.1^
	M^0^^.6^		T^7.4^		X^1.2^	G^0^^.6^	F^22.2^						K^21^	I^7.4^		R^0^^.6^						I^3.1^			V^3.1^	T^17.9^	I^1.9^
					A^0^^.6^								T^1.2^	V^0^^.7^								M^1.2^			X^1.2^	A^9.3^	
													X^0^^.7^	X^0^^.7^								A^0^^.6^				M^0^^.6^	
HCV3	L^1^^0^^0^	Q^1^^0^^0^	T^1^^0^^0^	F^1^^0^^0^	T^98.1^	V^1^^0^^0^	Y^1^^0^^0^	H^1^^0^^0^	Q^1^^0^^0^	D^1^^0^^0^	V^94.2^	R^1^^0^^0^	S^1^^0^^0^	L^82.7^	K^1^^0^^0^	G^1^^0^^0^	S^1^^0^^0^	S^1^^0^^0^	R^1^^0^^0^	A^1^^0^^0^	A^1^^0^^0^	V^98.1^	C^96.1^	Q^1^^0^^0^	I^92.3^	T^88.5^	L^1^^0^^0^
					S^1.9^						I^5.8^			I^15.4^								I^1.9^			V^7.7^	A^5.8^	
														V^1.9^									V^3.9^			S^3.8^	
																										X^1.9^	
HCV4	L^1^^0^^0^	Q^1^^0^^0^	S^71.4^	F^1^^0^^0^	T^96.4^	V^1^^0^^0^	Y^98.2^	H^1^^0^^0^	Q^1^^0^^0^	D^1^^0^^0^	V^89.3^	R^1^^0^^0^	T^87.5^	I^94.6^	K^1^^0^^0^	G^1^^0^^0^	S^98.2^	S^1^^0^^0^	R^1^^0^^0^	A^1^^0^^0^	A^1^^0^^0^	V^98.2^	C^1^^0^^0^	D^1^^0^^0^	V^96.4^	S^87.5^	L^1^^0^^0^
			T^28.6^		X^3.6^		X^1.8^				I^7.1^		S^1^^0^^.7^	L^3.6^			F^1.8^					L^1.8^			I^3.6^	A^7.1^	
											X^3.6^		X^1.8^	V^1.8^												N^3.6^	
																										X^1.8^	
HCV5	L^1^^0^^0^	Q^1^^0^^0^	T^1^^0^^0^	F^1^^0^^0^	T^1^^0^^0^	V^66.7^	F^1^^0^^0^	H^1^^0^^0^	K^1^^0^^0^	D^1^^0^^0^	V^1^^0^^0^	R^1^^0^^0^	T^1^^0^^0^	I^1^^0^^0^	K^1^^0^^0^	G^1^^0^^0^	S^1^^0^^0^	S^1^^0^^0^	R^1^^0^^0^	A^1^^0^^0^	A^1^^0^^0^	V^1^^0^^0^	C^1^^0^^0^	E^66.7^	I^66.7^	N^1^^0^^0^	L^1^^0^^0^
						L^33.3^																		D^33.3^	V^33.3^		
HCV6	V^83.9^	Q^1^^0^^0^	S^56.8^	F^1^^0^^0^	T^1^^0^^0^	V^98.8^	Y^9^^0^^.1^	H^97.5^	Q^71.6^	D^1^^0^^0^		R^1^^0^^0^	N^44.4^	I^82.7^	K^98.8^	G^98.8^	S^97.5^	S^1^^0^^0^	R^1^^0^^0^	A^1^^0^^0^	A^1^^0^^0^	V^95.1^	C^1^^0^^0^	D^96.3^	V^58^	N^6^^0^^.5^	M^1^^0^^0^
	L^16.1^		T^43.2^			X^1.2^	F^9.9^		K^25.9^				T^3^^0^^.9^	L^17.3^	R^1.2^	A^1.2^	F^2.5^					I^4.9^		E^3.7^	I^39.5^	S^32.1^	
								Y^2.5^	L^2.5^				S^24.7^												A^2.5^	A^4.9^	
																										G^2.5^	

**Table 3:** Amino acid frequencies at positions important for NS5A inhibitor drug susceptibility. For each position, the reference sequence H77 amino acid and the distribution of amino acids in each HCV genotype is listed. Frequencies are indicated in superscript, and the amino acids are ranked according to decreasing frequency. Positions where the consensus amino acid is not shared across genotypes are shaded in red. Positions defined as pan-genotypic weakly conserved are shaded in yellow, and positions associated with NS5A drug resistance are shaded in light grey.

NS5A Inhibitors	23	28	29	30	31	32	35	36	37	54	56	58	62	92	93	95	97
Reference H77	L	M	P	Q	L	P	P	F	V	H	R	P	E	A	Y	T	P
HCV1	L^99.1^	M^58.5^	P^99.3^	Q^62.5^	L^97.2^	P^99.3^	P^99.3^	F^94.4^	V^57.9^	H^71.5^	R^6^^0^^.6^	H^57.8^	E^6^^0^^.3^	A^98^	Y^97.2^	T^99.^^0^	P^98.4^
	K^0^^.7^	L^37.5^	Q^0^^.7^	R^35.4^	M^2.^^0^	G^0^^.7^	F^0^^.6^	L^4.8^	L^21.8^	Q^24.7^	T^36.7^	P^37.9^	Q^37.2^	Y^0^^.7^	H^1.8^	G^0^^.6^	S^0^^.8^
	I^0^^.1^	V^2.7^		L^0^^.9^	P^0^^.7^		L^0^^.1^	V^0^^.7^	F^15.7^	Y^1.6^	I^1.2^	S^1.3^	D^1.2^	T^0^^.5^	T^0^^.7^	A^0^^.2^	C^0^^.7^
	M^0^^.1^	P^0^^.7^		H^0^^.8^	I^0^^.1^			I^0^^.1^	I^2.3^	N^0^^.9^	C^0^^.7^	C^1^	I^0^^.6^	V^0^^.3^	C^0^^.3^	V^0^^.2^	H^0^^.1^
		T^0^^.3^		K^0^^.3^					M^1.3^	T^0^^.7^	V^0^^.3^	T^0^^.7^	K^0^^.3^	P^0^^.3^			
		I^0^^.2^		M^0^^.1^					S^0^^.7^	L^0^^.4^	N^0^^.2^	Q^0^^.3^	R^0^^.2^	G^0^^.2^			
		F^0^^.1^							Y^0^^.3^	C^0^^.2^	A^0^^.1^	L^0^^.3^	A^0^^.1^	S^0^^.1^			
											S^0^^.1^	N^0^^.2^	S^0^^.1^				
											L^0^^.1^	Y^0^^.2^					
												D^0^^.2^					
												A^0^^.1^					
HCV2	L^1^^0^^0^	L^61.1^	P^99.4^	K^97.5^	M^72.8^	P^1^^0^^0^	P^1^^0^^0^	F^96.3^	I^84.5^	T^98.8^	R^96.3^	P^95.7^	N^85.8^	C^93.8^	Y^1^^0^^0^	E^98.2^	P^58.7^
		F^35.2^	L^0^^.6^	R^2.5^	L^27.2^			L^3.1^	V^12.4^	V^1.2^	K^2.5^	S^3.7^	A^3.7^	S^4.9^		D^0^^.6^	Q^37.7^
		C^1.9^						S^0^^.6^	L^3.1^		Q^1.2^	H^0^^.6^	S^3.1^	A^1.2^		G^0^^.6^	H^1.8^
		I^1.2^											T^3.1^			V^0^^.6^	S^1.2^
		S^0^^.6^											V^1.2^				A^0^^.6^
													D^0^^.6^				
													E^0^^.6^				
													H^0^^.6^				
													L^0^^.6^				
													Y^0^^.6^				
HCV3	L^1^^0^^0^	M^96.2^	P^1^^0^^0^	A^76.9^	L^88.5^	P^1^^0^^0^	P^1^^0^^0^	F^1^^0^^0^	I^71.2^	S^8^^0^^.8^	R^1^^0^^0^	P^98.1^	S^46.1^	E^98.1^	Y^1^^0^^0^	T^96.1^	P^1^^0^^0^
		L^1.9^		K^17.4^	M^7.7^				L^25^	T^19.2^		R^1.9^	T^28.8^	G^1.9^		V^3.9^	
		I^1.9^		L^1.9^	V^3.8^				F^3.8^				M^7.7^				
				S^1.9^									A^3.8^				
				V^1.9^									D^3.9^				
													E^3.9^				
													L^3.9^				
													P^1.9^				
HCV4	L^98.2^	L^83.9^	P^1^^0^^0^	R^68.1^	M^83.9^	P^1^^0^^0^	P^1^^0^^0^	F^1^^0^^0^	L^69.8^	H^1^^0^^0^	T^62.5^	P^85.7^	E^64.4^	A^92.9^	Y^89.2^	T^89.3^	P^92.8^
	X^1.8^	M^1^^0^^.7^		L^1^^0^^.7^	L^16.1^				F^19.6^		V^16.1^	T^1^^0^^.7^	N^8.9^	X^5.4^	H^5.4^	S^1^^0^^.7^	X^3.6^
		I^3.6^		S^1^^0^^.7^					Y^8.9^		I^1^^0^^.7^	R^1.8^	S^8.9^	T^1.8^	R^1.8^		S^1.8^
		V^1.8^		Q^5.4^					I^1.8^		K^7.1^	X^1.8^	Q^7.1^		S^1.8^		A^1.8^
				T^3.4^							Q^1.8^		R^7.1^		T^1.8^		
				A^1.7^							R^1.8^		D^3.6^				
HCV5	L^1^^0^^0^	L^1^^0^^0^	P^1^^0^^0^	Q^1^^0^^0^	L^1^^0^^0^	P^1^^0^^0^	P^1^^0^^0^	F^1^^0^^0^	L^55.6^	S^88.9^	K^1^^0^^0^	P^1^^0^^0^	T^88.9^	A^1^^0^^0^	T^1^^0^^0^	T^1^^0^^0^	P^1^^0^^0^
									F^44.4^	Y^11.1^			A^11.1^				
HCV6	L^1^^0^^0^	V^54.5^	P^1^^0^^0^	S^42.^^0^	L^97.5^	P^1^^0^^0^	P^1^^0^^0^	F^1^^0^^0^	L^58.^^0^	H^81.5^	T^95.^^0^	T^49.4^	V^37.^^0^	A^1^^0^^0^	T^69.2^	T^98.8^	P^1^^0^^0^
		F^22.9^		R^32.1^	I^2.5^				F^23.5^	T^9.9^	K^2.5^	P^45.7^	D^16.1^		S^29.6^	A^1.2^	
		L^2^^0^^.7^		A^23.5^					I^11.1^	N^4.9^	S^2.5^	S^2.5^	Q^12.4^		I^1.2^		
		M^1.9^		N^2.4^					Y^7.4^	R^3.7^		X^2.5^	N^9.9^				
													E^7.4^				
													S^4.9^				
													M^4.9^				
													K^3.7^				
													A^2.5^				
													T^1.2^				

**Table 4:** Amino acid frequencies at positions important for NS5B polymerase inhibitor drug susceptibility. For each position, the reference sequence H77 amino acid and the distribution of amino acids in each HCV genotype is listed. Frequencies are indicated in superscript, and the amino acids are ranked according to decreasing frequency. Positions where the consensus amino acid is not shared across genotypes are shaded in red. Positions defined as pan-genotypic weakly conserved are shaded in yellow, and positions associated with NS5B polymerase drug resistance are shaded in light grey.

NS5B	48	96	149	159	160	162	168	172	220	225	282	291	316	319	321	367	368	386	394	411	414	421	448	495	553	554	556	559
H77	R	S	P	L	I	F	R	K	D	D	S	N	C	D	V	S	S	R	R	N	M	A	Y	P	A	G	S	D
HCV1	R^99.3^	S^99.3^	P^99.2^	L^97.3^	I^99.2^	F^93.3^	R^99.3^	K^99.3^	D^99.2^	D^99.2^	S^99.1^	N^99.3^	C^86.8^	D^99.3^	V^99.7^	S^99.8^	S^99,1^	R^99.^^0^	R^99.1^	N^99.1^	M^99.^^0^	A^88.2^	Y^99.^^0^	P^99.1^	A^93.4^	G^93.5^	S^89.^^0^	D^93.1^
	S^0^^.7^	A^0^^.6^	E^0^^.7^	F^2.^^0^	V^0^^.7^	Y^5.9^	V^0^^.7^	M^0^^.7^	T^0^^.7^	S^0^^.7^	G^0^^.7^		N^12.2^	L^0^^.7^	I^0^^.3^	- ^0^^.2^	N^0^^.7^		A^0^^.7^	I^0^^.7^	F^0^^.7^	V^1^^0^^.^^0^	G^0^^.7^	-^0^^.1^	-^6.1^	-^6.1^	-^6.1^	-^6.5^
		T^0^^.1^	A^0^^.1^	I^0^^.7^	F^0^^.1^	P^0^^.7^			C^0^^.1^	E^0^^.1^	R^0^^.1^	T^0^^.7^	G^0^^.7^				-^0^^.2^	D^0^^.6^	^−^^0^^.2^	-^0^^.2^	-^0^^.2^	-^0^^.8^	-^0^^.2^		G^0^^.4^	Y^0^^.3^	G^3.9^	I^0^^.3^
						S^0^^.1^					T^0^^.1^		H^0^^.1^				P^0^^.1^				I^0^^.1^	R^0^^.7^	C^0^^.1^		V^0^^.1^	X^0^^.1^	N^0^^.7^	N^0^^.1^
													R^0^^.1^					^−^^0^^.2^			V^0^^.1^	T^0^^.2^	H^0^^.1^					
													Y^0^^.1^					H^0^^.1^				M^0^^.1^					D^0^^.3^	
													X^0^^.1^									S^0^^.1^					X^0^^.1^	
																		Y^0^^.1^										
HCV2	R^1^^0^^0^	S^1^^0^^0^	P^1^^0^^0^	L^1^^0^^0^	I^1^^0^^0^	Y^9^^0^^.7^	R^1^^0^^0^	K^1^^0^^0^	D^1^^0^^0^	D^1^^0^^0^	S^1^^0^^0^	N^1^^0^^0^	C^99.4^	D^1^^0^^0^	V^98.1^	S^99.4^	S^99.4^	R^98.8^	R^99.4^	N^97.5^	Q^94.4^	V^81.5^	Y^98.8^	P^98.1^	-^1^^0^^0^	-^1^^0^^0^	-^1^^0^^0^	-^1^^0^^0^
						F^9.3^							W^0^^.6^		F^0^^.6^	- ^0^^.6^	-^0^^.6^		^−^^0^^.6^	T^1.2^	L^4.3^	A^17.3^	X^0^^.6^	-^2.^^0^				
															I^0^^.6^			K^0^^.6^		-^0^^.6^	S^0^^.6^	-^0^^.6^	-^0^^.6^					
															X^0^^.6^			-^0^^.6^		D^0^^.6^	-^0^^.6^	X^0^^.6^						
HCV3	R^1^^0^^0^	S^1^^0^^0^	P^1^^0^^0^	L^1^^0^^0^	I^1^^0^^0^	Y^96.1^	R^1^^0^^0^	K^1^^0^^0^	D^1^^0^^0^	D^1^^0^^0^	S^98.1^	N^1^^0^^0^	C^1^^0^^0^	D^1^^0^^0^	V^1^^0^^0^	S^98.1^	S^1^^0^^0^	R^1^^0^^0^	R^1^^0^^0^	N^96.2^	M^1^^0^^0^	V^1^^0^^0^	Y^1^^0^^0^	P^94.2^	V^71.2^	G^69.2^	G^69.2^	D^71.2^
						F^3.9^					R^1.9^					A^1.9^				S^3.8^				-^5.8^	-^28.8^	-^28.8^S^1.9^	-^28.8^	-^28.8^
																											S^1.9^	
HCV4	R^1^^0^^0^	S^1^^0^^0^	P^1^^0^^0^	L^98.2^	I^1^^0^^0^	Y^89.3^	R^98.2^	K^1^^0^^0^	D^1^^0^^0^	D^1^^0^^0^	S^1^^0^^0^	N^1^^0^^0^	C^75^	D^1^^0^^0^	V^94.6^	S^1^^0^^0^	S^1^^0^^0^	R^1^^0^^0^	R^1^^0^^0^	N^1^^0^^0^	L^48.2^	V^89.3^	Y^1^^0^^0^	P^1^^0^^0^	V^87.5^	G^89.3^	G^8^^0^^.4^	D^87.5^
				X^1.8^		F^1^^0^^.7^	V^1.8^						N^17.9^		I^5.4^						I^25^	A^1^^0^^.7^			-^1^^0^^.7^	-^1^^0^^.7^		
													H^5.4^								V^23.2^				X^1.8^		-^1^^0^^.7^	-^12.5^
													X^1.8^								Q^3.6^						N^7.1^	
																											A^1.8^	
HCV5	R^1^^0^^0^	S^1^^0^^0^	P^1^^0^^0^	L^1^^0^^0^	I^1^^0^^0^	Y^1^^0^^0^	R^1^^0^^0^	K^1^^0^^0^	D^1^^0^^0^	D^1^^0^^0^	S^1^^0^^0^	N^1^^0^^0^	C^1^^0^^0^	D^1^^0^^0^	V^1^^0^^0^	S^1^^0^^0^	S^1^^0^^0^	R^1^^0^^0^	R^66.7^	N^1^^0^^0^	M^1^^0^^0^	A^55.6^	Y^1^^0^^0^	P^66.7^	V^66.7^	G^66.7^	G^66.7^	D^66.7^
																			K^33.3^			V^44.4^		-^22.2^	-^33.3^	-^33.3^		
																								X^11.1^			-^33.3^	-^33.3^
HCV6	R^9^^0^^.1^	S^1^^0^^0^	P^65.4^	L^1^^0^^0^	I^1^^0^^0^	Y^85.2^	R^1^^0^^0^	K^1^^0^^0^	D^1^^0^^0^	D^1^^0^^0^	S^97.5^	N^1^^0^^0^	C^1^^0^^0^	D^1^^0^^0^	V^1^^0^^0^	S^1^^0^^0^	S^92.6^	R^1^^0^^0^	R^1^^0^^0^	N^1^^0^^0^	M^1^^0^^0^	V^1^^0^^0^	Y^1^^0^^0^	P^97.5^	A^64.2^	G^1^^0^^0^	S^6^^0^^.5^	D^1^^0^^0^
			T^11.1^			F^14.8^					C^2.5^						A^7.4^							L^2.5^	S^33.3^		D^3^^0^^.9^	
	K^9.9^		V^8.6^																						V^2.5^			
			S^7.4^																								R^6.2^	
			M^4.9^																								G^2.5^	
			A^2.6^																									

**Table 5:** Overview of NS3, NS5A, and NS5B positions bearing a resistance-related amino acid in at least one genotype. For each resistance-associated variant, its frequency was summarized for all HCV genotypes as well as for subtypes HCV1a and 1b separately. Additionally, the drugs for which the variant was reported to confer drug resistance were listed as well, with the corresponding HCV genotype(s) in which it was first reported, marked in light red.

Variants	HCV1	HCV1a	HCV1b	HCV2	HCV3	HCV4	HCV5	HCV6	DAA
**NS3**									
**V36L**	1%	1.2%	0.5%	99.4%	100%	100%	100%	83.9%	Telaprevir
	1%	1.2%	0.5%	99.4%	100%	100%	100%	83.9%	Asunaprevir
	1%	1.2%	0.5%	99.4%	100%	100%	100%	83.9%	Boceprevir
**Q80K**	24.2%	39.3%	0.25%	0%	0%	0%	0%	0%	Simeprevir
	24.2%	39.3%	0.25%	0%	0%	0%	0%	0%	Asunaprevir
**S122T**	1.8%	0%	4.7%	1.2%	0%	87.5%	100%	30.9%	Simeprevir/Asunaprevir
**S122R**	0.7%	1.1%	0%	77.2%	0%	0%	0%	0%	Simeprevir
**S122N**	1.3%	0%	3.4%	0%	0%	0%	0%	44.4%	Asunaprevir
**D168Q**	0%	0%	0%	0%	100%	0%	0%	0%	Simeprevir
**D168E**	0.5%	0.2%	1%	0%	0%	0%	66.7%	3.7%	Simeprevir/Asunaprevir/Paritaprevir
**I170V**	27.1%	2.8%	65.6%	3.1%	7.7%	96.4%	33.3%	58%	Boceprevir
**M175L**	61%	98%	1%	98.1%	100%	100%	100%	0%	Boceprevir
**NS5A**									
**L28M**	58.5%	94%	2.5%	0%	96.2%	10.7%	0%	1.9%	Daclatasvir
**M28V**	2.7%	4.2%	0.25%	0%	0%	1.8%	0%	54.5%	Daclatasvir/Ombitasvir
**R30Q**	62.5%	97.5%	7%	0%	0%	5.4%	100%	0%	Daclatasvir
**Q30K**	0.3%	0%	1%	97.5%	17.4%	0%	0%	0%	Daclatasvir
**Q30R**	35.4%	0.3%	91%	2.5%	0%	68.1%	0%	32.1%	Daclatasvir/Ledipasvir/Ombitasvir
**L30S**	0%	0%	0%	0%	1.9%	10.7%	0%	0%	Daclatasvir
**L31M**	2%	1.1%	3.4%	72.8%	7.7%	83.9%	0%	0%	Daclatasvir/Ledipasvir
**NS5B**									
**A421V**	10%	12.7%	5.7%	81.5%	100%	89.3%	44.4%	100%	Beclabuvir
**S556G**	3.9%	1.1%	8.4%	0%	69.2%	80.4%	66.7%	2.5%	Dasabuvir

A detailed analysis of the NS5A binding domain I, including positions 33–202, showed that only 24% of the positions were pan-genotypic (highly) conserved (Table 3). For the majority of the residues that were not pan-genotypic conserved, HCV genotypes 2, 4, 5 or 6 most often displayed a different consensus amino acid. Of nine positions reported to be involved in drug resistance, only two positions (22%) were considered pan-genotypic (highly) conserved whereas three individual positions showed a resistance-related amino acid as consensus in some HCV genotypes, namely 28M/V (HCV1 and 3), 30Q/K/R/S (in all HCV genotypes except for HCV3), and 31M (HCV2 and 4).

Regarding the NS5B polymerase inhibitors, 28 drug binding positions were mapped, of which 21 (75%) shared a consensus amino acid and 14 (50%) were in addition pan-genotypic conserved or highly conserved (Table 4). Half of the key drug binding sites (*n* = 14) have been reported as resistance-related positions, of which 29% (4/14) were defined as pan-genotypic (highly) conserved. Moreover, only 2 positions were found in some genotypes as consensus the amino acid was reported to be associated with drug resistance, for instance, 421V in HCV genotypes 2–4 and 6, and 556G in HCV3-5.

## 3. Discussion

The extensive genetic diversity of the hepatitis C virus (HCV) and its potential to rapidly adapt to changing environments can severely limit the performance of diagnostic assays and affect the effectiveness of antiviral drugs and vaccines, thereby hampering efforts to eradicate HCV worldwide. Although HCV diversity has been quantified previously, these studies were limited with respect to the range of genotypes or genomic regions considered, and they did not highlight the impact on DAA treatment [32]. Given the clinical and epidemiological importance especially in this new DAA era, this study aimed to provide a detailed mapping of HCV genomic diversity and to determine the extent of pan-genotype residue conservation, using a large sequence dataset encompassing HCV genotypes 1–6. Variability at amino acid and nucleotide level was estimated for each genotype based on the median number of pairwise differences per site, with an additional correction for amino acids taking into account biochemical similarities [35]. Furthermore, positions that were under positive selection or that shared consensus residues across all genotypes were identified. Given the increasing usage of DAA-based treatment worldwide, amino acid variability at key drug binding and resistance-associated positions was investigated. Our results may give guidance on whether drug resistance testing before the initiation of therapy is needed.

### 3.1. The Highly Diverse Nature of the HCV Genome

HCV is classified into seven genetically distinct HCV genotypes that differ by more than 30% at nucleotide level (Figure 2), with multiple subtypes present within each circulating genotype that are characterized by a diversity of 15%–25% at nucleotide level [5,6]. These estimates emphasize the high genetic variability of HCV, even higher than other genetically diverse viruses such as the Human Immunodeficiency Virus type 1 (HIV-1) and the hepatitis B virus (HBV). HIV-1 group M subtypes differ by 10%–30% at nucleotide level throughout the genome [36,37]. Genotypes within the HBV differ by approximately 8%–10% at nucleotide level and these genotypes are further subdivided into several sub-genotypes and HBsAg subtypes [37,38].

Genome-wide patterns of diversity were similar in all six genotypes, although genotypes 4 and 6 displayed higher overall within-genotype genomic diversity (Figure 1 and Figure 2). In agreement with previous analyses, the core and its encoding genomic region were found to be the least variable, and low diversity values were also obtained for the non-structural proteins NS3, NS4A, NS4B, and NS5B [39]. By contrast, higher diversity estimates were detected for proteins E1, E2, p7, NS2, and NS5A, both at genetic and protein level [5].

Genotype-specific consensus amino acids were shared across all genotypes in more than half of the full-genome positions, and in 39% of all positions the frequency of these consensus amino acids was 95% or higher in each genotype, indicating pan-genotypic conserved positions. For the different proteins, the highest proportion of shared consensus amino acids was detected in the core protein, and the lowest in the p7 protein (Table 1).

Only 0.23%–0.46% of full-genome codon positions were positively selected (Figure 4), in agreement with previous findings [40,41,42,43,44]. The highest number of positions under positive selective pressure was observed in HCV genotype 1 (2.16%–3.12%), however this may be due to a better statistical power given the higher number of strains available. The identity of the positively selected sites differed between genotypes; none of the positively selected positions were pan-genotypic. Highly diverse proteins had the highest number of positively selected positions. Small proteins like p7 and NS4A, but also in general the majority of the full-genome sites, seem to follow the neutral model of evolution, evolving mainly under negative selective pressure and random genetic drift [45]. A slightly higher number of positively selected sites was observed using the random sites models (0.49%), as data were pooled into categories, whereas with SLAC and FEL statistical power per site is lower. As expected, the highest proportion of positively selected positions was located in the variable envelope glycoproteins E1 and E2, consistent with their functional roles in viral escape from immunological responses [46]. Both envelope glycoproteins, targeted by T-and B-cell epitopes, have been identified as key antigens for the development of a preventive vaccine [10]. Overall, in contrast to a higher number of positively selected positions in HIV [36,47], HCV’s high genetic diversity is not heavily influenced by positive selection, but most likely is mainly the result of random genetic drift.

The observed difference in diversity and conservation patterns between the ten HCV proteins is consistent with their function during different stages of the viral life cycle. The HCV core protein forms the viral capsid and interacts with multiple cellular proteins, which may require a high level of conserved residues. Proteins NS3 and NS5B code for viral enzymes, and are involved in the formation of the replication complex through direct interactions with NS4A, NS4B, and NS5A proteins [48,49]. Maintaining enzymatic functions and inter- and intra-protein interactions may require conserved regions in NS3 and NS5B. The envelope proteins have been shown to use glycan shifting as an escape mechanism against neutralizing HCV antibodies, which may possibly account for their high variability [50], as the number of glycosylated positions varies depending on genotype and subtype. Moreover, the highest sequence variability is concentrated in the two hypervariable regions, HVR1 and HVR2 of E2. As described above, these regions are under constant immunological pressure because they are targeted by neutralizing antibodies [51].

### 3.2. Implications for the Development of DAAs

To date, DAAs of three classes have been marketed for treatment of HCV infection, namely NS3/4A protease inhibitors, NS5A inhibitors, and NS5B polymerase inhibitors. In order to develop pan-genotypic drugs, it is important that key drug binding positions are conserved not only within a genotype, but also between different genotypes. Antiviral activity against the six circulating genotypes differs between current drug classes, with first protease inhibitors achieving viral clearance only in genotype 1 infected patients, compared to second-wave compounds that have expanded antiviral activity to some other genotypes. Only second-generation PIs [52], some NS5A inhibitors and some NS5B polymerase nucleotide inhibitors are characterized by pan-genotypic antiviral activity [53]. Despite the improved and expanded treatment options, HCV3 has now become the most difficult-to-treat genotype, even with the new pan-genotypic drugs [54]. Drug designers therefore need improved insights into genetic variability across genotypes at positions relevant for drug activity.

A comparison of consensus amino acids revealed that 48% of positions important for NS3/4A protease inhibitor activity was defined to be pan-genotypic conserved or highly conserved (Table 2), suggesting a rather conserved active site [55] (Table 2). Of the NS3 resistance-related positions, 33% harbored the resistance-associated amino acid as consensus in some genotypes. Compared to the other drug classes, positions targeted by NS5A inhibitors showed the lowest proportion of conserved sites at key drug susceptibility-related positions, in agreement with previous findings [56,57,58,59]. Only 24% of all key drug binding and resistance-related positions could be considered pan-genotypic (highly) conserved (Table 3). In genotypes 2 and 4–6 a different consensus amino acid compared to reference H77 is often seen, which is supported by reports showing that the NS5A inhibitor daclatasvir is a relatively weak inhibitor in HCV genotypes other than HCV1b [60,61]. Among 17 drug binding positions, nine were reported to be associated with drug resistance, of which three positions individually harbored a resistant amino acid. The third class of DAAs, the NS5B polymerase inhibitors, displayed the highest proportion (75%) of key drug susceptibility positions [62] for which all HCV genotypes shared the same consensus amino acid (Table 4), and 50% of these drug binding positions were pan-genotypic conserved or highly conserved. Moreover, weakly conserved positions were mainly found in genotypes HCV5 and HCV6. However, of the fourteen resistance-related NS5B positions identified, only 29% were defined as pan-genotypic (highly) conserved. Yet, in only two positions the drug resistance variant was the consensus, for instance, 421V in HCV genotypes 2–4 and 6 and 556G in HCV3-5.

### 3.3. Implications for Drug Resistance Testing

A sequence diversity analysis at drug resistance-associated positions is important to evaluate the risk of naturally occurring resistance-related variants present at baseline or the risk for development of drug resistance variants under drug selective pressure. As mentioned in the introduction, baseline resistance testing is not routinely performed in HCV clinical practice, although some drugs require a resistance test before treatment initiation. For example, HCV1a infected patients need to be screened for the presence of the 80K polymorphism, before starting treatment with simeprevir [26,27]. For HIV, a cut-off of 5% for the presence of a resistance-related amino acid variant was used to motivate cost effectiveness of testing drug-naïve patients [23]. For HCV, cost-effective studies are not available, but it may be interesting to consider resistance-related polymorphisms at different thresholds, such as 10%, 5%, and 1%, in order to give guidance for resistance testing purposes. Clinicians might be especially interested in Table 5, summarizing the prevalence of all resistance-associated variants in NS3, NS5A, and NS5B for all six HCV genotypes.

Given a cut-off at 10% for resistance testing, it is indeed warranted to screen for the NS3 80K variant prior to treatment with simeprevir in HCV1a-infected patients, which is currently recommended. HCV1a infected patients harboring the 80K variant usually had lower SVR rates on simeprevir treatment, a PI administered to HCV1 and 4 infected patients, compared to patients harboring 80Q [27,63]. In all HCV genotypes, 80Q was the consensus amino acid, except for HCV5, where all sequences displayed 80K. This 80K variant was also observed in HCV1a (39.3%) and HCV6 (25.9%), but to a lesser extent in HCV1b (0.25%). The variable occurrence of the 80K-polymorphism in several genotypes has to be taken into account when a treatment with simeprevir, pegIFN-α, and ribavirin is considered. When a treatment with pan-genotypic NS5A inhibitor daclatasvir is considered in HCV4 infected patients, both resistance-related amino acids 28M and 30S have to be monitored [64], since for 10.7% of these patients achieving good responses with daclatasvir will be possibly hampered due to the presence of these variants. In the case of NS5B polymerase, resistance testing should be considered for variant 421V in HCV1a infected patients when treating with non-nucleoside inhibitor beclabuvir [64], which shows exclusively antiviral activity against HCV1 infections. Our analysis revealed that 12.7% of HCV1a infected patients harbored 421V at baseline, possibly impeding treatment in these patients.

If resistance testing is cost effective at a cut-off of 5%, NS5A variant 30Q should be additionally monitored in HCV1b infected patients treated with NS5A inhibitor daclatasvir [64], since our analysis revealed that 7% of the HCV1b strains harbored amino acid Q. NS5B variant 556G has been reported to confer drug resistance in HCV1b infected patients treated with palm 1 inhibitor dasabuvir [64], a drug that is only active in HCV1 infections. In total, 3.9% of the circulating HCV genotype 1 strains harbored this amino acid, with 8.4% for the HCV1b strains, potentially resulting in suboptimal success rates for this group.

When lowering the threshold to 1%, several additional variants may need to be monitored. HCV1b infected patients, treated either with simeprevir or asunaprevir, may need to be tested for NS3 protease variant 122T [64]. In total, 4.7% of the circulating HCV1b strains harbored this resistance-related amino acid at baseline. HCV1a infected patients considering a treatment with simeprevir, may need to be tested for another NS3 variant on position 122, 122R [64], since this variant was observed in 1.1% of the HCV1a strains. Additionally, NS3 variant 170V has been reported to confer resistance in HCV1a infected patients treated with boceprevir [64], of whom 2.8% bear this amino acid as a naturally occurring resistance-related variant. Before treating HCV1b infected patients with the pan-genotypic NS5A inhibitor daclatasvir, testing for resistance-related amino acid 28M should be considered [64], since achieving good response rates could be hampered for 2.5% of the HCV1b strains. Screening for resistance-related variant 28V may be performed in HCV1a and HCV4 infected patients treated with daclatasvir or ombitasvir [64], both pan-genotypic antivirals, although the latter is only used in the “Viekira Pack” combination in HCV1 and HCV4 infected patients. For both genotypes, the frequency of 28V was, respectively, 4.2% and 1.8%. NS5A variant 31M is associated with drug resistance in HCV1a and HCV1b infected patients treated with daclatasvir, and in HCV1a infected patients treated with ledipasvir [64]. Although this variant was observed as consensus amino acid in HCV genotypes 2 and 4, only 1.1% and 3.4% of the HCV1a and 1b sequences harbored this variant. At this 1% threshold, not only HCV1b, but also HCV1a infected patients should be considered for testing variant 556G when treated with palm 1 inhibitor dasabuvir [64], since 1.1% of the HCV1a strains harbored this amino acid variant.

### 3.4. Implications for the Development of Genotyping Assays and Epidemiological Surveys

The highly variable nature of the hepatitis C virus renders the design of genotyping assays, and particularly the design of primers, a difficult task. Although many assays have been designed for HCV sequencing purposes, evidence of assay validation across genotypes is limited [31]. For resistance testing, genotype-specific assays are often developed, since designing pan-genotypic PCR primers for all six HCV genotypes has so far been too challenging. The genomic region to be sequenced is also dependent on the purpose of the study. Transmission chain investigation should focus on the most divergent regions, like HVR1 in protein E2, in contrast with studies of the origin of the virus, where the most conserved regions are preferred [31]. Identification of highly pan-genotypic conserved genomic regions that border divergent regions like HVR1, can support the design of robust primers to sequence those regions [65].

### 3.5. Limitations

Although all publicly available HCV full-genome sequences were used in this study, HCV1 genotype sequences constituted the majority (75%). A lower number of sequences from other genotypes, in particular for HCV5, could have influenced the reported estimates of diversity and positive selective pressure. The distribution and number of subtypes available for each genotype could further have limited our ability to characterize worldwide HCV diversity. Information on treatment history was largely missing, limiting our analysis on naturally occurring or treatment-associated diversity. However we anticipate that almost all our sequences were DAA naïve because the most recent full-genome sequences in the Los Alamos HCV database date from 2011, with the majority of the sequences prior to 2008. The full-genome sequences collected from the Los Alamos database were primarily obtained using Sanger population sequencing, which cannot detect the presence of natural resistance-associated variants as minor variants, in contrast to deep sequencing approaches [66,67].

## 4. Materials and Methods

### 4.1. Full-Length Genome Sequence Dataset

Full-length HCV genome nucleotide sequences were downloaded from the Los Alamos National Laboratory (LANL) HCV Sequence Database (http://hcv.lanl.gov) [68], resulting in a dataset of 1631 sequences of HCV genotypes 1–6. Genotype 7 was excluded from the analysis, as only one full-length sequence was available at the time. Duplicates and sequences sampled from non-human hosts were discarded, and only one randomly selected sequence per patient was used. Sequence alignment was performed using an in-house developed pairwise alignment tool-chain that checks sequence quality and takes into consideration the different length of the proteins in different genotypes [69]. The genetic region for each of the ten HCV proteins was aligned separately, against reference sequence H77 (NC_004102), and merged into a full-genome alignment. Finally, all alignments were edited manually in Seaview V4.0 [70] and MEGA 6.0 [71] to improve the alignment quality. All position numbering is according to the numbering of the H77 reference sequence. HCV genotype and subtype assignment was verified phylogenetically, using the maximum-likelihood method implemented in RAxML V8.0.20 [72], and using the GTR gamma evolutionary model. Based on the constructed phylogenetic tree, HCV genomic sequences were clustered with all reference sequences retrieved from the LANL database, and five sequences were found to be differently classified in the LANL database. Both the COMET [73] and REGA subtyping tools [36] confirmed the results of our phylogenetic analysis for these sequences, and misclassifications were reported to LANL. Sequences containing stop codons within the open-reading frame (ORF), or only partial information for some of the ten HCV proteins, were removed. The majority of the sequences were classified as genotype HCV1, with 647 HCV1a and 408 HCV1b full-genome sequences. Smaller datasets were collected for the other genotypes, with 162 HCV2, 52 HCV3, 55 HCV4, 9 HCV5, and 82 HCV6 sequences, resulting in a total dataset of 1415 full-genome sequences. The first 3000 codon positions in the constructed full-genome alignment were common to all genotypes and considered for analysis, as protein lengths vary between and within HCV genotypes [69].

### 4.2. Diversity and Consensus Residues

Nucleotide diversity was quantified as the number of nucleotide differences per site in a pairwise comparison [35,74]. Within- and between-genotype diversity was assessed by calculating the median and interquartile range (IQR) over all within- and between-genotype pairwise comparisons, respectively [33]. Diversity estimates were obtained for the full-length genome (excluding the 5’ and 3’ UTRs), and for the genetic regions encoding the ten proteins separately. Diversity was plotted along the genome using a sliding window approach with a window size of 300 nucleotide positions and a step size of one position.

The amino acid sequence was inferred from the nucleotide sequence. Amino acid diversity was quantified using a functional conservation index as defined by Li *et al.* 2013 [74]. For each position, a pairwise similarity matrix was transformed into a functional conservation matrix by taking into account similarities in biochemical properties between residues as defined in the BLOSUM62 substitution matrix, and normalized to values ranging between 0 and 1 [75,76,77]. Finally, amino acid diversity expressed as a percentage (%) was obtained by 1 minus functional conservation index. A value of 0 indicates total amino acid homogeneity while a value of 1 corresponds to the maximum amino acid variation theoretically possible at a specific position. Within- and between-genotype amino acid diversity were assessed by calculating the median and IQR over all within- and between-genotype pairwise comparisons, respectively [74]. The sliding window approach used a window of 100 amino acid positions, with a step size of 1.

Within each genotype, the most occurring amino acid or nucleotide at each position was defined as the consensus residue, even if its frequency was less than 50% [78]. Frequency values at positions that share a consensus residue across all HCV genotypes, defined as pan-genotypic consensus positions, were discretized into four frequency-dependent categories according to threshold values (for frequency x: category 1: x < 50%, category 2: 50% ≤ x < 95%, category 3: 95% ≤ x < 99%, and category 4: x ≥ 99%). A position was defined as pan-genotypic conserved when frequency values of the shared consensus residues were within category 3 or 4 in all genotypes, and as pan-genotypic highly conserved when all frequency values were within category 4. Positions for which categories 1 or 2 were observed in at least one genotype were defined as pan-genotypic weakly conserved.

### 4.3. Positive Selective Pressure

Detection of selective pressure was performed using the methods of single likelihood ancestor counting (SLAC), of fixed effects likelihood (FEL), and of Nielsen and Yang (NY), as implemented in HyPhy v2.2.1 [79,80]. For SLAC, the number of non-synonymous (dN) and synonymous (dS) substitutions at each position was estimated, based on maximum likelihood reconstruction of ancestral codons [81]. Significant differences in the observed and expected proportions of synonymous substitutions were examined using an extended binomial distribution [82]. A position with a dN/dS ratio greater than 1 and a p-value less than 0.05 was considered to be positively selected. For FEL, the assumption that synonymous and non-synonymous rates (dN/dS) vary among sites is premised, iterating through every codon position in the alignment to identify positions under significant positive or negative selection [82]. Positions characterized by a dN/dS ratio > 1 and a *p*-value < 0.05 were defined as positively selected sites. For NY, positively selected sites (category ω_2_) were determined using random sites models M2 (=selection) and M3 (=discrete). Analyzing these random sites models could also define the proportion of sites under negative (category ω_0_) and neutral selective pressure (category ω_1_), with ω representing the ratio dN/dS.

### 4.4. Drug Susceptibility-Related Positions

DAAs that are currently approved for treatment of HCV infection target the NS3/4A, NS5A, and NS5B proteins. NS3/4A protease inhibitors bind either covalently or non-covalently with the catalytic triad of the protease backbone, consisting of three amino acid positions: H57, D81, and S139 [83,84,85]. Near the NS3 catalytic triad, 27 key drug binding positions have been identified at NS3 residues 41–43, 136–138, 155–159, and 168 [86]. Among them, 15 positions have been associated with drug resistance development in patients who have experienced treatment failure with protease inhibitors [63]. For instance, subtype HCV1a infected patients with a virus displaying the Q80K polymorphism showed lower SVR rates upon treatment with simeprevir, compared to patients lacking this polymorphism [27]. As NS5A inhibitors show twofold symmetry, dimeric forms located in NS5A domain I (amino acid positions 33–202) are suggested as potential binding positions, in particular amino acid sites L31, Q54, and Y93 [56,57,58]. In total, 17 NS5A positions were identified as key drug binding positions and nine of them were shown to be involved in drug resistance. The polymerase catalytic site of NS5B is located in the palm domain, and characterized by the conserved glycine-aspartic acid-aspartic acid (GDD) active motif (amino acid positions 317–319) [87]. Both nucleoside inhibitors (NIs) and non-nucleoside inhibitors (NNIs) inhibit the polymerase activity through interaction with multiple residues in the proximity of the active site [88,89,90]. Nucleoside inhibitors only target the active site itself, compared to NNIs which interact with allosteric binding residues located close to the active site. For NS5B, consensus amino acids at 28 key binding positions, and at 14 of which were reported to be associated with drug resistance either *in vitro* or *in vivo*, were examined and compared between all six HCV genotypes.

## 5. Conclusions

A detailed characterization of HCV genomic diversity demonstrated that despite its mainly neutral evolution, only 39% of all amino acid positions were defined as pan-genotypic conserved or highly conserved across all six HCV genotypes. Large differences in sequence variability were observed between the six genotypes, although in general the core was the most conserved region, and envelope proteins the most variable. It has been suggested that HCV evolved mainly under random genetic drift across the entire genome, and codon positions were predominantly found to be under negative selective pressure. Regarding positions involved in drug resistance, NS3 was most affected with resistance-related amino acids in some genotypes, followed by NS5A and NS5B, potentially impeding treatment especially in HCV genotypes 1 and 4. This knowledge is essential for the understanding of HCV epidemiology and for the ongoing developments and improvements of antiviral drugs and diagnostic assays.

## Supplementary Files

Supplementary File 1

## Abbreviations

AA, amino acid; CD4/8, cluster of differentiation 4/8; CI, functional conservation index; COMET, Context-based Modeling for Expeditious Typing; DAA, direct acting antiviral; dN, non-synonymous; dS, synonymous; E2, envelope glycoprotein 2; e.g., exempli gratia (for example); FEL, fixed effects likelihood; GDD, glycine-aspartic acid-aspartic acid; GTR, generalized time-reversible; HAV, hepatitis A virus; HBsAg, hepatitis B virus surface antigen; HBV, hepatitis B virus; HCC, hepatocellular carcinoma; HCV, hepatitis C virus; HIV, human immunodeficiency virus; HVR, hypervariable region; ID, identification; IQR, interquartile range; ISDR, interferon sensitivity determining region; LANL, Los Alamos National Laboratory; Mg, magnesium; NI, nucleo(s)(t)ide inhibitor; NNI, non-nucleoside inhibitor; NS, non-structural; NT, nucleotide; NY, Nielsen and Yang; ORF, open-reading frame; PCR, polymerase chain reaction; pegIFN-α, pegylated interferon-α; RAxML, Randomized Axelerated Maximum Likelihood; RBD, receptor binding domain; RNA, ribonucleic acid; SLAC, single likelihood ancestor counting; SOC, standard-of-care; SVR, sustained virological response; UTR, untranslated region.
