# Supplementary material for: Genetic Diversity and Selective Pressure in Hepatitis C Virus Genotypes 1–6: Significance for Direct-Acting Antiviral Treatment and Drug Resistance
Source: Viruses. 2015 Sep 16;7(9):5018–39. doi: 10.3390/v7092857 (PMC4584301; doi:10.3390/v7092857)
Supplement: Supplementary File 1 [file viruses-07-02857-s001.pdf]

Supplementary Materials

Table S1. Treatment recommendations for each HCV genotype, according to the most recent guidelines [15]. All approved interferon-containing and interferon-free regimens are listed in the table, with the respective HCV genotype in which they can be administered. For the same treatment regimen, the duration of therapy and whether or not ribavirin (RBV) needs to be added to the regimen, can still differ between different genotypes.

	Regimen	Genotype
**IFN-free**	Sofosbuvir + RBV	2 + 3
	Sofosbuvir/Ledipasvir (+ RBV)	1 + 4 + 5 + 6
	Ombitasvir/Paritaprevir/Ritonavir + Dasabuvir (+ RBV)	1
	Sofosbuvir + Simeprevir (+ RBV)	1 + 4
	Sofosbuvir + Daclatasvir (+ RBV)	All
	Ombitasvir/Paritaprevir/r (+ RBV)	4
**IFN-containing**	PegIFN-α + RBV + Sofosbuvir	All
	PegIFN-α + RBV + Simeprevir	1 + 4

Table S2. Within-genotype nucleotide diversity estimates (%) for all six HCV genotypes. Nucleotide diversity is given for each of the genetic regions encoding the ten viral proteins separately, and also for the full genome (excluding UTR). IQR or interquartile range gives a set of values between the 25th percentile (Q1) and the 75th percentile (Q3).

%	Core Genetic Region	E1 Genetic Region	E2 Genetic Region	p7 Genetic Region	NS2 Genetic Region	NS3 Genetic Region	NS4A Genetic Region	NS4B Genetic Region	NS5A Genetic Region	NS5B Genetic Region	Full-Genome (Excluding UTR)
**HCV1**	6.34	17.23	21.51	17.3	18.95	14.16	10.97	14.7	17.56	12	13.53
**HCV2**	8.63	18.63	18.76	18.55	18.38	14.45	15.76	13.36	17.19	11.65	14.51
**HCV3**	8.81	16.5	19.35	17.14	16.43	13.99	13.34	14.45	16.55	11.78	14.59
**HCV4**	9.11	23.79	25.75	26.71	28.32	19.17	20.25	19.57	24.02	16.74	20
**HCV5**	7.99	17.4	17.45	16.8	17.96	12.35	12.89	14.7	15.17	12.04	13.97
**HCV6**	12.63	28.93	26.52	34.49	27.91	21.38	22.98	22.87	27.51	20.35	23.37
**Median**	**8.72**	**18.02**	**20.43**	**17.93**	**18.67**	**14.31**	**14.55**	**14.7**	**17.38**	**12.02**	**14.55**
**IQR (Q1-Q3)**	**8.15–9.04**	**17.27–22.50**	**18.91–24.69**	**17.18–24.67**	**18.07–25.67**	**1.03–17.99**	**13.00–19.13**	**14.51–18.35**	**16.71–22.41**	**11.84–15.57**	**14.11–18.65**

Table S3. Within-genotype amino acid diversity estimates (%) for all six HCV genotypes. Amino acid diversity is given for each of the ten proteins separately, and also for the overall polyprotein. IQR or interquartile range gives a set of values between the 25th percentile (Q1) and the 75th percentile (Q3).

%	Core	E1	E2	p7	NS2	NS3	NS4A	NS4B	NS5A	NS5B	Full-Genome Polyprotein
**HCV1**	2	15	19.01	14.19	15.85	5.31	7	7.04	14.42	7.5	8.71
**HCV2**	4.52	17.17	13.87	15.47	15.42	5.66	10.21	4.61	14.78	8.18	9.6
**HCV3**	4.2	13.98	17.45	14.18	10.92	6	5.08	6.97	12.06	7.63	9.48
**HCV4**	3	17.66	19.85	23.36	24.11	6.75	11.26	7.29	17.56	10.58	12.17
**HCV5**	5.82	14.16	13.95	18.03	15.58	5.46	6.84	9.2	12.46	7.98	9.82
**HCV6**	5.33	25.45	20.91	33.94	21.22	8.78	13.54	13.02	21.57	13.48	15.67
**Median**	**4.36**	**16.09**	**18.23**	**16.75**	**15.72**	**5.83**	**8.61**	**7.17**	**14.60**	**8.08**	**9.71**
**IQR (Q1-Q3)**	**2.13–3.3**	**14.37–17.54**	**14.83–19.64**	**14.51–22.03**	**15.46–19.88**	**5.51–6.56**	**6.88–11.00**	**6.99–8.72**	**12.95–16.87**	**7.72–9.98**	**9.51–11.58**

Table S4. HCV inter-genotype genomic diversity (%). Inter-genotype pairwise nucleotide diversity (upper triangle) and amino acid diversity (bottom triangle) at the genome level.

		NUCLEOTIDE DIVERSITY (%)					
		**HCV1**	**HCV2**	**HCV3**	**HCV4**	**HCV5**	**HCV6**
**AMINO ACID DIVERSITY (%)**	**HCV1**	-	33.66	32.28	29.03	30.85	31.29
	**HCV2**	28.42	-	35.46	33.77	34.36	34.5
	**HCV3**	25.02	29.87	-	32.39	32.79	32.92
	**HCV4**	21.06	28.36	24.98	-	31.09	31.18
	**HCV5**	23.42	29.62	26.25	24.12	-	31.93
	**HCV6**	23.77	28.87	25.57	25.57	24.63	-

Table S5. Nucleotide alignment of the H77 (NC_004102) reference sequence with the consensus of all six HCV genotypes. For each genotype, a consensus alignment was obtained, based on the most frequently occurring nucleotides in the respective datasets. Consensus nucleotides indicated in bold, highlight positions that display a different consensus nucleotide compared to reference sequence H77.

	1 10 20 30 40 50 60 70 79
H77 (NC_004102)	ATGAGCACGAATCCTAAACCTCAAAGAAAAACCAAACGTAACACCAACCGTCGCCCACAGGACGTCAAGTTCCCGGGTG
HCV1	ATGAGCACGAATCCTAAACCTCAAAGAAAAACCAAACGTAACACCAACCGTCGCCCACAGGACGTCAAGTTCCCGGGTG
HCV2	ATGAGCAC**A**AATCCTAAACCTCAAAGAAAAACCAAA**A**G**A**AACAC**A**AACCG**C**CGCCCACAGGACGTCAAGTTCCCGGGTG
HCV3	ATGAGCAC**ACT**TCCTAAACCTCAAAGAAAAACCAAA**A**G**A**AACACCA**T**CCGTCGCCCACAGGACGTCAAGTTCCCGGGTG
HCV4	ATGAGCACGAATCCTAAACCTCAAAGAAAAACCAAACGTAACACCAACCG**C**CGCCC**CAT**GGACGT**T**AAGTTCCCGGGTG
HCV5	ATGAGCACGAATCCTAAACCTCAAAGAAAAACCAAA**A**G**A**AACACCAACCG**C**CGCCCACAGGACGTCAAGTTCCCGGG**C**G
HCV6	ATGAGCAC**ACT**TCC**A**AAACC**C**CAAAGAAAAACCAAA**A**G**A**AACACCAACCGTCGCCCA**AT**GGACGTCAAGTTCCCGGGTG
	80 90 100 110 120 130 140 150 158
H77 (NC_004102)	GCGGTCAGATCGTTGGTGGAGTTTACTTGTTGCCGCGCAGGGGCCCTAGATTGGGTGTGCGCGCGACGAGGAAGACTTC
HCV1	GCGGTCAGATCGTTGGTGGAGTTTACTTGTTGCCGCGCAGGGGCCCTAGATTGGGTGTGCGCGCGACGAGGAAGACTTC
HCV2	GCGG**C**CAGATCGTTGG**C**GGAGTTTACTTG**C**TGCCGCGCAGGGGCCC**C**AG**G**TTGGGTGTGCGCGCGAC**A**AGGAAGACTTC
HCV3	GCGG**A**CAGATCGTTGGTGGAGT**A**TAC**G**TGTTGCCGCGCAGGGGCCC**AC**GATTGGGTGTGCGCGCGACG**C**G**T**AA**A**ACTTC
HCV4	G**T**GG**C**CAGATCGTTGG**C**GGAGTTTACTTGTTGCCGCGCAGGGGCCC**C**AG**G**TTGGGTGTGCGCGCGAC**TC**GGAAGACTTC
HCV5	G**T**GGTCAGATCGTTGGTGGAGTTTACTTGTTGCCGCGCAGGGGCCCTAG**G**TTGGGTGTGCGCGCGAC**TC**GGAAGACTTC
HCV6	GCGGTCAGATCGTTGGTGGAGTTTACTTGTTGCCGCGCAGGGGCCC**CC**GATTGGGTGTGCGCGCGACGAGGAAGACTTC

Table S5. *Cont.*

	159 170 180 190 200 210 220 230 237
H77 (NC_004102)	CGAGCGGTCGCAACCTCGAGGTAGACGTCAGCCTATCCCCAAGGCACGTCGGCCCGAGGGCAGGACCTGGGCTCAGCCC
HCV1	CGAGCGGTCGCAACCTCGAGGTAGACGTCAGCCTATCCCCAAGGCTCGTCGGCCCGAGGGCAGGACCTGGGCTCAGCCC
HCV2	CGAGCGATCCCAGCCGCGTGGGAGACGCCAGCCCATCCCGAAAGATCGGCGCTCCACCGGCAAGTCCTGGGGAAAGCCA
HCV3	TGAACGGTCACAGCCTCGCGGACGGCGACAGCCTATCCCCAAGGCGCGTCGGAGCGAAGGCCGGTCCTGGGCTCAGCCC
HCV4	GGAGCGGTCGCAACCTCGTGGGAGGCGCCAACCTATCCCCAAGGCGCGCCGACCCGAGGGCAGGTCCTGGGCTCAGCCC
HCV5	AGAACGGTCGCAACCCCGTGGACGGCGCCAGCCTATTCCCAAGGCGCGCCAACCCACGGGCCGGTCCTGGGGTCAACCC
HCV6	CGAACGATCCCAGCCCAGAGGCAGGCGCCAGCCTATACCAAAGGCGCGCCAGCCCACGGGCCGGCACTGGGCTCAGCCC
	238 250 260 270 280 290 300 310 316
H77 (NC_004102)	GGGTACCCTTGGCCCCTCTATGGCAATGAGGGTTGCGGGTGGGCGGGATGGCTCCTGTCTCCCCGTGGCTCTCGGCCTA
HCV1	GGGTACCCTTGGCCCCTCTATGGCAATGAGGGCTGCGGGTGGGCGGGATGGCTCCTGTCTCCCCGTGGCTCTCGGCCTA
HCV2	GGATATCCTTGGCCCCTGTACGGAAACGAGGGCTGCGGCTGGGCAGGTTGGCTCCTGTCCCCCCGCGGGTCTCGTCCTA
HCV3	GGGTACCCTTGGCCCCTCTATGGTAACGAGGGCTGCGGGTGGGCAGGGTGGCTCCTGTCCCCACGCGGCTCCCGTCCAT
HCV4	GGGTACCCTTGGCCTCTTTACGGCAATGAGGGCTGCGGGTGGGCAGGATGGCTCCTGTCACCCCGCGGCTCTCGGCCGT
HCV5	GGGTACCCTTGGCCCCTTTACGCCAATGAGGGCCTCGGGTGGGCAGGGTGGTTGCTCTCCCCCCGAGGCTCTCGGCCTA
HCV6	GGTTACCCTTGGCCCCTTTATGGAAACGAGGGCTGCGGGTGGGCAGGGTGGCTCCTGTCCCCCCGCGGCTCCCGCCCAA
	317 330 340 350 360 370 380 390 395
H77 (NC_004102)	GCTGGGGCCCCACAGACCCCCGGCGTAGGTCGCGCAATTTGGGTAAGGTCATCGATACCCTTACGTGCGGCTTCGCCGA
HCV1	GTTGGGGCCCCACAGACCCCCGGCGTAGGTCGCGCAATTTGGGTAAGGTCATCGATACCCTCACGTGCGGCTTCGCCGA
HCV2	CTTGGGGCCCCACTGACCCCCGGCATAGATCACGCAATTTGGGTAAGGTCATCGATACCATTACGTGTGGTTTTGCCGA
HCV3	CTTGGGGCCCAAATGACCCCCGGCGAAGGTCCCGCAATTTGGGTAAAGTCATCGATACCCTCACGTGCGGATTCGCCGA
HCV4	CTTGGGGCCCAAATGATCCCCGGCGGAGGTCGCGCAATTTGGGTAAGGTCATCGATACCCTAACGTGCGGCTTCGCCGA
HCV5	ATTGGGGCCCCAATGACCCCCGGCGAAAATCGCGCAATTTGGGTAAGGTCATCGATACCCTAACGTGCGGATTCGCCGA
HCV6	ATTGGGGCCCCAATGACCCCCGGCGAAGGTCCCGCAATTTGGGTAAGGTCATCGATACCCTAACGTGCGGCTTCGCCGA

Table S5. *Cont.*

	396 400 410 420 430 440 450 460 470 474
H77 (NC_004102)	CCTCATGGGGTACATACCGCTCGTCGGCGCCCCTCTTGGAGGCGCTGCCAGGGCCCTGGCGCATGGCGTCCGGGTTCTG
HCV1	CCTCATGGGGTACATTCCGCTCGTCGGCGCCCCTCTTGGAGGCGCTGCCAGGGCCCTGGCGCATGGCGTCCGGGTTCTG
HCV2	CCTCATGGGGTACATCCCTGTCGTTGGCGCCCCGGTTGGAGGCGTCGCCAGAGCTCTGGCACACGGTGTTAGGGTCCTG
HCV3	CCTCATGGGGTACATCCCGCTCGTCGGCGCTCCCGTAGGAGGCGTCGCAAGAGCCCTCGCGCATGGCGTGAGGGCCCTT
HCV4	CCTCATGGGATACATCCCGCTCGTAGGCGCCCCCGTGGGTGGCGTCGCCAGGGCCCTGGCGCATGGCGTCAGGGCTGTG
HCV5	CCTCATGGGGTACATCCCGCTCGTAGGCGGCCCCGTTGGGGGCGTCGCAAGGGCTCTCGCACACGGTGTGAGGGTTCTT
HCV6	TCTCATGGGGTACATCCCCGTCGTAGGCGCGCCATTGGGCGGCGTCGCGGCTGCCCTCGCACATGGTGTGAGGGCAATC
	475 480 490 500 510 520 530 540 550 553
H77 (NC_004102)	GAAGACGGCGTGAACTATGCAACAGGGAACCTTCCTGGTTGCTCTTTCTCTATCTTCCTTCTGGCCCTGCTCTCTTGCC
HCV1	GAAGACGGCGTGAACTATGCAACAGGGAATCTTCCTGGTTGCTCTTTCTCTATCTTCCTTCTGGCCCTGCTCTCTTGCC
HCV2	GAGGACGGGATAAATTATGCAACAGGGAATCTACCCGGTTGCTCTTTTTCTATCTTCTTGCTTGCTCTTCTGTCGTGCA
HCV3	GAAGACGGGATAAATTTCGCAACAGGGAACTTGCCCGGTTGCTCCTTTTCTATCTTCCTTCTTGCTCTGTTCTCTTGCT
HCV4	GAGGACGGGATCAATTATGCAACAGGGAATCTTCCCGGTTGCTCTTTCTCTATCTTCCTCTTGGCACTTCTCTCGTGCC
HCV5	GAGGACGGGGTAAACTATGCAACAGGGAATTTGCCCGGTTGCTCTTTCTCTATCTTTATCCTTGCACTTCTTTCGTGCC
HCV6	GAGGACGGGATCAATTATGCAACAGGGAATCTTCCCGGTTGCTCTTTCTCTATCTTCCTCTTGGCACTACTCTCGTGCC
	554 560 570 580 590 600 610 620 632
H77 (NC_004102)	TGACTGTGCCCGCTTCAGCCTACCAAGTGCGCAATTCCTCGGGGCTTTACCATGTCACCAATGATTGCCCTAACTCGAG
HCV1	TGACCGTGCCCGCTTCAGCCTACCAAGTGCGCAACTCCTCGGGGCTTTACCATGTCACCAATGATTGCCCTAACTCGAG
HCV2	TCACAGTGCCAGTGTCTGCAGTGGAAGTCAGGAACATCAGTTCTAGCTACTAGGCCACTAATGATTGCTCGAACAACAG
HCV3	TAATTCATCCAGCAGCTAGTCTAGAGTGGCGGAATACGTCTGGCCTCTACGTCCTTACCAACGACTGTTCCAACAGCAG
HCV4	TGACTGTCCCCGCCTCGGCTGTGCACTATCGCAACGCCTCGGGGCACTACCACATCACCAATGACTGCCCGAATTCCAG
HCV5	TGACCGTCCCGGCCTCTGCAGTTCCCTACCGAAATGCCTCTGGGGTTTATCATGTCACCAATGATTGCCCCAACTCTTC
HCV6	TCACAACGCCAGCCTCGGCAGTCCACTACGGCAACACCACTGGGATATACCATCTCACCAATGACTGCCCCAACTCCAG

Table S5. *Cont.*

	633 640 650 660 670 680 690 700 711
H77 (NC_004102)	TATTGTGTACGAGGCGGCCGATGCCATCCTGCACACTCCGGGGTGTGTCCCTTGCGTTCGCGAGGGTAACGCCTCGAGG
HCV1	TATTGTGTACGAGGCGGCCGATGCCATCCTGCACACTCCGGGGTGTGTCCCTTGCGTTCGCGAGGGTAACGCCTCGAGG
HCV2	CATCACCTGGCAGCTCACTGACGCAGTTCTCCACCTTCCTGGATGCGTCCCATGTGAGAATGATAATGGCACCTTGCGC
HCV3	TATTGTGTATGAGGCCGATGACGTTATTCTGCACACACCCGGCTGTGTACCTTGTGTTCAGGACGGCAATACATCCACG
HCV4	CATAGTGTATGAGGCTGACCATCACATCTTGCACCTTCCGGGGTGCGTGCCCTGTGTGAGGACCGGGAACCAGTCGCGA
HCV5	CATAGTCTACGAGGCTGAAAACCTGATCTTACACGCACCTGGTTGCGTGCCCTGTGTCAGGCAAGATAATGTCAGTAGG
HCV6	CATCGTGTATGAGGCGGATGCTATGATCTTGCATCTTCCTGGATGCGTGCCGTGTGTCAGGGTCGGTAATCAGTCCACC
	712 720 730 740 750 760 770 780 790
H77 (NC_004102)	TGTTGGGTGGCGGTGACCCCCACGGTGGCCACCAGGGACGGCAAACTCCCCACAACGCAGCTTCGACGTCATATCGATC
HCV1	TGTTGGGTGGCGCTGACCCCCACGGTGGCCACCAGGGACGGCAAACTCCCCACAACGCAGCTTCGACGTCACATCGATC
HCV2	TGCTGGATACAAGTAACACCTAATGTGGCTGTGAAACACCGCGGTGCACTCACTCACACCCTGCGAACACACGTCGACA
HCV3	TGCTGGACCCCAGTGACACCTACAGTGGCAGTCAGGTACGTCGGAGCAACCACCGCTTCGATACGCAGTCATGTGGACC
HCV4	TGCTGGGTGGCCTTGACCCCCACTGTGGCTGCGCCATACCTCGGTGCTCCACTTGAGTCCATGCGGCGTCATGTGGACC
HCV5	TGCTGGGTCCAAATCACCCCCACACTGTCAGCCCCGAGCTTCGGAGCGGTCACGGCTCCTCTTCGGAGGGCCGTTGACT
HCV6	TGCTGGCATCCTGTGTCCCCCACCCTGGCCGTCCCAAACGCCTCCGTGCCCGCAACGGGGTTCCGCAGGCATGTGGACC
	791 800 810 820 830 840 850 860 869
H77 (NC_004102)	TGCTTGTCGGGAGCGCCACCCTCTGCTCGGCCCTCTACGTGGGGGACCTGTGCGGGTCTGTCTTTCTTGTTGGTCAACT
HCV1	TGCTTGTCGGGAGCGCCACCCTCTGCTCGGCCCTCTACGTGGGGGACCTGTGCGGGTCTGTCTTTCTTGTCGGTCAACT
HCV2	TGATCGTAATGGCAGCTACGGTCTGCTCGGCCTTGTACGTGGGAGATGTGTGCGGGGCCGTGATGATCGCGTCGCAGGC
HCV3	TATTAGTGGGCGCGGCCACGATGTGCTCTGCGCTCTACGTGGGTGATATGTGTGGGGCCGTCTTCCTTGTGGGACAAGC
HCV4	TGATGGTGGGCGCCGCCACTCTGTGCTCCGCCCTTTACGTCGGAGACCTGTGTGGGGGTGTCTTCCTAGTGGGTCAGAT
HCV5	ACTTAGCGGGAGGGGCTGCCCTCTGCTCCGCGCTATACGTCGGAGACGCGTGCGGGGCAGTGTTTTGGTAGGCCAAAT
HCV6	TTCTGGTGGGCGCCGCAGCGGTTTGCTCAGCCCTGTACATCGGGGATCTGTGCGGTGGTGTCTTCCTGGTGGGACAACT

Table S5. *Cont.*

	870 880 890 900 910 920 930 940 948
H77 (NC_004102)	GTTTACCTTCTCTCCCAGGCGCCACTGGACGACGCAAGACTGCAATTGTTCTATCTATCCCGGCCATATAACGGGTCAT
HCV1	GTTCACCTTCTCTCCCAGGCGCCACTGGACGACGCAAGACTGCAATTGTTCTATCTATCCCGGCCATATAACGGGTCAC
HCV2	TTTCATAGTATCGCCACAACACCACAACTTCACCCAAGAGTGCAACTGTTCCATCTACCAAGGTCACATCACCGGCCAC
HCV3	CTTCACGTTCAGACCTCGTCGCCATCAAACGGTCCAGACCTGTAACTGCTCACTGTACCCAGGCCATCTTACAGGACAT
HCV4	GTTCACCTTCCAGCCGCGCCGCCACTGGACCACCCAGGACTGCAATTGTTCCATCTACACTGGCCACATCACAGGCCAC
HCV5	GTTCACCTATAGGCCTCGCCAGCATACTACGGTGCAGGACTGCAACTGTTCCATTTACAGTGGCCATATCACCGGCCAC
HCV6	TTTCACCTTCCGACCCCGCCGCCATCAGACCGTGCAAGACTGCAACTGCTCCATCTATACAGGCCACGTCACCGGCCAC
	949 960 970 980 990 1000 1010 1020 1027
H77 (NC_004102)	CGCATGGCATGGGATATGATGATGAACTGGTCCCCTACGGCAGCGTTGGTGGTAGCTCAGCTGCTCCGGATCCCACAAG
HCV1	CGCATGGCATGGGATATGATGATGAACTGGTCCCCTACAACGGCGCTGGTAGTAGCTCAGCTGCTCCGGATCCCACAAG
HCV2	CGCATGGCATGGGACATGATGATGAACTGGTCACCAACTCTTACCATGATCCTGGCCTACGCTGCTCGTGTTCCTGAGC
HCV3	CGAATGGCTTGGGATATGATGATGAATTGGTCCCCCGCTGTGGGTATGGTGGTGGCGCACGTCCTGCGGTTGCCCCAGA
HCV4	GTTCACCTTCCAGCCGCGCCGCCACTGGACCACCCAGGACTGCAATTGTTCCATCTACACTGGCCACATCACAGGCCAC
HCV5	CGGATGGCATGGGACATGATGATGAATTGGTCACCTACGACAGCCTTGCTGATGGCCCAGTTGTTACGGATTCCCCAGG
HCV6	AGGATGGCTTGGGACATGATGATGAACTGGTCACCCACAGCCACTTATGTCCTCTCTAGCGTCCTGAGAGTACCTCAGA
	1028 1040 1050 1060 1070 1080 1090 1100 1106
H77 (NC_004102)	CCATCATGGACATGATCGCTGGTGCTCACTGGGGAGTCCTGGCGGGCATAGCGTATTTCTCCATGGTGGGGAACTGGGC
HCV1	CCATCTTGGACATGATCGCTGGTGCCCACTGGGGAGTCCTGGCGGGCATAGCGTATTTCTCCATGGTGGGGAACTGGGC
HCV2	TCGTCCTTGAAATCATCTTCGGCGGCCACTGGGGTGTGATGTTTGGCTTGGCCTACTTCTCCATGCAGGGAGCGTGGGC
HCV3	CCTTGTTCGACATAATAGCCGGGGCCCATTGGGGCATCTTGGCGGGCCTAGCCTATTACTCCATGCAGGGCAACTGGGC
HCV4	CCCTGGTTGACATGCTCGCGGGCGGCCACTGGGGCGTCCTGGCTGGCATGGCGTTCTTCAGCATGCAGGCCAATTGGGC
HCV5	TGGTCATTGACATCATTGCCGGGGGCCACTGGGGGGTCTTGTTCGCCGCCGCATACTTCGCGTCAACGGCTAACTGGGC
HCV6	TGCTCCTTGAGATGTTACTTGGTGGCCATTGGGGCGTCCTAGGTGCCGTGGTGTACTTCAGTATGGTGGGCAACTGGGC

Table S5. *Cont.*

	1107 1120 1130 1140 1150 1160 1170 1180 1185
H77 (NC_004102)	GAAGGTCCTGGTAGTGCTGCTGCTATTTGCCGGCGTCGACGCGGAAACCCACGTCACCGGGGGAAGTGCCGGCCGCACC
HCV1	GAAGGTCCTGGTAGTGCTGCTGCTATTTGCCGGCGTCGACGCGGAAACCCACGTCACCGGGGGGACTGCCGCCCGCACC
HCV2	CAAAGTCATTGCCATCCTCCTTCTTGTCGCGGGAGTGGATGCAACCACCTATACCACCGGCGGGCAAGCGGGTCGTACC
HCV3	CAAGGTCGCTATCATCATGGTTATGTTTTCAGGGGTCGATGCCACCACATATACCACCGGTGGCACTGCAGCTCATACT
HCV4	CAAGGTCATCCTGGTCCTGTTCCTCTTTGCTGGGGTTGACGCCAACACCCACGTCACCGGGGGGACCGCGGGCCACACC
HCV5	CAAGGTTGTGCTGGTCTTGTTTCTGTTTGCGGGGGTCGATGCCAACACCCACACTGTGGGCGGTACTGCTGCTCAAAGT
HCV6	AAAGGTCCTGGCTGTTCTGCTCCTCTTTGCAGGGGTTGATGCAACCACCACCACCACCGGCGGCGCAGCGGGCCGCACC
	1186 1190 1200 1210 1220 1230 1240 1250 1260 1264
H77 (NC_004102)	ACGGCTGGGCTTGTTGGTCTCCTTACACCAGGCGCCAAGCAGAACATCCAACTGATCAACACCAACGGCAGTTGGCACA
HCV1	ACGTCTGGGTTTGCGAGCCTCTTCACACCAGGCGCCAAGCAGAACATCCAGCTGATCAACACCAACGGCAGTTGGCACA
HCV2	ACTTCGGGGTTCGCTAGCCTCTTTACCCCTGGTCCCAAGCAGAACATCCAGCTAATCAACACCAATGGCAGCTGGCACA
HCV3	GCCAGCGGGCTTGCTAGCCTTTTCAGTCCGGGCGCCAAACAGAAACTGCAGCTGGTCAACACCAATGGCTCGTGGCACA
HCV4	GCCTCCGGCCTTGCCAGCCTCTTCACCCCGGGGTCCAGGCAGAACCTCCAGCTCATCAACACCAACGGGAGCTGGCACA
HCV5	GCGAGCGCCCTCGCCAGCCTCTTCACCCCTGGGCCGCAGCAGAAGTTGCAGCTCATAAATACCAACGGGAGCTGGCACA
HCV6	GCTTCCGGCCTCACCAGCCTTTTCACCCCCGGTGCCAAGCAGAATCTCCAGCTTATCAACACCAATGGCAGCTGGCACA
	1265 1270 1280 1290 1300 1310 1320 1330 1340 1343
H77 (NC_004102)	TCAATAGCACGGCCTTGAACTGCAATGAAAGCCTTAACACCGGCTGGTTAGCAGGGCTCTTCTATCAGCACAAATTCAA
HCV1	TCAATAGGACGGCCTTGAACTGCAATGACAGCCTCAACACCGGCTGGCTAGCAGGGCTGTTCTATTACCACAAATTCAA
HCV2	TAAACCGGACCGCCCTCAATTGCAATGACAGCTTGCAGACGGGTTTCATCGCTTCCCTGTTTTACGCCAACAAGTTCAA
HCV3	TCAACAGCACTGCCCTGAACTGCAATGATTCCATAAACACCGGGTTCATAGCTGGGTTGTTTTATTATCATAAGTTCAA
HCV4	TCAACAGGACTGCCCTCAATTGCAATGACAGCCTGCACACGGGGTTCATGGCTGGCCTGTTCTACTCCCACAAGTTCAA
HCV5	TCAACAGAACTGCCCTTAATTGTAATGACAGCCTCCAGACTGGGTTTATAGCCGGCCTCATGTATGACCATAAGTTCAA
HCV6	TAAACAGGACTGCTCTGAACTGCAATGATTCCCTCCAGACGGGGTTCATAGCGGGCCTGTTTTACCACCACAAATTCAA

Table S5. *Cont.*

	1344 1350 1360 1370 1380 1390 1400 1410 1422
H77 (NC_004102)	CTCTTCAGGCTGTCCTGAGAGGTTGGCCAGCTGCCGACGCCTTACCGATTTTGCCCAGGGCTGGGGTCCTATCAGTTAT
HCV1	CTCTTCAGGCTGCCCCGAGAGGATGGCCAGCTGCCGACCCCTTGCCGATTTCGCCCAGGGCTGGGGCCCTATCAGTTAT
HCV2	CAGCTCTGGCTGCCCCGAGCGCTTGTCTTCCTGCCGCAGGCTGGACGATTTTCGCATCGGGTGGGGAACCTTGGAATAC
HCV3	CTCTACTGGATGTCCTCAAAGGCTCAGCAGCTGCAAGCCCATCACTTTCTTCAAGCAGGGGTGGGGCCCCTTGACAGAT
HCV4	CAGCTCCGGGTGTCCAGAGAGGCTTGCCAGCTGCAAGCGCCTTGACGACTTCCCACAGGGCTGGGGCCCGCTTGGGGTC
HCV5	CTCCTCTGGGTGTCCAGAGCGGATGGCTAGCTGTAAGCCCCTTGCCGCATTCGACCAGGGCTGGGGAACTATCAGCTAT
HCV6	CTCCTCGGGCTGCCCAGAACGCATGACTGCGTGCAAGCCCCTCGCCTACTTCGACCAGGGGTGGGGCCCAATCACATAT
	1423 1430 1440 1450 1460 1470 1480 1490 1501
H77 (NC_004102)	GCCAACGGAAGCGGCCTCGACGAACGCCCCTACTGCTGGCACTACCCTCCAAGACCTTGTGGCATTGTGCCCGCAAAGA
HCV1	GCCAACGGAAGCGGCCCCGACCAGCGCCCCTACTGCTGGCACTACCCCCCAAAACCTTGTGGTATTGTGCCCGCAAAGA
HCV2	GAGACCACCAACGATGAGGACATGAGGCCGTACTGCTGGCATTACCCCCCGAAGCCTTGCGGTATCGTCCCGGCTAGGA
HCV3	GCTAACGGTCCCTCTGATGAC---AGACCGTACTGCTGGCACTACGCACCTAGACCTTGTGACACTGTCCCGGCATCAA
HCV4	GCTAACGGCCCCTCTGATGAT---AGGCCTTACTGCTGGCACTACGCGCCTCGACCATGCGGGGTGGTGCCCGCATCCA
HCV5	GCCAACGGCCCCAGTGATGAC---AAGCCATATTGCTGGCACTATCCCCCACGACCGTGCGGAGTGGTGCCAGCCCGAG
HCV6	GCCAACGGCCCCTCCGATGAC---AGGCCCTACTGCTGGCATTACGCTCCCAGGCCCTGTGACGTGGTGCCGGCCAGCA
	1502 1510 1520 1530 1540 1550 1560 1570 1580
H77 (NC_004102)	GCGTGTGTGGCCCGGTATATTGCTTCACTCCCAGCCCCGTGGTGGTGGGAACGACCGACAGGTCGGGCGCGCCTACCTA
HCV1	ACGTGTGTGGCCCAGTGTATTGCTTCACTCCCAGCCCCGTGGTGGTGGGAACGACCGACAGGTCGGGCGCGCCTACCTA
HCV2	CGGTTTGCGGGCCGGTCTACTGTTTCACCCCCAGCCCTGTTGTCGTGGGCACCACTGACAGGCAGGGCGTGCCCACCTA
HCV3	GTGTCTGCGGCCCTGTGTACTGCTTCACACCATCGCCAGTGGTCGTAGGCACTACTGATGCTAAGGGCGCCCCAACCTA
HCV4	AAGTGTGTGGCCCCGTGTACTGTTTCACACCCAGCCCTGTGGTGGTCGGCACCACCGACCGGCTCGGGGTCCCCACCTA
HCV5	ATGTCTGCGGTCCAGTCTACTGTTTTACACCTAGCCCAGTGGTCGTTGGCACCACCGACCGCAGAGGGTATCCCACTTA
HCV6	CGGTGTGCGGCCCTGTCTACTGCTTCACACCCAGCCCTGTGGTGGTTGGCACCACTGACAGGAGGGGGAACCCCACATA

Table S5. *Cont.*

	1581 1590 1600 1610 1620 1630 1640 1650 1659
H77 (NC_004102)	CAGCTGGGGTGCAAATGATACGGATGTCTTCGTCCTTAACAACACCAGGCCACCGCTGGGCAATTGGTTCGGTTGTACC
HCV1	CAACTGGGGTGAGAATGAGACGGACGTCTTCGTCCTTAACAACACCAGGCCACCGCTGGGCAATTGGTTCGGTTGTACC
HCV2	CACCTGGGGGGAAAATGAGACCGATGTCTTCCTGCTAAACAGCACAAGACCCCCGCAAGGAGCTTGGTTCGGCTGCACT
HCV3	CACCTGGGGTGAGAATGAGACAGACGTGTTCCTGCTGGAGTCCCTGCGGCCTCCCAGTGGTCGGTGGTTTGGGTGCACG
HCV4	CACCTGGGGGGAGAATGAGACTGATGTGTTCATCCTGAACTCGACCAGGCCTCCCCGAGGCGCGTGGTTCGGGTGCACG
HCV5	CAACTGGGGCGACAATGAGACTGACATCTTTCTCTTGAACAACACGAGGCCCCCTACTGGCAACTGGTTCGGCTGCACC
HCV6	CACCTGGGGGGAGAATGAGACGGATGTGTTCATGCTGGAAAGCCTGCGGCCCCCTACTGGAGGATGGTTCGGGTGCACG
	1660 1670 1680 1690 1700 1710 1720 1730 1738
H77 (NC_004102)	TGGATGAACTCAACTGGATTCACCAAAGTGTGCGGAGCGCCCCCTTGTGTCATCGGAGGGGTGGGCAACAACACCTTGC
HCV1	TGGATGAACTCAACTGGATTCACCAAAGTGTGCGGAGCGCCCCCTTGTGTCATCGGAGGGGTGGGCAACAACACCTTGC
HCV2	TGGATGAACGGGACTGGGTTCACTAAGACATGCGGCGCACCACCTTGCCGCATTAGGAGGGACTTCACTCTCGATCTAT
HCV3	TGGATGAACTCCACGGGGTTTGTCAAGACGTGCGGAGCTCCCCCTTGTAACATCTATGGGGGTCCCGAGTCAGACCTCT
HCV4	TGGATGAACAGCACTGGGTTCACAAAAACCTGCGGGGCCCCTCCATGCAAGGTGCAGACCAAC---AACGACACCTGGA
HCV5	TGGATGAACTCCACAGGGTTTGTGAAGACTTGCGGAGCCCCGCCCTGCAACTTAGGGCCGACA---AACAACAGCCTCA
HCV6	TGGATGAACTCCACGGGCTTTGTCAAGACATGTGGTGCCCCGCCGTGTAAGATAAGCCCCGGAAACAACAACGCGCTTA
	1739 1750 1760 1770 1780 1790 1800 1810 1817
H77 (NC_004102)	TCTGCCCCACTGATTGTTTCCGCAAGCATCCGGAAGCCACATACTCTCGGTGCGGCTCCGGTCCCTGGATTACACCCAG
HCV1	ACTGCCCCACTGACTGCTTCCGCAAGCATCCGGAAGCCACATACTCTCGGTGCGGCTCCGGTCCCTGGATGACACCCAG
HCV2	TGTGCCCCACAGACTGTTTTAGGAAGCACCCAGATGCTACCTATATTAAGTGTGGAGCAGGGCCTTGGTTGACCCCCAG
HCV3	TCTGCCCCACCGACTGCTTCAGGAAACATCCTGAGGCCACATACAGCCGGTGTGGTGCGGGGCCCTGGTTGACACCTCG
HCV4	ACTGCCCCACCGACTGCTTCAGGAAACATCCAGAAACCACCTACACCAAGTGTGGCTCAGGGCCTTGGCTCACGCCAAG
HCV5	AGTGTCCTACTGATTGCTTCCGCAAGCACCCAGACGCCACCTACACCAGGTGTGGGTCAGGACCCTGGCTCACCCCCCG
HCV6	TGTGCCCCACCGACTGCTTCCGTAAACATCCGGAAGCTACATACGCGAGGTGTGGGTCGGGGCCCTGGCTCACTCCCAG

Table S5. *Cont.*

	1818 1830 1840 1850 1860 1870 1880 1890 1896
H77 (NC_004102)	GTGCATGGTCGACTACCCGTATAGGCTTTGGCACTATCCTTGTACCATCAATTACACCATATTCAAAGTCAGGATGTAC
HCV1	GTGCCTGGTCGACTACCCATATAGGCTTTGGCATTATCCTTGTACCGTCAACTACACCATCTTCAAAGTCAGGATGTAC
HCV2	GTGCCTGGTAGACTACCCTTATAGACTGTGGCATTATCCGTGCACTGTAAATTTTACCATCTTCAAGGTGCGGATGTAT
HCV3	GTGCATGGTCGACTACCCATACCGGCTTTGGCATTACCCATGTACAGTCAATTTCACATTGTTCAAGGTGAGGATGTTT
HCV4	GTGCATGGTCCACTACCCATACCGGCTGTGGCACTACCCATGCACTGTCAACTACACCATCTTTAAGGTCAGGATGTAT
HCV5	GTGTTTGGTGCACTACCCTTACCGGTTGTGGCATTACCCGTGCACCGTAAATTATACCATCTTTAAGGTGCGCATGTAC
HCV6	GTGTCTGGTGGACTACCCTTACAGGCTGTGGCACTACCCCTGCACTGTCAACTTCACCATGCACAAGGTCCGGATGTTC
	1897 1910 1920 1930 1940 1950 1960 1970 1975
H77 (NC_004102)	GTGGGAGGGGTCGAGCACAGGCTGGAAGCGGCCTGCAACTGGACGCGGGGCGAACGCTGTGATCTGGAAGACAGGGACA
HCV1	GTGGGAGGGGTCGAGCACAGGCTGGAAGCTGCCTGCAACTGGACGCGGGGCGAGCGTTGTGATCTGGAGGACAGGGACA
HCV2	GTAGGGGGGGTGGAGCATCGGTTCTCCGCAGCATGCAACTTCACGCGCGGGGACCGCTGCAGATTGGAAGACAGGGATA
HCV3	GTGGGTGGGTTTGAACACCGGTTTACCGCCGCTTGCAACTGGACCAGGGGGGAGCGCTGCGATATCGAGGATCGTGACC
HCV4	GTTGGCGGGATAGAGCATCGGCTGGAGGCGGCATGCAACTGGACCAGGGGGGAACCCTGCGGCTTGGAGCACAGAGATC
HCV5	ATTGGGGGCCTAGAGCACAGGCTTGAAGCGGCATGCAACTGGACCCGCGGTGAGCGGTGTGACCTCGAAGACAGAGATA
HCV6	GTGGGGGGCGTTGAGCACAGGTTTGATGCCGCATGCAATTGGACCCGGGGGGAGCGGTGCGAGCTGGATGACAGAGACA
	1976 1980 1990 2000 2010 2020 2030 2040 2050 2054
H77 (NC_004102)	GGTCCGAGCTCAGCCCATTGCTGCTGTCCACCACACAGTGGCAGGTCCTTCCGTGTTCTTTCACGACCCTGCCAGCCTT
HCV1	GGTCCGAGCTCAGCCCGCTGCTGCTGTCCACCACACAGTGGCAGGTCCTTCCGTGTTCCTTCACGACCCTGCCAGCCTT
HCV2	GGGGTCAGCAGAGTCCACTGCTGCATTCCACCACTGAGTGGGCGGTGTTGCCATGCTCCTTCTCCGACCTACCGGCACT
HCV3	GCAGCGAGCAACATCCGCTGCTGCATTCAACAACTGAGCTTGCTATACTGCCTTGCTCTTTCACGCCCATGCCTGCATT
HCV4	GCACAGAGCTGTCGCCCTTGCTCCTTTCCACCACACAGTGGCAGGTCCTCCCCTGCTCCTTCACCACCCTGCCTGCCCT
HCV5	GGGCCGAGCTGAGCCCGCTCCTACACACCACCACACAGTGGGCCATATTGCCGTGCTCTTTCACACCCACGCCCGCTCT
HCV6	GGATTGAGATGAGCCCGCTCCTGTTCTCAACCACGCAGCTTGCCATCCTGCCCTGCTCATTTACCACCATGCCTGCCCT

Table S5. *Cont.*

	2055 2060 2070 2080 2090 2100 2110 2120 2130 2133
H77 (NC_004102)	GTCCACCGGCCTCATCCACCTCCACCAGAACATTGTGGACGTGCAGTACTTGTACGGGGTAGGGTCAAGCATCGCGTCC
HCV1	GTCCACCGGCCTCATCCACCTCCACCAGAACATCGTGGACGTGCAATACTTGTACGGGGTGGGGTCAAGCATTGTGTCC
HCV2	ATCTACTGGCCTATTGCACCTCCACCAAAACATCGTGGACGTGCAGTACCTCTATGGACTTTCTCCGGCTATCACAAGA
HCV3	GTCAACAGGTCTAATACACCTCCACCAAAACATCGTGGATGTCCAATACCTTTATGGTGTTGGATCTGGCATGGTGGGA
HCV4	CTCGACTGGCTTGATCCACCTCCACCAGAACATCGTGGACGTCCAGTACCTTTACGGTGTTGGGTCCGCGGTGGTGTCA
HCV5	TAGCACTGGCCTCATACACTTACATCAGAATATAGTAGACACCCAGTACCTTTACGGCCTGAGCTCCAGCATCGTCTCG
HCV6	ATCAACTGGCCTCATCCACCTGCACCAGAACATAGTGGACGTGCAGTACCTCTACGGAGTCTCCTCGAGCATCGTCTCG
	2134 2140 2150 2160 2170 2180 2190 2200 2212
H77 (NC_004102)	TGGGCCATTAAGTGGGAGTACGTCGTTCTCCTGTTCCTCCTGCTTGCAGACGCGCGCGTCTGCTCCTGCTTGTGGATGA
HCV1	TGGGCCATCAAGTGGGAGTACGTCGTTCTCCTGTTCCTTCTGCTTGCAGACGCGCGCGTCTGCTCCTGCTTGTGGATGA
HCV2	TACATCGTGAAGTGGGAGTGGGTGGTCCTCCTTTTCCTGCTGTTGGCAGACGCCAGGGTCTGTGCATGCCTTTGGATGC
HCV3	TGGGCGTTGAAATGGGAGTTCGTCATCCTCGTTTTCCTCCTCCTAGCAGACGCACGCGTGTGCGTTGCCCTTTGGCTGA
HCV4	TGGGCTCTCAAGTGGGAGTACGTTGTGCTTGCCTTCCTGCTGCTCGCAGACGCCAGGGTCTGTGCGTGCCTGTGGATGA
HCV5	TGGGCCGTTAAGTGGGAGTACATAGTGCTGGCCTTTTTACTACTTGCTGATGCCCGTATCTGTACTTGCCTGTGGATCA
HCV6	TGGGCAGTGAAGTGGGAGTACGTTGTCCTGGTGTTCCTGGTGCTGGCAGATGCTCGCATTTGTGCGTGTCTGTGGTTGA
	2213 2220 2230 2240 2250 2260 2270 2280 2291
H77 (NC_004102)	TGTTACTCATATCCCAAGCGGAGGCGGCTTTGGAGAACCTCGTAATACTCAATGCAGCATCCCTGGCCGGGACGCACGG
HCV1	TGCTACTCATATCCCAAGCGGAGGCGGCTTTGGAGAACCTCGTGGTACTCAATGCAGCATCCCTGGCCGGGACGCACGG
HCV2	TCATCATACTGGGCCAAGCCGAGGCGGCGCTTGAGAAGCTCATCATCTTGCACTCCGCTAGCGCTGCTAGTGCCAATGG
HCV3	TGCTGATGATATCACAAGCAGAAGCAGCCTTGGAGAACCTTGTCACGCTGAACGCCGTCGCTGCTGCCGGGACACATGG
HCV4	TGCTGCTGGTGTCGCAGGTTGAGGCAGCCCTGGCCAACCTGATTACCATCAATGCTGTTTCAGCCGCTGGCACTCACGG
HCV5	TGCTCCTAGTTTGTCAGGCTGAAGCAGCCCTGGAGAACGTCATTGTCCTAAACGCGGCTGCGGCCGCGGGGACTCATGG
HCV6	TGCTGCTCGTAGGCCAAGCTGAGGCAGCACTGGAGAACCTTATCGTCCTCAACGCGGCCTCTGCCGCCAGCACTCACGG

Table S5. *Cont.*

	2292 2300 2310 2320 2330 2340 2350 2360 2370
H77 (NC_004102)	TCTTGTGTCCTTCCTCGTGTTCTTCTGCTTTGCGTGGTATCTGAAGGGTAGGTGGGTGCCCGGAGCGGTCTACGCCTTC
HCV1	TCTTGTGTCCTTCCTCGTGTTCTTCTGCTTTGCCTGGTATCTGAAGGGTAGGTGGGTGCCCGGAGCGGCCTACGCCCTC
HCV2	GCTGCTGTGGTTCTTCATCTTCTTTATAGCGGCCTGGTACTTAAAGGGCAGGGTGGTCCCCGTGGCCACGTACTCTGTT
HCV3	TATCGGCTGGTACCTGGTAGCCTTTTGCGCGGCGTGGCACGTGCGGGGTAAACTTGTCCCGCTGGTGACCTACAGCCTG
HCV4	GTTCTACTACGCCATCCTTTTCATCTGTGTGGCTTGGTATGTCAAGGGCAGGCTCCCAGCTGCTGCCGCCTACGCAGCT
HCV5	GTTCTTCTGGGGTTTGCTCGTCATCTGCTTCGCCTGGCACTTCAAGGGCAGGTTGGTCCCCGGGGCCACCTACCTTTGC
HCV6	CTGGTTGTGGGGGTTGCTTTTCATGTGCTGCGCTTGGTATGTGAAGGGCCGCGTTGTCCCCGCGTTTACCTACGCGGTA
	2371 2380 2390 2400 2410 2420 2430 2440 2449
H77 (NC_004102)	TACGGGATGTGGCCTCTCCTCCTGCTCCTGCTGGCGTTGCCTCAGCGGGCATACGCACTGGACACGGAGGTGGCCGCGT
HCV1	TACGGGATGTGGCCTCTCCTCCTGCTCCTGTTGGCGTTGCCCCAACGGGCATACGCGCTGGACACGGAGGTGGCCGCGT
HCV2	CTCGGCTTGTGGTCCTTTCTCCTCCTAGTCCTGGCCCTGCCACAGCAGGCTTATGCCTTGGACGCCGCTGAACAAGGGG
HCV3	ACGGGTCTTTGGTCCCTAGCATTGCTCGTCCTCTTGCTCCCCCAACGGGCGTATGCTTGGTCGGGTGAAGACAGCGCTA
HCV4	TGCGGGATGTGGCCCCTGCTCCTCCTGCTCCTCGCGCTGCCCGAAAGGGCGTACGCGTTTGACCGGGAGGTGGCAGGGT
HCV5	TTGGGCATTTGGCCACTACTCTTACTCCTTCTCCTCCTGCCCCACAGGGCTCTGGCCCTGGACTCAAGCGATGGCGGGA
HCV6	CTGCAGATGTGGCCCCTCCTGCTGTTAGTCCTGGCCTTGCCTCAGCGGGCATACGCTATGGACAATGAGCAGGCGGCAT
	2450 2460 2470 2480 2490 2500 2510 2520 2528
H77 (NC_004102)	CGTGTGGCGGCGTTGTTCTTGTCGGGTTAATGGCGCTGACTCTGTCGCCATATTACAAGCGCTACATCAGCTGGTGCAT
HCV1	CGTGTGGCGGCGTTGTTCTTGTCGGGTTGATGGCGCTGACTCTGTCACCATATTACAAGCGCTATATCAGCTGGTGCTT
HCV2	AACTAGGGCTGGTCATATTAGTAATCATATCCATCTTTACTCTTACCCCAGCATACAAGATTCTCCTGAGCCGTTCAGT
HCV3	CTCTCGGCGCTGGGGTCTTGGTCCTCTTCGGCTTCTTTACCTTGTCACCTTGGTATAAGCATTGGATCGGCCGCCTCAT
HCV4	CCCTTGGCGGCGGCGTCGTGGTCTTACTGACCATCCTGACATTGTCTCCACATTACAAGCACTGGCTGGCCCGGGGAAT
HCV5	CTGTGGGTTGTCTCGTGCTAACCATTCTTACAATCTTCACACTCACCCCTGGGTATAAGAAGATGGTAGTGCTGGTCAT
HCV6	CCCTCGGGGCTGTTGTTCTTGTCGTTATCACCATCTTCACCCTCACCCCCGCTTACAAGAAGCTGCTCACAAGCTTGAT

Table S5. *Cont.*

	2529 2540 2550 2560 2570 2580 2590 2600 2606
H77 (NC_004102)	GTGGTGGCTTCAGTATTTTCTGACCAGAGTAGAAGCGCAACTGCACGTGTGGGTTCCCCCCCTCAACGTCCGGGGGGG
HCV1	ATGGTGGCTTCAGTATTTTCTGACCAGAGCAGAAGCGCAACTGCACGTGTGGGTTCCCCCCCTCAACGTCCGAGGGGG
HCV2	GTGGTGGCTGTCCTACATGCTGGTCTTGGCCGAGGCCCAGATTCAGCAATGGGTTCCCCCCCTGGAGGCCCGAGGGGG
HCV3	GTGGTGGAACCAGTACACCATATGTAGATGCGAGGCCGCCCTTCAAGTGTGGGTCCCCCCCCTACTTGCACGCGGGAG
HCV4	GTGGTGGTTGCAATACTTCATAGCCAGGGCCGAGGCCATGCTGCATGTCTATGTTCCATCATTGGAGGTGCGCGGCCC
HCV5	GTGGTGGCTTCAGTATTTCATAGCCCGGGTGGAGGCCATCATCCACGTGTGGGTGCCCCCGTTGCAGGTTAGGGGTGG
HCV6	TTGGTGGAACCAGTATTTCATCGCACGAGCTGAGGCCATGCTACACGAGTGGGTGCCCTCCCTACGGGTTAGGGGTGG
	2607–2610 2620 2630 2640 2650 2660 2670 2680 2684
H77 (NC_004102)	GCGCGATGCCGTCATCTTACTCATGTGTGTTGTACACCCGACTCTGGTATTTGACATCACCAAACTACTCCTGGCCAT
HCV1	GCGCGACGCCGTCATCCTACTCATGTGTGTTGTACACCCGGCGCTGGTATTTGACATCACCAAACTGCTGCTGGCCGT
HCV2	GCGTGACGGGATCATCTGGGCGGCTGTCATTCTACACCCACGCCTTGTGTTTGAGATCACGAAATGGTTGTTGGCAAT
HCV3	TAGGGACGGTGTCATCCTGCTAACAAGCCTGCTTTATCCATCTTTAATTTTTGACATCACTAAGCTGCTGATAGCAGT
HCV4	TCGAGACTCCGTCATCATCCTCACGGCCCTGTTCCACCCCCACCTGGCCTTTGACATCACAAAATACCTCCTGGCCAT
HCV5	TCGTGATGCTATCATCATGCTCACATGCCTCTTCCACCCTGCCCTGGGGTTTGAGATCACAAAAATCCTCCTCGGGAT
HCV6	TCGGGACGCCGTCATCCTACTCACCTGCTTGTTACATCCACAGCTGGGGTTTGAGGTCACTAAGATCCTGCTAGCTAT
	2685 2690 2700 2710 2720 2730 2740 2750 2762
H77 (NC_004102)	CTTCGGACCCCTTTGGATTCTTCAAGCCAGTTTGCTTAAAGTCCCCTACTTCGTGCGCGTTCAAGGCCTTCTCCGGAT
HCV1	CTTCGGACCCCTTTGGATTCTCCAAGCCAGTTTGCTTAAAGTGCCCTACTTCGTGCGCGTTCAAGGCCTTCTCCGGAT
HCV2	CCTGGGGCCTGCCTACCTCCTTAAAGCGTCTCTGCTACGGGTGCCATACTTTGTGAGGGCTCACGCTCTGCTACGAGT
HCV3	ATTGGGCCCATTATACTTAATACAGGCTGCCATCACTACCACCCCCTACTTTGTGCGTGCGCATGTACTGGTCCGCCT
HCV4	CCTTGGCCCCCTCTACATACTCCAGGCCTCACTGCTGCGCGTCCCTTACTTTGTGAGGGCTCACGCGCTAATCAAGAT
HCV5	ACTGGGCCCTTTGTACCTGCTGCAGCATTCGCTCACCAAGCTGCCTTACTTCATCAGGGCGCGCGCCCTGCTGAGGCT
HCV6	CCTGGGCCCTCTGTACATCCTCCAGTACAGCTTGCTCAAGGTGCCTTACTTTGTGCGCGCACACATACTCCTGCGCGC

Table S5. *Cont.*

	2763 2770 2780 2790 2800 2810 2820 2830 2840
H77 (NC_004102)	CTGCGCGCTAGCGCGGAAGATAGCCGGAGGTCATTACGTGCAAATGGCCATCATCAAGTTAGGGGCGCTTACTGGCAC
HCV1	CTGCGCGCTAGCGCGGAAGATGGCCGGAGGCCATTACGTGCAAATGGCCATCATCAAGTTGGGGGCGCTTACTGGCAC
HCV2	GTGCACCCTGGTGAGACACCTCGCAGGAGCTAGGTACATCCAGATGCTGCTGATCACCATTGGCAGGTGGACCGGCAC
HCV3	TTGCATGCTCGTGCGCTCCGTGATGGGGGGAAAATACTTCCAGATGGTCATACTGAGCATTGGCAGATGGTTCAACAC
HCV4	CTGCAGCCTGGTGCGCGGGGTGGTGTATGGCAAGTACTGCCAAATGGCCCTACTCAAGGTGGGGGCCCTGACTGGCAC
HCV5	GTGCCTACTAGCAAAGCACCTGGCCTATGGCAAGTACGTGCAGGCGGCCTTGCTCCACCTTGGTAGGCTGACCGGAAC
HCV6	TTGCATGCTTGTCCGTAGGCTAGCAGGGGGTAAGTACGTGCAGGCGTTCCTTCTGAGGCTGGGCGCTTGGACTGGCAC
	2841 2850 2860 2870 2880 2890 2900 2910 2918
H77 (NC_004102)	CTATGTGTATAACCATCTCACCCCTCTTCGAGACTGGGCGCACAACGGCCTGCGAGATCTGGCCGTGGCTGTGGAACC
HCV1	CTATGTTTATAACCATCTCACTCCTCTTCGGGACTGGGCGCACAACGGCCTACGAGATCTGGCCGTGGCTGTAGAGCC
HCV2	TTACATCTATGACCACCTCTCCCCTATGTCAACTTGGGCAGCCCAGGGTTTGCGGGACCTGGCAGTCGCCGTGGAGCC
HCV3	CTACCTATACGACCACCTAGCGCCAATGCAACATTGGGCCGCAGCTGGCCTCAAAGACCTAGCAGTGGCCACTGAACC
HCV4	ATACATCTATGACCACCTCACTCCCTTGTCAGACTGGGCCGCCGACGGCCTTAAGGACCTGGCGGTGGCCCTTGAGCC
HCV5	GTACATTTATGACCACCTTGCCCCCATGAAGGATTGGGCAGCGTCTGGGCTGCGCGACTTAGCGGTGGCCACAGAGCC
HCV6	CTACATCTATGACCATCTCGCCCCCCTTTCTGACTGGGCTAGCGACGGCTTGCGCGATCTGGCCGTTGCAGTTGAGCC
	2919 2930 2940 2950 2960 2970 2980 2990 2996
H77 (NC_004102)	AGTCGTCTTCTCCCGAATGGAGACCAAGCTCATCACGTGGGGGGCAGATACCGCCGCGTGCGGTGACATCATCAACGG
HCV1	AGTCGTCTTCTCCCAAATGGAGACCAAGCTCATCACGTGGGGGGCAGACACCGCCGCGTGCGGTGACATCATCAACGG
HCV2	TGTGGTGTTCAGCCCAATGGAGAAGAAGGTCATTGTGTGGGGGGCTGAGACAGTGGCATGTGGGGACATCCTGCATGG
HCV3	TGTAATATTTAGTCCCATGGAAATCAAGGTCATCACCTGGGGCGCGGACACAGCGGCTTGCGGAGATATTCTTTGCGG
HCV4	AGTCATATTCACGCCCATGGAGAAGAAGGTGATAACCTGGGGCGCTGACACTGCTGCGTGCGGTGACATCATCAAGGG
HCV5	CATCATATTCTCCGCTATGGAGACGAAGGTCATCACGTGGGGGGCTGACACGGCCGCGTGTGGGGACATACTTGCTGG
HCV6	GGTCATTTTCTCTCCCATGGAGAAGAAAGTCATCACCTGGGGTGCTGACACTGCCGCGTGCGGTGACATCCTGACTGG

Table S5. *Cont.*

	2997–3000 3010 3020 3030 3040 3050 3060 3070 3074
H77 (NC_004102)	CTTGCCCGTCTCTGCCCGTAGGGGCCAGGAGATACTGCTTGGGCCAGCCGACGGAATGGTCTCCAAGGGGTGGAGGTT
HCV1	CTTGCCCGTCTCCGCCCGTAGGGGCCGGGAGATACTGCTCGGACCGGCCGATGGAATGGTCTCCAAGGGGTGGAGGTT
HCV2	CCTCCCGGTTTCCGCGAGGCTAGGTAGGGAAGTTCTGCTCGGCCCTGCCGATGGCTACACCTCGAAGGGGTGGAAGCT
HCV3	GCTGCCCGTCTCTGCGCGATTAGGCCGTGAGGTGTTGTTGGGACCTGCTGATGACTATCGGGAGATGGGTTGGCGCCT
HCV4	CCTGCCGGTCTCGGCTAGGCTGGGCAATGAGATCTTGCTCGGCCCGGCTGACACTGAGGCGGCGGGGGGGTGGAGGCT
HCV5	CCTTCCGGTATCAGCTAGACGGGGCCGCGAAATTTTCCTGGGGCCGGCCGACGACATCAAGAAGGCGGGCTGGCGACT
HCV6	CCTCCCTGTGTCAGCTAGGAAGGGGAACCTGGTGCTACTGGGACCCGCTGATGATATGAAGGGCGGGGGTTGGAGGCT
	3075 3080 3090 3100 3110 3120 3130 3140 3152
H77 (NC_004102)	GCTGGCGCCCATCACGGCGTACGCCCAGCAGACGAGAGGCCTCCTAGGGTGTATAATCACCAGCCTGACTGGCCGGGA
HCV1	GCTGGCGCCCATCACGGCGTACGCCCAGCAGACAAGGGGCCTCCTAGGGTGCATAATCACCAGCCTGACAGGCCGGGA
HCV2	CCTAGCTCCCATCACTGCTTACACTCAGCAGACTCGTGGTCTCCTGGGTGCCATCGTGGTCAGCCTAACGGGCCGCGA
HCV3	GTTGGCCCCGATCACAGCATACGCCCAGCAAACTAGGGGCCTTCTTGGGACTATTGTGACCAGCTTGACTGGCAGGGA
HCV4	CCTTGCCCCCATCACCGCGTACGCGCAGCAGACCCGAGGCTTGTTTGGCACCATCGTGACGAGCCTCACCGGCAGAGA
HCV5	TCTCGCCCCTATCACCGCGTATGCGCAGCAGACGCGAGGGGTACTGGGCGCTATAGTCGTCAGCCTAACCGGCCGGGA
HCV6	TCTGGCTCCCATCACCGCGTATGCGCAGCAGACGAGGGGCCTAGTCGGCACCATTGTCACCAGCCTAACCGGCCGTGA
	3153 3160 3170 3180 3190 3200 3210 3220 3230
H77 (NC_004102)	CAAAAACCAAGTGGAGGGTGAGGTCCAGATCGTGTCAACTGCTACCCAAACCTTCCTGGCAACGTGCATCAATGGGGT
HCV1	CAAAAACCAGGTGGAGGGTGAGGTCCAGATTGTGTCAACTGCTGCCCAAACTTTCCTGGCAACCTGCATCAACGGGGT
HCV2	CAAAAATGAGCAGGCTGGGCAGGTCCAGGTTCTGTCCTCCGTCACACAATCTTTCTTGGGGACATCTATTTCGGGGGT
HCV3	TAAGAACGTGGTGACCGGTGAAGTGCAGGTGCTTTCTACGGCTACCCAGACCTTCCTAGGTACAACAGTGGGGGGGGT
HCV4	CACCAATGAGAACTGTGGTGAGGTCCAGGTGCTGTCCACGGCCACGCAGTCCTTCTTGGGTACTGCGATCAATGGCGT
HCV5	CAAAAATGAAGCCGAGGGTGAGGTGCAGGTCCTATCCACGGCAACACAAACATTTCTTGGCACTTCCATCAATGGGGT
HCV6	CAAGAATGAGGTAGAAGGGGAGGTACAGGTGGTCTCCACGGCCACCCAGTCCTTCCTGGCGACCTCCGTCAACGGTGT

Table S5. *Cont.*

	3231 3240 3250 3260 3270 3280 3290 3300 3308
H77 (NC_004102)	ATGCTGGACTGTCTACCACGGGGCCGGAACGAGGACCATCGCATCACCCAAGGGTCCTGTCATCCAGATGTATACCAA
HCV1	GTGCTGGACTGTCTACCACGGGGCCGGAACAAGGACCATCGCATCACCCAAGGGTCCTGTCATCCAGATGTACACCAA
HCV2	CCTCTGGACAGTATACCACGGGGCTGGTAACAAGACCTTGGCTGGCCCCAAAGGACCAGTCACTCAGATGTACACCAG
HCV3	TATGTGGACTGTTTACCATGGTGCAGGTTCAAGGACACTTGCGGGCGCCAAACATCCTGCGCTCCAAATGTACACAAA
HCV4	CATGTGGACTGTGTACCATGGGGCGGGCAGCAAGACCATCAGCGGCCCGAAAGGACCTGTCAACCAAATGTACACCAA
HCV5	AATGTGGACAGTCTTCCACGGGGCCGGCTCTAAGACCCTTGCTGGCCCCAAAGGCCCAGTAGTGCAAATGTACACCAA
HCV6	CCTGTGGACTGTTTATCATGGGGCCGGTTCAAAGACTCTGGCTGGACCGAAAGGACCAGTGTGCCAGATGTACACCAA
	3309 3320 3330 3340 3350 3360 3370 3380 3386
H77 (NC_004102)	TGTGGACCAAGACCTTGTGGGCTGGCCCGCTCCTCAAGGTTCCCGCTCATTGACACCCTGCACCTGCGGCTCCTCGGA
HCV1	TGTAGACCAAGACCTCGTGGGCTGGCCCGCTCCCCAAGGTGCCCGCTCATTGACACCCTGCACCTGCGGCTCCTCGGA
HCV2	CGCAGAGGGGGACCTCGTGGGATGGCCTAGCCCCCCCGGGACTAAGTCATTAGACCCCTGTACCTGCGGGGCCGTGGA
HCV3	TGTAGATCAGGACCTCGTTGGGTGGCCAGCCCCTCCAGGGGCTAAGTCTCTTGAACCGTGCACCTGCGGGTCTGCAGA
HCV4	TGTTGACCAAGACCTAGTGGGATGGCCAGCCCCCCCGGGGGTCAAGTCTCTGGCGCCGTGCACCTGCGGCGCATCGGA
HCV5	TGTAGACAAAGACCTGGTGGGATGGCCTTCCCCCCCCGGAACGCGCTCGCTCACTCCGTGTACTTGCGGATCGGCGGA
HCV6	TGTGGACCAGGACCTGGTAGGATGGCCAGCTCCCCCGGGAGCAAGGTCGCTCACACCATGCACTTGTGGCTCTAGTGA
	3387 3390 3400 3410 3420 3430 3440 3450 3460 3464
H77 (NC_004102)	CCTTTACCTGGTCACGAGGCACGCCGATGTCATTCCCGTGCGCCGGCGAGGTGATAGCAGGGGTAGCCTGCTTTCGCC
HCV1	CCTTTACCTGGTCACGAGGCACGCCGATGTCATTCCCGTGCGCCGGCGGGGTGATAGCAGGGGCAGCCTGCTTTCGCC
HCV2	CCTCTACCTGGTCACCCGAAACGCTGATGTCATTCCGGTCCGGAGGAAAGATGACCGGCGGGGTGCACTACTCTCGCC
HCV3	CTTATACTTGGTTACCCGCGATGCTGATGTCATCCCTGCTCGGCGCAGGGGGGACTCCACAGCGAGCTTGCTCAGTCC
HCV4	CCTGTTCCTGGTTACCAGGCACGCAGACGTGGTGCCCGTGCGCAGGAGGGGCGACACCCGGGGCGCCCTCCTGAGCCC
HCV5	TCTGTACCTGGTTACCAGGCATGCAGACGTCATTCCCGCCCGTAGACGGGGCGACACTCGCGCCTCCCTGCTCAGCCC
HCV6	CCTCTATCTGGTCACGAGGAATGCCGATGTCATCCCTGCAAGGCGCAGGGGTGACAACCGTGCTGCCCTCCTCAGTCC

Table S5. *Cont.*

	3465 3470 3480 3490 3500 3510 3520 3530 3542
H77 (NC_004102)	CCGGCCCATTTCCTACTTGAAAGGCTCCTCGGGGGGTCCGCTGTTGTGCCCCGCGGGACACGCCGTGGGCCTATTCAG
HCV1	CCGGCCCATCTCCTACTTGAAAGGCTCCTCGGGGGGTCCGCTGCTGTGCCCCGCGGGACACGCCGTGGGCATATTCAG
HCV2	AAGGCCTCTCTCAACCCTCAAAGGATCATCCGGCGGACCCGTGCTCTGCCCTAGGGGACACGCCGTGGGCTTGTTCAG
HCV3	TAGGCCTCTCGCCTGTCTCAAAGGTTCCTCTGGAGGTCCTGTTATGTGCCCTTCGGGGCATGTTGCGGGGATCTTTAG
HCV4	CAGGCCAATCTCCACCCTCAAGGGATCCTCTGGCGGTCCGCTGCTGTGCCCCCTGGGACACGCCGTCGGCATATTCCG
HCV5	GAGACCCATCAGTTATCTCAAGGGTTCCTCGGGCGGCCCGGTCATGTGCCCTTCCGGGCATGTGGTAGGTGTTTTCAG
HCV6	ACGGCCCATAAGCACCCTGAAAGGGTCCTCGGGAGGCCCCATGCTGTGCCCCTCGGGCCACGTTGTGGGACTCTTCCG
	3543 3550 3560 3570 3580 3590 3600 3610 3620
H77 (NC_004102)	GGCCGCGGTGTGCACCCGTGGAGTGGCTAAGGCGGTGGACTTTATCCCTGTGGAGAACCTAGAGACAACCATGAGATC
HCV1	GGCCGCGGTGTGCACCCGTGGAGTGGCTAAGGCGGTGGACTTTATCCCCGTGGAGACCCTAGAGACAACCATGAGGTC
HCV2	AGCGGCCGTGTGCTCCAGGGGTGTGGCCAAATCCATTGACTTCATCCCTGTTGAGTCTCTCGACATCGCCACACGGTC
HCV3	GGCTGCTGTGTGCACCAGAGGTGTAGCAAAAGCCCTACAGTTCATACCAGTGGAAACCCTTAGCACACAGGCTAGGTC
HCV4	AGCCGCGGTGTGCACCCGGGGCGTGGCTAAAGCAGTGGACTTCGTGCCGGTTGAGTCTCTTGAAACCACCATGAGGTC
HCV5	GGCTGCCGTGTGCACGCGGGGTGTCGCGAAGGCGCTCGAGTTCATCCCGGTCGAAAACCTGGAGACCACGATGCGCTC
HCV6	CGCTGCCGTATGCACAAGGGGTGTGGCAAAATCCCTTGATTTTGTCCCAGTGGAGAACATGGAGACCACCATGCGCTC
	3621 3630 3640 3650 3660 3670 3680 3690 3697
H77 (NC_004102)	CCCGGTGTTCACGGACAACTCCTCTCCACCAGCAGTGCCCCAGAGCTTCCAGGTGGCCCACCTGCATGCTCCCACCG
HCV1	CCCGGTGTTCACGGACAACTCCTCTCCACCAGCAGTGCCCCAGAGCTTCCAGGTGGCCCACCTGCATGCTCCCACCG
HCV2	GCCCAGTTTCTCTGACAACAGCACGCCACCAGCTGTGCCCCAGACCTACCAGGTGGGCTACTTGCATGCACCAACAG
HCV3	TCCATCTTTCTCTGACAATTCAACTCCTCCTGCTGTCCCACAGAGCTATCAAGTAGGGTACCTTCATGCCCCGACCG
HCV4	CCCGGTCTTCACTGACAACTCCACGCCCCCTGCTGTGCCCCAGACCTACCAGGTCGCCCACTTGCACGCACCGACGG
HCV5	GCCTGTGTTCACTGACAACAGCACACCCCCTGCGGTCCCTCATGAGTTCCAAGTGGGGCACCTCCATGCGCCCACCG
HCV6	TCCCTCCTTCACAGACAACTCCACGCCGCCTGCTGTGCCCCAGACCTACCAGGTGGGGTACCTCCACGCACCAACAG

Table S5. *Cont.*

	3698–3700 3710 3720 3730 3740 3750 3760 3770 3774
H77 (NC_004102)	GCAGCGGTAAGAGCACCAAGGTCCCGGCTGCGTACGCAGCCCAGGGCTACAAGGTGTTGGTGCTCAACCCCTCTGTT
HCV1	GCAGCGG**C**AAGAGCACCAAGGTCCCGGCTGC**A**TACGCAGCCCAGGGCTACAAGGTG**C**T**A**GTGCTCAACCCCTC**C**GTT
HCV2	GCAGCGG**G**AAGAGCACCAAGGTCCC**T**GC**C**GC**A**TA**T**GC**CAGT**CAGGG**G**TA**T**AA**A**GT**AC**T**C**GT**A**CT**A**AA**T**CCCTCTGT**C**
HCV3	GCAGCGGTAAGAGCAC**A**AAGGTCCCGGC**C**GC**T**TA**T**G**T**AGC**A**CA**A**GG**A**TA**T**AA**T**GT**TC**T**C**GTG**T**T**G**AA**T**CC**A**TC**G**GT**G**
HCV4	G**G**AGCGG**C**AAGAGCACCAA**A**GTCCCGGC**C**GCGTA**T**GC**G**GCCCAGGGCTACAAGGTG**C**T**A**GTGCTCAACCCCTC**G**GTT
HCV5	G**G**AGCGG**G**AAGAGCAC**G**AAGGT**A**CC**C**GCTGC**T**TACGC**G**GCCCA**A**GG**G**TACAAGGTG**C**TGGTGCT**A**AACCC**G**TC**C**GT**C**
HCV6	GCAGCGG**G**AAGAGCACCAAGGTCCCGGC**A**GCGTA**T**GC**T**GC**T**CAGGGCTACAAGGTG**C**TGGTGCT**G**AACCC**A**TC**G**GT**G**
	3775 3780 3790 3800 3810 3820 3830 3840 3851
H77 (NC_004102)	GCTGCAACGCTGGGCTTTGGTGCTTACATGTCCAAGGCCCATGGGGTTGATCCTAATATCAGGACCGGGGTGAGAAC
HCV1	GCTGCAAC**A**CTGGGCTTTGGTGCTTACATGTCCAAGGCCCATGGG**A**T**C**GATCCTAA**C**ATCAGGAC**T**GGGGTGAG**G**AC
HCV2	GC**G**GC**C**AC**A**CT**T**GG**T**TTTGG**G**GC**C**TACATGTCCAA**A**GCCCA**C**GGG**A**T**CA**A**C**CC**C**AA**C**ATCAG**A**ACCGG**A**GT**AC**G**G**AC
HCV3	GC**G**GC**C**AC**A**CT**A**GGCTT**C**GG**CT**CTT**T**CATGTC**GCGT**GCC**T**ATGGG**A**T**C**GA**C**CC**C**AA**C**ATC**C**G**C**AC**T**GGG**AACC**G**C**AC
HCV4	GCTGC**G**AC**A**CTGGGCTTTGG**G**GC**A**TACATGTCCAAGGC**G**CATGG**CA**T**C**GA**C**CC**G**AA**C**AT**AC**GG**T**C**A**GG**T**GT**C**AG**G**AC
HCV5	GC**A**GC**G**ACGCT**C**GG**G**TT**C**GG**G**GC**A**TACATGTC**A**A**GA**GC**AT**ATGG**C**GT**G**GA**C**CC**C**AA**C**ATCAGGACCGG**A**GT**T**AG**G**AC
HCV6	GC**G**GC**C**ACGCT**T**GGCTTTGG**C**GCTTA**T**ATG**AGT**AA**A**GC**T**CATGG**CA**T**A**GA**C**CC**G**AA**C**ATC**C**GGACCGGGGT**C**AG**G**AC
	3852 3860 3870 3880 3890 3900 3910 3920 3928
H77 (NC_004102)	AATTACCACTGGCAGCCCCATCACGTACTCCACCTACGGCAAGTTCCTTGCCGACGGCGGGTGCTCAGGAGGTGCTT
HCV1	AATTACCACTGGCAGCCCCATCACGTACTCCACCTACGGCAAGTTCCTTGCCGACGGCGGGTGCTCAGG**G**GGTGCTT
HCV2	**TG**T**G**ACCAC**C**GG**GGA**CCCCATCAC**C**TACTCCAC**T**TA**T**GGCAAGTT**T**CT**C**GC**A**GA**T**GG**A**GG**C**TGCTCAG**CT**GG**C**GC**C**T
HCV3	**CG**TTAC**A**ACTGG**TGCTAAAC**T**G**AC**C**TA**T**TCCACCTACGG**T**AAGTT**T**CT**C**GC**G**GACGG**G**GG**T**TGCTC**C**GG**G**GG**A**GC**A**T
HCV4	**C**AT**C**ACCAC**A**GG**CGCG**CC**T**ATCAC**A**TACTCCACCTACGG**G**AAGTTCCT**G**GCCGA**T**GG**A**GG**T**TGC**GGG**GGAGG**A**GC**G**T
HCV5	**TG**T**C**ACCAC**C**GG**AGCTG**C**T**ATCAC**A**TACTC**T**AC**T**TA**T**GGCAAGTTCCT**C**GC**T**GACGG**G**GGGTGCTCAGG**C**GG**C**GC**G**T
HCV6	**C**AT**C**ACCAC**A**GG**CG**GC**G**C**T**ATCAC**C**TACTCCAC**A**TA**T**GG**G**AA**A**TTCCT**G**GCCGA**T**GG**G**GG**A**TG**T**TC**T**GG**G**GG**G**GC**G**T

Table S5. *Cont.*

	3929 3940 3950 3960 3970 3980 3990 4000 4005
H77 (NC_004102)	ATGACATAATAATTTGTGACGAGTGCCACTCCACGGATGCCACATCCATCTTGGGCATCGGCACTGTCCTTGACCAA
HCV1	ATGACATAATAATTTGTGACGAGTGCCACTCCACGGATGCCACATCCATCTTGGGCATCGGCACTGTCCTTGACCAA
HCV2	ATGACATCATCATATGCGATGAATGCCATTCAGTGGACGCTACTACCATCCTTGGCATTGGAACAGTCCTTGACCAG
HCV3	ATGATGTGATTATCTGTGATGAATGTCATGCCCAAGACGCTACTAGCATATTGGGTATAGGCACGGTCTTAGATCAG
HCV4	ATGACATCATCATCTGTGACGAGTGCCATTCCACTGATTCCACAACGATCCTTGGCATAGGCACGGTCCTGGACCAA
HCV5	ATGACGTGATCATATGCGACGAGTGCCATTCCCAGGACGCTACCACCATCCTTGGGATAGGCACAGTCCTTGACCAG
HCV6	ATGACATCATCATCTGTGACGAGTGCCACTCAACAGACCCTACCACGGTCCTGGGCATAGGCACGGTTCTCGACCAG
	4006 4010 4020 4030 4040 4050 4060 4070 4082
H77 (NC_004102)	GCAGAGACTGCGGGGGCGAGACTGGTTGTGCTCGCCACTGCTACCCCTCCGGGCTCCGTCACTGTGTCCCATCCTAA
HCV1	GCAGAGACTGCGGGGGCGAGACTGGTCGTGCTCGCCACCGCTACCCCTCCGGGCTCCGTCACCGTGCCCCATCCTAA
HCV2	GCTGAGACCGCAGGTGTCAGGCTAGTGGTTTTGGCCACAGCCACGCCTCCCGGTACGGTGACAACTCCCCACACCAA
HCV3	GCTGAGACGGCTGGGGTGAGGCTGACGGTTTTAGCGACAGCAACTCCCCCAGGCAGCATCACTGTGCCACATTCTAA
HCV4	GCGGAGACCGCTGGAGCGCGCCTTGTCGTGCTCGCGACCGCCACGCCACCAGGGTCGGTGACAACCCCCCATCCCAA
HCV5	GCAGAGACGGCTGGAGCTAGGCTCGTCGTCTTGGCCACGGCCACTCCCCCCGGCAGTGTGACAACGCCCCACCCCAA
HCV6	GCGGAGACTGCCGGAGTGCGCCTTACTGTGCTCGCAACAGCAACGCCGCCTGGTTCTGTCACTGTCCCCCCATCCTAA
	4083 4090 4100 4110 4120 4130 4140 4150 4159
H77 (NC_004102)	CATCGAGGAGGTTGCTCTGTCCACCACCGGAGAGATCCCTTTTTACGGCAAGGCTATCCCCCTCGAGGTGATCAAGG
HCV1	CATCGAGGAGGTTGCTCTGTCCACCACCGGAGAGATCCCCTTTTACGGCAAGGCTATCCCCCTCGAGGCAATCAAGG
HCV2	CATAGAGGAGGTGGCCCTCGGTCATGAGGGCGAGATCCCCTTTTATGGCAAGGCTATTCCCCTAGCTTACATCAAGG
HCV3	CATCGAAGAAGTGGCCCTGGGCTCTGAAGGTGAGATCCCTTTCTACGGTAAGGCTATACCGATAGCCCTGCTCAAGG
HCV4	CATAGAGGAGGTCGCCCTGCCGACGACGGGAGAGATACCCTTCTACGGCAGGGCGATCCCCCTAGCTGTCATCAAGG
HCV5	CATCGAGGAGGTGGCCCTGCCTTCGGAGGGGGAAATCCCCTTCTACGGCAGGGCCATCCCCCTTGCTCTTATAAAGG
HCV6	CATAACAGAGACAGCCCTCCCTACTACGGGAGAAATACCCTTTTATGGGAAGGCAATCCCCCTTGAGTACATCAAAG

Table S5. *Cont.*

	4160 4170 4180 4190 4200 4210 4220 4230 4236
H77 (NC_004102)	GGGGAAGACATCTCATCTTCTGCCACTCAAAGAAGAAGTGCGACGAGCTCGCCGCGAAGCTGGTCGCATTGGGCATC
HCV1	GGGGGAGACATCTCATCTTCTGCCACTCAAAGAAGAAGTGTGACGAGCTCGCCGCAAAGCTGGTCGCACTGGGCATC
HCV2	GGGGCAGACACCTGATCTTTTGCCATTCAAAGAAGAAGTGCGACGAGCTCGCAGCAGCCCTTCGGGGCATGGGTGTC
HCV3	GGGGGAGGCACCTTATCTTTTGCCATTCCAAGAAAAAATGTGATGAGATAGCATCCAAACTCAGAGGCATGGGGCTC
HCV4	GGGGGAGACACCTCATCTTCTGTCATTCAAAGAAGAAGTGTGACGAGCTGGCCAAGCAGCTGTCATCTCTTGGCCTC
HCV5	GCGGTAGGCATCTCATCTTCTGCCACTCTAAGAAAAAATGTGATGAACTTGCCAAGCAATTGACCAGCCAGGGCGTG
HCV6	GGGGAAGACATCTCATATTTTGCCACTCAAAGAAGAAGTGCGACGAGCTGGCCAAGCAACTGACATCACTTGGCTTA
	4237 4240 4250 4260 4270 4280 4290 4300 4313
H77 (NC_004102)	AATGCCGTGGCCTACTACCGCGGTCTTGACGTGTCTGTCATCCCGACCAGCGGCGATGTTGTCGTCGTGTCGACCGA
HCV1	AATGCCGTGGCCTACTACCGCGGTCTTGACGTGTCCGTCATCCCGACCAGCGGCGATGTTGTCGTCGTGGCAACTGA
HCV2	AATGCCGTTGCCTACTACAGGGGTCTCGACGTCTCCGTTATACCAACTCAAGGAGACGTGGTGGTCGTCGCCACCGA
HCV3	AACGCTGTAGCGTACTATAGGGGTCTTGATGTGTCCGTCATACCAACAACAGGAGACGTCGTAGTTTGCGCTACTGA
HCV4	AATGCTGTGGCCTATTACAGGGGGCTAGACGTCTCGGTGATACCAACATCTGGGGACGTCGTGGTATGCGCCACAGA
HCV5	AACGCCGTGGCATATTACAGAGGTCTAGACGTCGCCGTCATACCCGCGACAGGAGACGTGGTCGTGTGCAGCACTGA
HCV6	AACGCCGTCGCATTCTACAGAGGTGTCGATGTGTCCGTCATCCCCACCTCGGGCGATGTGGTCGTCTGCGCCACAGA
	4314 4320 4330 4340 4350 4360 4370 4380 4390
H77 (NC_004102)	TGCTCTCATGACTGGCTTTACCGGCGACTTCGACTCTGTGATAGACTGCAACACGTGTGTCACTCAGACAGTCGATT
HCV1	TGCTCTCATGACCGGCTTTACCGGCGACTTCGACTCGGTGATAGACTGCAACACGTGTGTCACCCAGACAGTCGATT
HCV2	TGCCCTAATGACTGGATACACTGGTGACTTTGACTCTGTCATCGACTGCAACGTTGCAGTCACTCAGATTGTTGACT
HCV3	CGCCCTCATGACTGGATTCACCGGAGACTTCGATTCTGTCATAGATTGCAACGTGGCTGTTGAACAGTACGTTGACT
HCV4	CGCCCTCATGACAGGCTTCACCGGCGACTTTGACTCAGTGATAGACTGCAACACGTCTGTCATACAGACTGTTGACT
HCV5	CGCGCTCATGACGGGGTTTACCGGCGACTTTGATTCTGTCATAGACTGCAACTCCGCCGTCACGCAGACGGTGGACT
HCV6	CGCCCTTATGACTGGCTACACAGGCGATTTCGACTCCGTCATCGACTGTAACGTTGCCGTGACACAGGTGGTTGACT

Table S5. *Cont.*

	4391 4400 4410 4420 4430 4440 4450 4460 4467
H77 (NC_004102)	TCAGCCTTGACCCTACCTTTACCATTGAGACAACCACGCTCCCCCAGGATGCTGTCTCCAGGACTCAACGCCGGGGC
HCV1	TCAGCCTTGACCCTACCTT**C**ACCATTGAGACAACCACGCT**T**CCCCAGGATGCTGTCTCC**C**G**C**ACTCAACG**T**CGGGGC
HCV2	TCAGCCT**A**GACCC**A**ACCTTTACCAT**CACC**AC**TCAA**AC**CG**TCCC**T**CAGGA**C**GCTGTCTCC**C**G**T**A**G**TCAACG**TA**G**A**GG**G**
HCV3	TCAGCCT**G**GACCC**C**ACCTTT**T**CCATTGAGAC**TCG**CAC**TGCT**CCCCA**A**GA**C**GC**G**GT**T**TCC**C**G**C**A**GC**CAACG**T**C**GT**GGC
HCV4	TCAGCCT**G**GACCCTACCTT**CT**CCAT**A**GAGAC**C**AC**A**AC**TG**T**A**CCCCAGGA**C**GC**G**GT**G**TCC**C**GGA**GC**CA**G**CG**GA**G**A**GGC
HCV5	TCAG**T**CT**G**GA**T**CC**C**ACCTT**C**ACCATTGAGAC**T**ACCAC**AG**T**G**CCCCAGGA**C**GC**A**GT**G**TCCAG**A**A**GC**CA**G**CG**TA**GGGGC
HCV6	TCAGCCT**G**GACCC**A**AC**A**TTT**T**CCAT**A**GAGAC**T**ACCAC**TG**TCCCCCAGGATGCTGTCTCC**C**GGA**GC**CAACG**G**CG**A**GGC
	4468 4480 4490 4500 4510 4520 4530 4540 4544
H77 (NC_004102)	AGGACTGGCAGGGGGAAGCCAGGCATCTACAGATTTGTGGCACCGGGGGAGCGCCCCTCCGGCATGTTCGACTCGTC
HCV1	AGGACTGGCAGGGGGAAGCCAGGCATCTACAGATTTGTGGCACCGGGGGAGCGCCCCTCCGGCATGTTCGACTCGTC
HCV2	AG**A**ACTGG**G**AGGGGG**CGA**C**TG**GGCAT**T**TACAG**G**T**A**TGT**CT**C**GT**C**A**GG**C**GAG**A**G**G**CC**G**TC**T**GG**G**ATGTTCGAC**AGCGT**
HCV3	**C**G**T**AC**G**GGC**C**G**A**GG**T**A**GA**C**TC**GG**T**A**CG**TAC**C**GAT**A**TGT**CA**C**C**CC**C**GG**T**GA**AA**G**A**CC**G**TC**T**GG**A**ATGTT**T**GACTCG**GT**
HCV4	**C**G**C**ACTGG**T**AGGGGGA**G**G**TT**AGGCAT**A**TAC**C**G**G**T**A**TGT**CA**C**C**CCGGGGGAG**A**G**A**CC**G**TCCGGCATGTT**T**GAC**A**C**C**TC
HCV5	**C**G**C**AC**G**GG**A**AG**A**GG**T**A**G**GC**AC**GGCAT**A**TAC**C**G**G**T**AC**GT**CT**C**GT**C**T**GGGGAG**A**G**A**CC**G**TC**T**GGCAT**T**TTCGACTC**CGT**
HCV6	AG**C**AC**G**GG**G**AGGGG**T**AAGCC**C**GG**GG**T**G**TACAGAT**A**TGT**CT**C**G**C**AA**GGGGAGCG**G**CC**G**TC**G**GG**T**ATGTTCGAC**A**C**CGT**
	4545 4550 4560 4570 4580 4590 4600 4610 4621
H77 (NC_004102)	CGTCCTCTGTGAGTGCTATGACGCGGGCTGTGCTTGGTATGAGCTCACGCCCGCCGAGACTACAGTTAGGCTACGAG
HCV1	CGTCCTCTGTGAGTGCTATGACGCGGGCTGTGCTTGGTATGAGCTCACGCCCGCCGAGAC**C**ACAGTTAGGCTACGAG
HCV2	**A**GT**G**CTCTGTGAGTGCTA**C**GA**T**GC**C**GG**GGCA**GC**C**TGGTA**C**GAGCTCACGCC**T**GC**T**GAGACTAC**G**GT**G**AG**A**CT**C**CG**G**G
HCV3	**T**GT**T**CTCTGTGAGTGCTATGACGCGGGCTG**CT**C**G**TGGTA**C**GA**T**CT**GCA**GCCCGC**T**GAGAC**C**ACAGT**C**AG**A**CT**GA**GAG
HCV4	**A**GT**G**CTCTGTGAGTGCTATGA**T**GC**T**GG**G**TG**C**GCTTGGTA**C**GA**A**CT**G**AC**A**CC**G**GC**T**GAGAC**A**ACA**ACC**AGGCT**GA**G**G**G
HCV5	**G**GT**G**CTCTGTGAGTGCTA**C**GA**T**GC**C**GG**A**TGTGC**A**TGGTA**C**GA**T**CT**G**AC**C**CC**T**GC**T**GAGACTAC**C**GT**G**AGG**T**T**GC**G**T**G
HCV6	CGTCCT**G**TG**C**GAG**GC**CTATGAC**A**CGGG**A**TG**C**GC**G**TGGTA**C**GA**A**CT**A**AC**C**CC**TT**C**T**GA**A**AC**A**AC**T**GT**C**AGGCT**GC**G**G**G

Table S5. *Cont.*

	4622 4630 4640 4650 4660 4670 4680 4690 4697
H77 (NC_004102)	CGTACATGAACACCCCGGGGCTTCCCGTGTGCCAGGACCATCTTGAATTTTGGGAGGGCGTCTTTACGGGCCTCAC
HCV1	CGTACATGAACACCCCGGGGCTTCCCGTGTGCCAGGACCATCTTGAATTTTGGGAGGGCGTCTTCACGGGCCTCAC
HCV2	CTTATTTCAACACGCCCGGTTTGCCCGTGTGTCAAGACCACCTGGAGTTCTGGGAGGCGGTCTTCACAGGTCTCAC
HCV3	CTTACTTGTCCACGCCGGGGTTACCTGTCTGCCAAGACCATCTAGACTTTTGGGAGAGCGTCTTCACTGGACTAAC
HCV4	CTTATTTCAACACACCAGGCCTCCCCGTCTGCCAAGACCATCTTGAGTTCTGGGAGAGCGTCTTTACGGGGTTAAC
HCV5	CTTACCTGAACACCCCCGGGCTCCCTGTCTGTCAAGACCATCTCGAATTTTGGGAGGGGGTGTTCACAGGGCTTAC
HCV6	CCTACCTGAACACTCCTGGCCTCCCCGTATGCCAAGACCACCTTGAGTTTTGGGAGGGCGTGTTTACTGGCCTGAC
	4698 4710 4720 4730 4740 4750 4760 4773
H77 (NC_004102)	TCATATAGATGCCCACTTTCTATCCCAGACAAAGCAGAGTGGGGAGAACTTTCCTTACCTGGTAGCGTACCAAGCC
HCV1	CCATATAGATGCCCACTTCCTATCCCAGACAAAGCAGAGTGGGGAGAACTTTCCTTACCTGGTAGCGTACCAAGCC
HCV2	ACACATTGATGCCCACTTCCTCTCCCAGACGAAGCAAGGAGGAGACAACTTTGCGTACCTAACGGCCTACCAGGCC
HCV3	TCACATAGATGCCCACTTTCTGTCACAGACTAAGCAGCAGGGACTCAACTTCTCGTACCTAACTGCCTACCAAGCC
HCV4	CCATATAGACGCCCACTTCCTATCCCAGACCAAGCAGGCGGGTGACAACTACCCCTACCTGGTTGCTTACCAGGCC
HCV5	TAACATCGACGCCCACATGCTGTCACAGACCAAACAGGGTGGGGAAAACTTCCCATACCTTGTGGCGTACCAAGCA
HCV6	CCACATAGATGCCCACTTCCTCTCTCAGACGAAGCAGGCGGGGGAGAACTTCGCGTACCTCGTGGCCTATCAGGCT
	4774 4780 4790 4800 4810 4820 4830 4840 4849
H77 (NC_004102)	ACCGTGTGCGCTAGGGCTCAAGCCCCTCCCCCATCGTGGGACCAGATGTGGAAGTGTTTGATCCGCCTTAAACCCA
HCV1	ACCGTGTGCGCTAGGGCTCAAGCCCCTCCCCCATCGTGGGACCAGATGTGGAAGTGTTTGATCCGCCTCAAGCCCA
HCV2	ACAGTGTGCGCTAGGGCAAAGGCCCCTCCTCCTTCGTGGGACGTGATGTGGAAGTGTCTAACTAGGCTCAAACCTA
HCV3	ACTGTGTGCGCCCGCGCGCAGGCTCCTCCCCCAAGTTGGGACGAGATGTGGAAGTGTCTCGTGCGGCTTAAGCCAA
HCV4	ACAGTCTGTGCCAGGGCGCTGGCCCCCCCACCCAGCTGGGACACTATGTGGAAGTGCCTGATCCGCCTCAAGCCCA
HCV5	ACAGTCTGTGTTCGTGCGAAAGCGCCCCCCCCCAGCTGGGACACAATGTGGAAGTGCATGCTCCGTCTCAAACCGA
HCV6	ACAGTGTGCGCCAGGGCGAAAGCCCCCCCCCCCTCTTGGGATACGATGTGGAAGTGTCTCATCCGGCTCAAGCCCA

Table S5. *Cont.*

	4850 4860 4870 4880 4890 4900 4910 4920 4925
H77 (NC_004102)	CCCTCCATGGGCCAACACCCCTGCTATACAGACTGGGCGCTGTTCAGAATGAAGTCACCCTGACGCACCCAATCAC
HCV1	CCCTCCATGGGCCAACACCCCTGCTATACAGACTGGGCGCTGTTCAGAATGAAGTCACCCTGACGCACCCAATCAC
HCV2	CACTGACTGGTCCTACCCCCCTCCTGTACCGCTTGGGCGCCGTGACCAACGAGGTCACCCTGACGCACCCCGTGAC
HCV3	CACTACATGGACCTACGCCCCTTCTATATCGGTTGGGGCCTGTCCAAAATGAAATCTGCTTGACACACCCCATCAC
HCV4	CCCTGCACGGACCCACCCCCCTCCTTTACAGGCTGGGGGCTGTGCAAAATGAGGTGACACTCACCCATCCTATAAC
HCV5	CTCTAACTGGCCCCACTCCCCTCCTGTACAGGCTGGGGGCCGTCCAGAATGAGATCACACTGACGCACCCCATCAC
HCV6	CCCTTACAGGGCCCACTCCCCTGTTGTATAGGCTGGGGGCGGTCCAAAATGAGATCATAACAACCCATCCAATCAC
	4926 4930 4940 4950 4960 4970 4980 4990 5001
H77 (NC_004102)	CAAATACATCATGACATGCATGTCGGCCGACCTGGAGGTCGTCACGAGCACCTGGGTGCTCGTTGGCGGCGTCCTG
HCV1	CAAATACATCATGACATGCATGTCGGCTGACCTGGAGGTCGTCACGAGCACCTGGGTGCTCGTTGGCGGCGTCCTG
HCV2	GAAATACATCGCCACGTGCATGCAAGCTGACCTTGAGATCATGACAAGCACATGGGTCCTGGCAGGGGGGGTGCTG
HCV3	AAAATACATCATGGCATGCATGTCAGCTGATCTGGAAGTAACCACCAGCACCTGGGTGTTGCTTGGAGGGGTCCTC
HCV4	CAAATACATCATGGCCTGCATGTCAGCGGACCTTGAGGTGGTGACCAGCACGTGGGTCTTGGTGGGCGGCATCCTG
HCV5	CAAGTACATTATGGCTTGCATGTCCGCGGACTTGGAGGTCATTACCAGCACTTGGGTTTTGGTAGGGGGTGTCGTG
HCV6	CAAATACATCATGACCTGCATGTCAGCGGACTTGGAGGTCATCACCAGCACATGGGTCCTCGTGGGTGGAGTCCTA
	5002 5010 5020 5030 5040 5050 5060 5070 5077
H77 (NC_004102)	GCTGCTCTGGCCGCGTATTGCCTGTCAACAGGCTGCGTGGTCATAGTGGGCAGGATTGTCTTGTCCGGGAAGCCGG
HCV1	GCTGCTTTGGCCGCGTATTGCCTGTCAACAGGCTGCGTGGTCATAGTGGGCAGGATTGTCTTGTCCGGGAAGCCGG
HCV2	GCCGCCGTGGCAGCTTACTGCCTGGCGACCGGCTGCGTTTCCATCATTGGCCGCATACACCTGAATGATCGGGTAG
HCV3	GCGGCCCTAGCGGCCTACTGCTTGTCAGTCGGCTGCGTTGTGATTGTGGGTCATATTGAGCTGGGGGGCAAGCCGG
HCV4	GCAGCCCTAGCTGCATACTGCTTGTCAGTGGGCAGCGTGGTGATCGTCGGGAGGGTCGTCCTATCGGGCCAACCTG
HCV5	GCGGCCCTAGCGGCCTACTGCTTGACGGTGGGCTCGGTGGCCATAGTCGGTAGAATCATCCTATCTGGGAGACCTG
HCV6	GCCGCGCTCGCAGCCTACTGCTTGTCAGTGGGCTGCGTTGTCATCTGCGGCAGGATCACTACGACTGGCAAGCCTG

Table S5. *Cont.*

	5078 5090 5100 5110 5120 5130 5140 5153
H77 (NC_004102)	CAATTATACCTGACAGGGAGGTTCTCTACCAGGAGTTCGATGAGATGGAAGAGTGCTCTCAGCACTTACCGTACAT
HCV1	CAATCATACCTGACAGGGAAGTCCTCTACCGGGAGTTCGATGAGATGGAAGAGTGCTCTCAGCACCTACCGTACAT
HCV2	TTGTGGCCCCTGACAAGGAAGTCCTATATGAGGCCTTTGATGAGATGGAAGAATGCGCCTCCAAAGCCGCCCTCAT
HCV3	CACTCGTTCCAGACAAAGAGGTGTTGTATCAACAATACGATGAGATGGAGGAGTGCTCGCAAGCCGCCCCATATAT
HCV4	CCGTCATCCCCGACCGAGAGGTGCTCTACCAACAGTTTGACGAGATGGAGGAGTGCTCCAAGCACCTTCCACTAGT
HCV5	CCATCATTCCCGACAGGGAGGTGCTATACCAGCAATTTGATGAGATGGAGGAGTGCTCGGCCTCCCTGCCCTATAT
HCV6	CTGTTGTCCCTGATCGGGAGGTCTTGTACCAACAATTTGACGAGATGGAGGAGTGCTCTAGGCACATCCCCTACCT
	5154 5160 5170 5180 5190 5200 5210 5220 5229
H77 (NC_004102)	CGAGCAAGGGATGATGCTCGCTGAGCAGTTCAAGCAGAAGGCCCTCGGCCTCCTGCAGACCGCGTCCCGCCAAGCA
HCV1	CGAGCAAGGGATGATGCTCGCCGAGCAGTTCAAGCAGAAGGCCCTCGGCCTCCTGCAGACCGCGTCCCGCCAGGCA
HCV2	TGAGGAAGGGCAGCGGATGGCGGAGATGCTCAAGTCCAAGATACAAGGCCTCCTACAACAGGCCACAAAACAGGCC
HCV3	CGAACAAGCTCAGGTAATAGCCCACCAGTTCAAGGAGAAAGTCCTTGGATTGCTGCAGCGAGCCACCCAACAACAA
HCV4	CGAGCACGGCCTGCAACTGGCAGAGCAATTCAAACAGAAGGCCCTCGGCGTGCTGAACATCGCCGGCAAGCAGGCC
HCV5	GGACGAGGCACGTGCCATTGCCGGGCAATTCAAGGAGAAAGTGCTCGGCCTCATTGGCACGGCCGGCCAGAAAGCT
HCV6	CGCTGAGGGCCAGCAGATAGCCGAACAGTTCAAACAAAAGGTGCTCGGTCTCATCCAGACCACCGCCAAGCAGGCA
	5230 5240 5250 5260 5270 5280 5290 5300 5305
H77 (NC_004102)	GAGGTTATCACCCCTGCTGTCCAGACCAACTGGCAGAAACTCGAGGTCTTCTGGGCGAAGCACATGTGGAATTTCA
HCV1	GAGGTTATCGCCCCTGCTGTCCAGACCAACTGGCAAAAACTCGAGGCCTTCTGGGCGAAGCATATGTGGAACTTCA
HCV2	CAAGACATACAGCCAGCCATACAGTCATCATGGCCCAAGCTTGAACAATTTTGGGCCAAACACATGTGGAACTTCA
HCV3	GCTGTCATTGAGCCCATAGTAGCTACCAACTGGCAAAAGCTTGAGGCCTTCTGGCACAAGCATATGTGGAATTTTG
HCV4	CAGGAGGCAGCACCAGTGGTCCAGTCCAACTTTGCCAAGCTTGAACAGTTCTGGGCAAAGCACATGTGGAACTTCA
HCV5	GAAACTCTGAAACCGGCAGCCACGTCTATGTGGAGCAGGGCCGAGCAGTTCTGGGCAAAACACATGTGGAACTTTG
HCV6	GAAGAACTGAAGCCTGCTGTCCATTCCGCGTGGCCTAAGCTTGAGCAGTTTTGGCAGAAACACATGTGGAACTTTG

Table S5. *Cont.*

	5306 5310 5320 5330 5340 5350 5360 5370 5381
H77 (NC_004102)	TCAGTGGGATACAATACTTGGCGGGCCTGTCAACGCTGCCTGGTAACCCCGCCATTGCTTCATTGATGGCTTTTAC
HCV1	TCAGTGGGATACAATACTTGGCGGGCCTGTCAACGCTGCCTGGTAACCCCGCCATTGCATCATTGATGGCTTTTAC
HCV2	TCAGTGGCATACAGTACCTGGCGGGACTCTCCACCCTACCGGGAAATCCCGCAGTGGCATCAATGATGGCTTTCAG
HCV3	TGAGTGGGATCCAGTACCTAGCAGGCCTCTCCACTTTGCCTGGCAACCCTGCTGTGGCGTCTCTTATGGCGTTCAC
HCV4	TCAGCGGCATCCAATACCTTGCCGGGTTGTCCACCTTGCCCGGCAACCCTGCCATTGCTTCCCTCATGTCCTTCAC
HCV5	TCAGTGGGATCCAATACCTAGCTGGCCTTTCGACCTTGCCGGGCAATCCCGCCGTGGCCACGTTGATGTCTTTCAC
HCV6	TCAGCGGGATTCAGTACCTGGCGGGCTTATCCACCCTGCCCGGCAACCCAGCTGTGGCATCCCTCATGTCATTCTC
	5382 5390 5400 5410 5420 5430 5440 5450 5457
H77 (NC_004102)	AGCTGCCGTCACCAGCCCACTAACCACTGGCCAAACCCTCCTCTTCAACATATTGGGGGGGTGGGTGGCTGCCCAG
HCV1	AGCTGCTGTCACCAGCCCACTAACCACTAGCCAAACCCTCCTCTTCAACATCTTGGGGGGGTGGGTGGCTGCCCAG
HCV2	CGCCGCACTGACTAGCCCACTATCCACCAGCACCACCATCCTCTTGAACATCATGGGGGGATGGTTGGCCTCCCAG
HCV3	TGCTTCAGTCACCAGTCCCCTGACGACCAACCAAACTATGTTTTTTAACATACTCGGGGGGTGGGTTGCTACCCAT
HCV4	CGCCGCTGTCACCAGCCCCCTGACCACCCAACAAACCCTCCTCTTCAACATCTTAGGAGGGTGGGTGGCCTCGCAG
HCV5	CGCCGCGGTGACCTCCCCCCTTACAACACAACAGACCCTCCTCTTTAATATTCTAGGAGGGTGGGTGGCCTCACAG
HCV6	AGCGTCGCTGACCAGCCCACTGACCACTTCTCAGACCCTCCTCCTCAACATCTTAGGGGGCTGGGTAGCCTCCCAA
	5458 5470 5480 5490 5500 5510 5520 5533
H77 (NC_004102)	CTCGCCGCCCCCGGTGCCGCTACCGCCTTTGTGGGCGCTGGCTTAGCTGGCGCCGCCATCGGCAGCGTTGGACTGG
HCV1	CTCGCCGCCCCCGGTGCCGCTACCGCCTTTGTGGGCGCTGGCTTAGCTGGCGCCGCCATCGGCAGTGTTGGACTGG
HCV2	ATTGCCCCCCCTGCCGGGGCCACTGGCTTCGTTGTCAGTGGTCTAGTGGGGGCGGCCGTCGGAAGCATAGGCCTGG
HCV3	TTGGCAGGGCCCCAGAGCTCTTCCGCATTCGTGGTAAGCGGCTTGGCCGGCGCTGCCATAGGGGGTATAGGCCTGG
HCV4	ATCGCGACCCCCACGGCCTCTACGGCTTTTGTCGTGAGCGGCCTCGCGGGGGCTGCGGTTGGCAGTGTGGGCCTGG
HCV5	ATCGCGCCACCCACGGCCGCCACTGCGTTCGTCGTTAGTGGAATGGCCGGGGCCGCAGTCGGTAGCATCGGGTTGG
HCV6	CTGGCTACCCCCCCCGCGTCTACAGCTTTTGTGGTGAGCGGCCTAGCAGGCGCCGCGGTGGGAAGCATTGGGCTCG

Table S5. *Cont.*

	5534 5540 5550 5560 5570 5580 5590 5600 5609
H77 (NC_004102)	GGAAGGTCCTCGTGGACATTCTTGCAGGGTATGGCGCGGGCGTGGCGGGAGCTCTTGTAGCATTCAAGATCATGAG
HCV1	GGAAGGTCCTCGTGGACATCCTTGCAGGGTATGGCGCGGGCGTGGCGGGAGCTCTCGTAGCATTCAAGATCATGAG
HCV2	GTAAGATACTGGTGGACGTTTTGGCCGGGTACGGTGCAGGCATTTCAGGGGCCCTCGTAGCTTTTAAGATCATGAG
HCV3	GCAGGGTCTTGCTTGACATCCTGGCAGGATACGGAGCTGGTGTCTCAGGCGCCTTGGTGGCTTTTAAGATCATGGG
HCV4	GGAAGATCCTGGTCGACATTCTCGCTGGCTACGGCGCCGGCGTAGCCGGCGCTGTGGTCACCTTCAAGATCATGAG
HCV5	GTAGGGTCCTCATCGACATCCTAGCTGGATATGGAGCAGGGGTGGCTGGAGCACTCGTCGCCTTCAAGATCATGTG
HCV6	GGAAGGTCCTGGTTGACATCCTCGCCGGATACGGAGCGGGCGTGTCGGGGGCTCTCGTCGCTTTCAAGATCATGAG
	5610 5620 5630 5640 5650 5660 5670 5680 5685
H77 (NC_004102)	CGGTGAGGTCCCCTCCACGGAGGACCTGGTCAATCTGCTGCCCGCCATCCTCTCGCCTGGAGCCCTTGTAGTCGGT
HCV1	CGGCGAGGTCCCCTCCACGGAGGACCTGGTCAATCTACTGCCCGCCATCCTCTCGCCTGGAGCCCTTGTAGTCGGT
HCV2	CGGCGAGAAGCCCTCGGTAGAAGATGTTGTCAATCTCCTGCCTGCTATCCTGTCTCCTGGCGCTTTGGTAGTGGGA
HCV3	AGGAGAACTCCCCACTGCTGAGGACATGGTCAACCTGTTGCCCGCCATACTATCTCCGGGCGCTCTCGTCGTCGGT
HCV4	CGGCGAGATGCCATCAACAGAGGACTTGGTAAACTTGCTCCCGGCCATACTATCGCCGGGAGCCTTGGTGGTGGGG
HCV5	CGGGGAGAAGCCGACAGCCGAAGACCTAGTAAACCTCCTGCCTTCAATACTCTGCCCTGGAGCCCTAGTGGTCGGG
HCV6	CGGCGAGACCCCGACAACGGAGGACATGGTCAACCTGCTACCCGCCCTGTTGTCACCAGGGGCTCTCGTGGTGGGC
	5686 5690 5700 5710 5720 5730 5740 5750 5761
H77 (NC_004102)	GTGGTCTGCGCAGCAATACTGCGCCGGCACGTTGGCCCGGGCGAGGGGGCAGTGCAATGGATGAACCGGCTAATAG
HCV1	GTGGTCTGCGCAGCAATACTGCGCCGGCACGTTGGCCCGGGCGAGGGGGCAGTGCAGTGGATGAACCGGCTGATAG
HCV2	GTCATCTGTGCGGCAATCCTGCGCCGCCACGTCGGCCAGGGAGAGGGGGCGGTCCAGTGGATGAACAGGCTGATCG
HCV3	GTGATATGCGCTGCCATACTACGTCGACACGTAGGACCTGGGGAGGGGGCGGTGCAGTGGATGAACAGGCTCATCG
HCV4	GTGGTGTGCGCCGCGATCCTGCGGCGTCACGTGGGCCCGGGCGAGGGGGCTGTGCAGTGGATGAACCGTCTCATTG
HCV5	GTCATATGCGCCGCGGTTCTGCGGCGCCATATTGGCCCAGGCGAGGGGGCTGTGCAGTGGATGAATAGGCTGATTG
HCV6	GTCGTGTGTGCTGCCATCTTGCGACGCCACGTTGGCCCCGCTGAGGGGGCTACCCAGTGGATGAACAGGCTAATAG

Table S5. *Cont.*

	5762 5770 5780 5790 5800 5810 5820 5830 5837
H77 (NC_004102)	CCTTCGCCTCCCGGGGGAACCATGTTTCCCCCACGCACTACGTGCCGGAGAGCGATGCAGCCGCCCGCGTCACTGC
HCV1	CCTTCGCCTCCCGGGGGAACCATGTTTCCCCCACGCACTACGTGCCGGAGAGCGATGCAGCTGCCCGCGTCACTGC
HCV2	CCTTTGCCTCCAGAGGAAACCATGTTGCCCCTACCCACTACGTGGCGGAGTCTGACGCCTCGCAGCGCGTGACGCA
HCV3	CATTCGCATCCCGGGGCAACCACGTCTCACCAACGCACTATGTTCCCGAGAGCGATGCTGCAGCGAGGGTCACCGC
HCV4	CGTTCGCATCGCGAGGCAATCACGTGTCTCCCACGCACTACGTCCCCGAGACCGACGCAGCGGCCCGCGTGACTGC
HCV5	CGTTCGCTTCCCGGGGCAATCATGTGTCTCCGACACACTACGTGCCCGAGACGGACGCGTCGGCCAAAGTCACGCA
HCV6	CCTTTGCCTCAAGGGGCAACCACGTGTCCCCCACGCACTACGTGCCTGAGACTGACGCGTCACGGAAAGTGACTAA
	5838 5850 5860 5870 5880 5890 5900 5913
H77 (NC_004102)	CATACTCAGCAGCCTCACTGTAACCCAGCTCCTGAGGCGACTGCATCAGTGGATAAGCTCGGAGTGTACCACTCCA
HCV1	CATACTCAGCAGCCTCACTGTCACCCAGCTCCTGAGGCGACTGCACCAGTGGATAAGCTCGGAGTGCACCACTCCA
HCV2	GGTGCTAAGCTCACTCACAATTACCAGCTTACTTAGGAGACTACATACCTGGATCACTGAAGATTGCCCAGTCCCA
HCV3	ATTGCTGAGTTCTCTAACTGTCACAAGTCTGCTCCGGCGGTTGCACCAGTGGATCAATGAAGACTACCCAAGTCCT
HCV4	CATACTATCATCCCTGACTGTGACATCCCTTCTCAGACGCCTCCACAAGTGGATCAATGAGGACTGCTCCACTCCC
HCV5	GCTGCTCAGCTCCCTCACAGTAACATCTCTTCTGAAGAGGCTCCATACATGGATCGGTGAGGACTACTCCACGCCC
HCV6	CATACTCAGTTCCCTTACCATCACCAGCCTACTCCGTAAGTTACATCAGTGGATCAATGAAGACTGGTCCACCCCT
	5914 5920 5930 5940 5950 5960 5970 5980 5989
H77 (NC_004102)	TGCTCCGGTTCCTGGCTAAGGGACATCTGGGACTGGATATGCGAGGTGCTGAGCGACTTTAAGACCTGGCTGAAAG
HCV1	TGCTCCGGTTCCTGGCTAAGGGACATCTGGGACTGGATATGCGAGGTGCTGAGCGACTTTAAGACCTGGCTAAAGG
HCV2	TGCTCGGGGTCTTGGCTCCGGGACATTTGGGATTGGGTTTGTTCCATCCTCACAGACTTTAAGAACTGGCTGTCTT
HCV3	TGCAGCGGCGATTGGCTGCGTACCATCTGGGACTGGGTTTGCACGGTGTTGTCCGACTTCAAGACATGGCTCTCTG
HCV4	TGTGACTCATCTTGGCTGTGGGAGATCTGGGACTGGGTCTGCACCGTACTGAGTGACTTCAAGACGTGGCTCAAGG
HCV5	TGTGATGGCACATGGCTTAGGGCCATTTGGGACTGGGTCTGCACGGCACTGACAGACTTCAAAGCCTGGCTGCAGG
HCV6	TGCTCTACCTCATGGTTGCGCGACATCTGGGACTGGGTGTGTACTGTGCTATCTGACTTCAAAACCTGGCTCAAAG

Table S5. *Cont.*

	5990 6000 6010 6020 6030 6040 6050 6060 6065
H77 (NC_004102)	CCAAGCTCATGCCACAACTGCCTGGGATTCCCTTTGTGTCCTGCCAGCGCGGGTATAGGGGGGTCTGGCGAGGAGA
HCV1	CCAAGCTCATGCCACAACTGCCTGGGATCCCCTTTGTGTCCTGCCAACGCGGGTATAGGGGGGTCTGGCGAGGGGA
HCV2	CAAAATTGCTCCCCAAAATGCCCGGCCTTCCCTTCATCTCTTGCCAAAAGGGATACAGGGGTGTGTGGGCTGGCAC
HCV3	CTAAGATCATGCCAGCGCTCCCTGGGCTGCCCTTCATTTCCTGTCAAAAGGGATACAAGGGCGTGTGGCGGGGGGA
HCV4	CTAAGCTACTGCCCCGCATGCCCGGCATCCCCTTCCTCTCCTGCCAAAAGGGGTACAAGGGGGAGTGGCGGGGGGA
HCV5	CAAAACTCCTCCCGCAACTCCCCGGAGTGCCCTTCCTCTCATGCCAGAGAGGGTACAAGGGTGTGTGGCGCGGGGA
HCV6	CCAAACTTGTCCCACCCCTGCCGGGGGTCCCCTTCCTCTCGTGCCAACGGGGATACAGGGGAGTCTGGAGAGGTGA
	6066 6070 6080 6090 6100 6110 6120 6130 6141
H77 (NC_004102)	CGGCATTATGCACACTCGCTGCCACTGTGGAGCTGAGATCACTGGACATGTCAAAAACGGGACGATGAGGATCGTC
HCV1	CGGCATCATGCACACTCGCTGCCACTGTGGAGCTGAGATCACTGGACATGTCAAAAACGGGACGATGAGGATCGTC
HCV2	GGGAATCATGACTACTCGGTGTCCATGCGGAGCAAACATCTCGGGCCATGTCCGCATGGGCACCATGAAAATAACA
HCV3	CGGTGTGATGTCAACACGCTGTCCTTGCGGGGCATCAATAACCGGTCATGTGAAGAATGGGTCCATGCGGCTTGCA
HCV4	CGGGGTAATGCACACCACATGCCCTTGCGGCGCAGAGCTGGCCGGCCACATCAAAAACGGCTCGATGAGGATCGTC
HCV5	TGGAGTGAATTCCACCAAGTGTCCGTGCGGAGCGACGATATCTGGCCACGTGAAGAACGGAACCATGAGAATCGTC
HCV6	CGGGGTGTGCCACACCACCTGCACCTGCGGGGCCGAGATAGCTGGCCACGTCAAAAACGGGACCATGAAGATCACA
	6142 6150 6160 6170 6180 6190 6200 6210 6217
H77 (NC_004102)	GGTCCTAGGACCTGCAGGAACATGTGGAGTGGGACGTTCCCCATTAACGCCTACACCACGGGCCCCTGTACTCCCC
HCV1	GGTCCTAAGACCTGCAGGAACATGTGGAGTGGGACCTTCCCCATCAACGCCTACACCACGGGCCCCTGCACCCCCC
HCV2	GGCCCGAAGACCTGCTTGAACATGTGGCAGGGGACCTTCCCCATCAATTGTTACACAGAAGGGCCTTGCGTGCCAA
HCV3	GGGCCGCGCACATGTGCTAACATGTGGCACGGTACTTTCCCCATCAATGAGTACACCACCGGACCCAGCACACCTT
HCV4	GGGCCCAAGACCTGCAGCAACACCTGGCATGGGACCTTCCCCATCAATGCTTACACCACAGGCCCCGGCGTGCCCA
HCV5	GGCCCGAAGATGTGCAGTAACACCTGGCACGGAACCTTCCCAATCAACGCCACAACAACAGGGCCCAGTGTACCTG
HCV6	GGGCCCCGGACATGCAGCAACATCTGGCACGGCACTTTCCCCATCAATGCCACCACGACCGGCCCTAGCGTGCCAA

Table S5. *Cont.*

	6218 6230 6240 6250 6260 6270 6280 6293
H77 (NC_004102)	TTCCTGCGCCGAACTATAAGTTCGCGCTGTGGAGGGTGTCTGCAGAGGAATACGTGGAGATAAGGCGGGTGGGGGA
HCV1	TTCCTGCGCCGAACTATACGTTCGCGCTGTGGAGGGTGTCTGCAGAGGAATACGTGGAGATAAGGCGGGTGGGGGA
HCV2	AACCCCCTCCTAATTACAAGACCGCAATTTGGAGGGTGGCAGCGTCGGAGTACGTTGAGGTCACGCAGCATGGCTC
HCV3	GCCCATCACCCAACTACACTCGCGCACTATGGCGCGTGGCTGCCAACAGCTACGTTGAGGTGCGCCGGGTGGGGGA
HCV4	TCCCAGCGCCGAACTACAAGTTCGCGCTGTGGAGGGTGTCCGCCGAGGAATACGTGGAGGTTCGCAGAGTGGGTGA
HCV5	CCCCGGCTCCCAACTACAAGTTCGCCTTGTGGAGGGTAGGCGCTGCGGACTACGCGGAGGTGCGCCGCGTAGGGGA
HCV6	TCCCAGCGCCCAACTACAAGAGGGCTCTTTGGAGGGTATCCGCTGAGGAGTACGTTGAGGTACGGAGGGTGGGCGA
	6294 6300 6310 6320 6330 6340 6350 6360 6369
H77 (NC_004102)	CTTCCACTACGTATCGGGTATGACTACTGACAATCTTAAATGCCCGTGCCAGATCCCATCGCCCGAATTTTTCACA
HCV1	CTTCCACTACGTGACGGGCATGACTACTGACAACCTTAAATGCCCGTGCCAGGTCCCATCGCCCGAATTTTTCACA
HCV2	TTTCTCGTACGTAACGGGGTTAACCAGTGACAACCTTAAGGTCCCTTGCCAGGTACCAGCTCCAGAGTTCTTCTCT
HCV3	CTTCCATTACATTACGGGGGCCACAGAAGATGAGCTCAAGTGTCCGTGCCAAGTGCCGGCTGCTGAGTTCTTTACT
HCV4	TTTCCATTATGTCACCGGGGTGACACAAGATAACATCAAGTGCCCCTGCCAGGTTCCTGCCCCTGAGTTCTTCACT
HCV5	CTACCACTACATTACGGGGGTGACACAGGACAATCTGAAGTGTCCCTGCCAGGTACCATCTCCAGAGTTCTTCACG
HCV6	CTGCCACTACGTGGTAGGGGTCACTGCTGACGACCTGAAATGCCCATGCCAGGTGCCTGCTCCTGAGTTCTTCACT
	6370 6380 6390 6400 6410 6420 6430 6440 6445
H77 (NC_004102)	GAATTGGACGGGGTGCGCCTACATAGGTTTGCGCCCCCTTGCAAGCCCTTGCTGCGGGAGGAGGTATCATTCAGAG
HCV1	GAATTGGACGGGGTGCGCCTGCATAGGTTCGCGCCCCCTTGCAAGCCCTTGCTGCGGGAGGAGGTCTCATTCAGAG
HCV2	TGGGTGGACGGGGTGCAAATACACCGATTCGCCCCCACTCCAGGTCCCTTCTTTCGGGATGAGGTAACGTTCACCG
HCV3	GAAGTGGATGGGGTGAGACTCCACCGTTACGCCCCTCCATGTAAGCCCCTGTTGAGGGATGAGATCACTTTCATGG
HCV4	GAGGTGGACGGCGTCAGGCTACACCGCCACGCCCCTGCGTGCAAGCCCCTGCTGAGGGACGAAGTGACCTTCACCG
HCV5	GAGCTGGACGGCGTGAGGATTCACCGCTTCGCGCCACCCTGCAACCCCCTCCTAAGGGAGGAGGTTACTTTCTCCG
HCV6	GAGGTTGATGGCGTGAGGATACACCGTTACGCGCCACCGTGCAAGCCCCTGCTGAGGGACGAAGTGACCTTCTCTG

Table S5. *Cont.*

	6446 6450 6460 6470 6480 6490 6500 6510 6521
H77 (NC_004102)	TAGGACTCCACGAGTACCCGGTGGGGTCGCAATTACCTTGCGAGCCCGAACCGGACGTAGCCGTGTTGACGTCCAT
HCV1	TAGGACTCCACGAGTACCCGGTGGGGTCGCAATTACCTTGCGAGCCCGAACCGGACGTGGCCGTGTTGACGTCCAT
HCV2	TAGGCCTCAATTCCTTTGTGGTCGGCTCTCAGCTCCCTTGTGACCCTGAGCCGGACACCGAGGTATTGGCCTCCAT
HCV3	TAGGGTTGAATTCCTACGCGATAGGATCTCAACTCCCCTGTGAGCCCGAACCAGATGTTTCTGTGCTGACCTCGAT
HCV4	TGGGCCTCAACACGTTTGTGGTGGGGTCCCAGCTCCCCTGCGAGCCAGAGCCAGACGTGGCCGTGTTAACATCCAT
HCV5	TGGGGCTGCACTCCTACGTGGTGGGGTCCCAACTACCCTGTGAGCCAGAACCTGATGTGACCGTCCTGACGTCAAT
HCV6	TGGGTCTCTCCACCTACGCCGTAGGGTCGCAGCTCCCTTGCGAGCCAGAGCCAGACGTGATGGTCGTCACATCAAT
	6522 6530 6540 6550 6560 6570 6580 6590 6597
H77 (NC_004102)	GCTCACTGATCCCTCCCATATAACAGCAGAGGCGGCCGGGAGAAGGTTGGCGAGAGGGTCACCCCCTTCTATGGCC
HCV1	GCTCACTGATCCCTCCCACATAACAGCAGAGGCGGCTAGGAGAAGGTTGGCGAGGGGGTCACCCCCCTCTTTGGCC
HCV2	GCTGACAGACCCGTCCCACATCACGGCGGAGGCGGCAGCCAGGCGATTGGCCAGGGGATCTCCCCCTTCACAGGCC
HCV3	GTTGAGAGACCCTTCCCATATCACCGCCGAGACGGCAGCGCGCCGCCTTGCGCGCGGGTCCCCTCCATCAGAGGCA
HCV4	GCTGACAGACCCATCCCACATCACCGCGGAGACGGCGCGCCGGAGGCTGGCCAGGGGGTCACCGCCCTCGTTGGCC
HCV5	GCTGTCAGACCCTGCCCACATCACGGCAGAGACGGCCAAGCGCAGGCTAGATCGAGGGTCTCCGCCCTCTCTGGCT
HCV6	GCTGACAGACCCCTCACACATCACCGCAGAGGCGGCAGCACGGCGCCTGGCGAGGGGGTCCCCCCCCTCCCTGGCC
	6598 6610 6620 6630 6640 6650 6660 6673
H77 (NC_004102)	AGCTCCTCGGCCAGCCAGCTGTCCGCTCCATCTCTCAAGGCAACTTGCACCGCCAACCATGACTCCCCTGACGCCG
HCV1	AGCTCCTCGGCTAGCCAGCTGTCTGCTCCATCTCTCAAGGCAACTTGCACCACCAACCATGACTCCCCTGACGCCG
HCV2	AGCTCCTCAGCGAGCCAGCTCTCCGCCCCGTCCTTGAAGGCTACCTGTACCACCCATAAGATGGCATATGATTGTG
HCV3	AGCTCATCCGCCAGCCAACTATCAGCTCCGTCGTTGAAGGCCACTTGCCAGACGCATAGGCCTCATCCAGACGCTG
HCV4	AGCTCCTCGGCTAGCCAGCTTTCCGCCCCATCCCTCAAAGCCACATGCACCGCCCACCACGACTCCCCTGGCGCTG
HCV5	AGCTCCTCGGCCAGTCAGCTCTCGGCCCCCTCCCTCAAGGCTACCTGCACTACACACCACCCAGACGCAGACCTCA
HCV6	AGCTCATCGGCCAGCCAGCTGTCAGCACCGTCACTGAAGGCCACATGCACAACGGACCACCCGGACGCGGAACTGA

Table S5. *Cont.*

	6674 6680 6690 6700 6710 6720 6730 6740 6749
H77 (NC_004102)	AGCTCATAGAGGCTAACCTCCTGTGGAGGCAGGAGATGGGCGGCAACATCACCAGGGTTGAGTCAGAGAACAAAGT
HCV1	AGCTCATAGAGGCCAACCTCCTGTGGAGGCAGGAGATGGGCGGCAACATCACCAGGGTTGAGTCAGAGAACAAAGT
HCV2	ACATGGTGGATGCCAACCTTTTC------------ATGGGAGGCGATGTGACCCGGATTGAGTCCGACTCCAAGGT
HCV3	AGCTAGTGGACGCCAACTTGTTATGG------GAGATGGGCAGCAACATCACACGGGTAGAGTCTGAAACAAAGGT
HCV4	ACCTCATCGAAGCCAATCTTCTGTGG------------GGTGCCAACGCCACCAGGGTTGAGTCAGAGAACAAGGT
HCV5	TAGAGGCTAATCTGCTATGGAGATGC---------ATGGGAGGCAACATCACGCGAGTGGAGGCCGAGAACAAGGT
HCV6	TCGAGGCCAACCTCCTGTGGAGG---------GAGATGGGGGGCAACATCACTCGAGTGGAGTCAGAGAACAAAGT
	6750 6760 6770 6780 6790 6800 6810 6820 6825
H77 (NC_004102)	GGTGATTCTGGACTCCTTCGATCCGCTTGTGGCAGAGGAGGATGAGCGGGAGGTCTCCGTACCCGCAGAAATTCTG
HCV1	GGTGATTCTGGACTCCTTCGATCCGCTTGTGGCGGAGGAGGATGAGCGGGAGATCTCCGTTCCCGCAGAAATCCTG
HCV2	GATCGTTCTCGACTCCCTCGACTCCATGACCGAGGTAGAGGACGATCGTGAGCCTTCTGTACCATCAGAGTACTTG
HCV3	TGTGATTCTTGATTCATTCGAACCTCTGAGAGCCGAAACTGATGACGCCGAGCTCTCGGTGGCTGCAGAGTGTTTC
HCV4	GGTGGTGCTGGACTCTTTTGAGCCACTTGTGGCCGAGCCGGATGACAGGGAGGTCTCCGTCGCCGCAGAGATCCTG
HCV5	CGTGATCCTTGACTCCTTTGAGCCGCTC---AAGGCGGAAGACGACAGGGAGATCTCTGTGTCCGCTGACTGCTTT
HCV6	CGTCGTACTCGACTCCTTTGAGCCCCTCACCCCTGAGTATGACGAGGAAATATCAGTATCAGCTGAGTGCCACAGG
	6826 6830 6840 6850 6860 6870 6880 6890 6901
H77 (NC_004102)	CGGAAGTCTCGGAGATTCGCCCGGGCCCTGCCCGTTTGGGCGCGGCCGGACTACAACCCCCCGCTAGTAGAGACGT
HCV1	CGGAAGTCTCGGAGATTCCCCCCGGCCCTGCCCATTTGGGCGCGGCCGGACTACAACCCCCCGCTGCTAGAGACGT
HCV2	ATCACGAGGAGGAAGTTCCCACCGGCACTACCTCCCTGGGCCCGTCCAGACTACAACCCTCCTGTGATCGAGACAT
HCV3	AAGAAACCTCCCAAGTATCCTCCAGCCCTTCCTATCTGGGCTAGGCCAGACTACAACCCTCCACTGTTGGACCGCT
HCV4	CGCCCCACCAAGAAGTTCCCGCCGGCCATGCCAATTTGGGCCCGGCCAGATTACAACCCACCTCTGGTTGAGACGT
HCV5	AGGAGGGGACCGGCGTTTCCCCCCGCCCTGCCAGTATGGGCAAGGCCGGGGTATGACCCACCCCTCCTGGAGACTT
HCV6	CCACCCAGGCCTAAATTCCCTCCAGCTCTCCCCATATGGGCCAGGCCTGACTACAACCCACCTCTGCTAGAGACAT

Table S5. *Cont.*

	6902 6910 6920 6930 6940 6950 6960 6970 6977
H77 (NC_004102)	GGAAAAAGCCTGACTACGAACCACCTGTGGTCCATGGCTGCCCGCTACCACCTCCACGGTCCCCTCCTGTGCCTCC
HCV1	GGAAAAAGCCGGACTACGAACCACCTGTGGTCCATGGCTGCCCACTT---CCTCCACAGTCCCCTCCTGTGCCTCC
HCV2	GGAAGAGGCCGGACTATGAACCACCCACTGTCCTAGGCTGTGCCCTC---CCCACACCTCAAGCGCCAGTGCCCCC
HCV3	GGAAAGCACCGGATTATGTACCACCAACTGTCCATGGATGCGCCTTA---CCACGGGGCGCTCCACCGGTGCCTCC
HCV4	GGAAGCAACCGGACTACGAACCTCCGGTCGTTCACGGGTGCGCCCTG---CCCAGTAAGCCGCCCCCCGTTCCTCC
HCV5	GGAAGCGGCCGGATTATGACCCCCCCCAGGTGTCAGGCTGCCCGCTA---CCTGCGGGCCTCCCGCCTGTCCCGCC
HCV6	GGAAGACGCCCGAATACGAGCCTCCAGTGGTGTCTGGGTGTGCCCTG---CCACCTAAACCGACTCCAATTCCTCC
	6978 6990 7000 7010 7020 7030 7040 7053
H77 (NC_004102)	GCCTCGGAAAAAGCGTACGGTGGTCCTCACCGAATCAACCCTATCTACTGCCTTGGCCGAGCTTGCCACCAAAAGT
HCV1	GCCTCGGAAGAAGCGG---GTGGTCCTCACCGAATCAACCGTATCTACTGCCTTGGCCGAGCTTGCCACCAAGAGC
HCV2	ACCTCGGAGGCGCCGC---AAAGTCCTGACTCAGGACAATGTGGAGGGGGTCCTCAGGGAGATGGCCGACAAAGTG
HCV3	CCCTCGGAGGAAAAGA---ATCCAGCTGGATGGCTCCAATGTGTCCGCGGCGCTAGCTGCGCTAGCGGAAAAATCA
HCV4	CCCCAGGAGGAAGCGG---GTCGTGCTCTCCGAGTCCAACGTCTCAGACGCCTTGGCGGACTTGGCAGCGAAGTCC
HCV5	TCCGCGGAGGAAGAGGAAGATGGAACTATCTGACTCTACCGTGTCCCAAGTCATGGCCGACTTGGCGCACGCCAGG
HCV6	ACCGAGGAGGAAGAGGCTGCACCTGGATGAGTCCACGGTCTCACACGCCTTGGCGCAGCTGGCCGAGAAGACGTTT
	7054 7060 7070 7080 7090 7100 7110 7120 7129
H77 (NC_004102)	TTTGGCAGCTCCTCAACTTCCGGCATTACGGGCGACAATACGACAACATCCTCTGAGCCCGCCCCTTCTGGCTGCC
HCV1	TTTGGC---TCCTCAACTTCCGGCATTACGGGCGACAATACGACAACATCCTCTGAGCCCGCCTCCTCTGGCTGC-
HCV2	TTCAGC---CGCGACTCCGGCCATTCCACCGGGGAGGACACCGGAGGGGACACCGGCCAGGACCCCCCTGAGGAG-
HCV3	TTCCCGTCCGAAGAGAATAGCTCATCCTCAGGGGTCGACACACAGTCCAGCACTACTTCCAAGGTGCCCCCTTCT-
HCV4	TTTGGCCAGCCGGACTCCGATTCCGGGGCAGGCCTCACCACCCCAACTGAGACTTCTGACCCAGACCCCATCATT-
HCV5	TTCAAGGCCCCATCCATTGAAGGCCAGGATTCTGCGTTGGGCACTGGCAGCCAACCCGATTCAGGGCCTGAGGAA-
HCV6	CCGGAGACTGCCCCAGCAGCCAGTGCAGACTCGGGACTAGCAACTACCAGTGCCGCTGCAGCGGCCCCAGCAGCA-

Table S5. *Cont.*

	7130 7140 7150 7160 7170 7180 7190 7200 7205
H77 (NC_004102)	CCCCCGACTCCGACGTTGAGTCCTATTCTTCCATGCCCCCCCTGGAGGGGGAGCCTGGGGATCCGGATCTCAGCGA
HCV1	--CCAGACTCCGACGCTGAGTCCTATTCTTCCATGCCCCCCCTGGAGGGGGAGCCTGGGGATCCGGATCTC---AG
HCV2	--GCCGCTTCAGAAACGGGATCACTGTCCTCCATGCCTCCCCTTGAGGGAGAGCCGGGGGACCCTGACCTGGAGTC
HCV3	--GGGGAGTCCGACTCAGAGTCATGCTCGTCCATGCCTCCTCTCGAGGGAGAGCCGGGCGATCCGGACTTG---TG
HCV4	--GACAAATCAGACGACGGGTCTTACTCGTCAATGCCTCCACTTGAAGGGGAGCCCGGTGACCCAGACTTG---TC
HCV5	--GATGACTCGGACGCGGCTTCATATTCTTCCATGCCTCCGCTAGAGGGCGAGCCTGGGGACCCTGACCTT---TC
HCV6	--GCCGCCTCAGAAGCAGAGTCCTACAGCTCCATGCCCCCTCTTGAGGGCGAGCCCGGAGACCCTGACCTA---TC
	7206 7210 7220 7230 7240 7250 7260 7270 7281
H77 (NC_004102)	CGGGTCATGGTCGACGGTCAGTAGTGGGGCCGACACGGAAGATGTCGTGTGCTCAATGTCTTATTCCTGGACAGGC
HCV1	CGGGTCATGGTCGACGGTCAGTAGTGGGGCCGCCACGGAGGATGTCGTGTGCTCAATGTCTTACACCTGGACAGGC
HCV2	TAAGTCGTGGTCCACAGTCTCT------GATCAAGAGGATTCTGTCATCTGCTCCATGTCATACTCCTGGACGGGG
HCV3	CGACTCTTGGTCCACCGTTAGT------AGCGAGGAGCAGAGCGTGGTCTGCTCTATGTCGTACTCTTGGACCGGC
HCV4	AGACTCTTGGTCCACCGTCAGC------TCGGAGGAC------GTAGTGTGTTCAATGTCATACTCATGGACGGGG
HCV5	AGGGTCGTGGTCAACCGTCAGC------GAGGACAGC------GTGGTGTGTTCCATGTCATACAGCTGGACTGGG
HCV6	AGGGTCTTGGTCCACGGTCAGC------GAGGACGAC------GTCGTCTGCTCCATGTCCTACTCCTGGACGGGG
	7282 7290 7300 7310 7320 7330 7340 7350 7357
H77 (NC_004102)	GCACTCGTCACCCCGTGCGCTGCGGAAGAACAAAAACTGCCCATCAACGCACTGAGCAACTCGTTGCTACGCCATC
HCV1	GCACTCATCACCCCGTGCGCCGCGGAAGAACAAAAACTGCCCATCAACGCACTGAGCAACTCGTTGCTGCGTCACC
HCV2	GCCCTCATAACACCATGTGGGCCCGAGGAGGAGAAGTTGCCAATCAACCCTCTGAGTAATTCGCTCATGCGGTTCC
HCV3	GCCCTGATAACACCATGTAGTGCTGAGGAGGAGAAACTGCCCATCAGCCCACTCAGCAACTCCTTGCTGAGACATC
HCV4	GCGCTTGTAACACCCTGCGCAGCTGAGGAGTCCAAGCTGCCAATCAACCCCCTGAGCAACTCACTACTGCGCCACC
HCV5	GCGCTCATCACCCCTTGCTCTGCTGAGGAGGAAAAATTACCTATCAATCCCTTAAGCAACACCCTATTGCGCCACC
HCV6	GCTCTCATCACACCATGTGCTGCGGAGGAGGAGAAGCTGCCAATAAATCCCCTGAGCAACTCCCTCATACGACACC

Table S5. *Cont.*

	7358 7370 7380 7390 7400 7410 7420 7433
H77 (NC_004102)	ACAATCTGGTGTATTCCACCACTTCACGCAGTGCTTGCCAAAGGCAGAAGAAAGTCACATTTGACAGACTGCAAGT
HCV1	ACAATCTGGTGTATTCCACCACCTCACGCAGTGCTTGCCAAAGGCAGAAGAAAGTCACATTTGACAGACTGCAAGT
HCV2	ACAACAAGGTGTACTCCACAACATCGAGGAGTGCCTCTCTGAGGGCAAAGAAGGTGACTTTTGACAGGGTGCAGGT
HCV3	ATAACCTAGTCTATTCAACGTCGTCTAGAAGCGCTTCTCAGCGTCAGAAGAAGGTTACCTTCGACAGACTGCAGGT
HCV4	ACAACATGGTGTACGCCACGACCTCCCGTTCCGCCGCCACCCGGCAGAAGAAGGTCACCTTCGACCGCATGCAGGT
HCV5	ACAATCTTGTGTACTCCACTTCCTCCCGAAGTGCGGGTCAGAGGCAGAAGAAGGTCACCTTTGACAGGCTACAAGT
HCV6	ATAACCTGGTGTATTCCACCACATCACGCAGCGCAGCCCTGCGCCAGAAGAAGGTCACATTTGACCGAGTGCAAGT
	7434 7440 7450 7460 7470 7480 7490 7500 7509
H77 (NC_004102)	TCTGGACAGCCATTACCAGGACGTGCTCAAGGAGGTCAAAGCAGCGGCGTCAAAAGTGAAGGCTAACTTGCTATCC
HCV1	CCTGGACAACCATTACCAGGACGTGCTCAAGGAGGTTAAGGCGGCGGCGTCAAAAGTGAAGGCTAACTTGCTATCC
HCV2	GCTGGACGCACACTATGACTCAGTCTTGCAGGACGTTAAGCGGGCCGCCTCCAAGGTTAGTGCGAGGCTCCTCTCA
HCV3	GCTCGACGACCATTACAAGACTGCATTAAAGGAGGTAAAGGAGCGAGCGTCTAGGGTAAAGGCTCGCATGCTCACC
HCV4	GGTGGACAACCACTACCATGAAGTACTTAAGGAGATTAAGGCACGAGCGTCCAGAGTGAAGGCGCGGACCCTCTCT
HCV5	CCTCGACGACCACTACAGAGAGGTTGTGGACGAGATGAAGCGATTAGCTTCCAAGGTTAAGGCCAGACTACTGCCC
HCV6	GCTCGACCAACATTATCATGATGTACTAAAGGAGATTAAGCTCCGGGCGTCCAAGGTGAAGGCAAAGCTCCTCTCC
	7510 7520 7530 7540 7550 7560 7570 7580 7585
H77 (NC_004102)	GTAGAGGAAGCTTGCAGCCTGACGCCCCCACATTCAGCCAAATCCAAGTTTGGCTATGGGGCAAAAGACGTCCGTT
HCV1	GTAGAGGAAGCTTGCAGCCTGACGCCCCCACATTCAGCCAGATCCAAATTTGGCTATGGGGCAAAAGACGTCCGTT
HCV2	GTAGAGGAAGCCTGCGCGCTGACCCCGCCCCACTCCGCCAGATCACGGTACGGATTTGGGGCAAAGGAGGTGCGCA
HCV3	ATCGAGGAAGCGTGCGCGCTCGTCCCTCCTCACTCTGCCCGGTCGAAGTTCGGGTATAGTGCGAAGGACGTTCGCT
HCV4	GTTGAGGAAGCCTGCGACCTGACGCCCCCCCATTCTGCCAGATCAAAGTTTGGCTACGGGGCAAAGGACGTTCGGA
HCV5	CTAGAGGAAGCTTGTGGGTTAACACCACCCCATTCCGCCCGTTCCAAATACGGATACGGCGCGAAGGAAGTTCGCT
HCV6	ATAGAGGAAGCCTGCGACCTCACCCCACCGCACTCAGCCCGGTCCAAGTTTGGGTATGGGGCGAAGGACGTTAGGA

Table S5. *Cont.*

	7586 7590 7600 7610 7620 7630 7640 7650 7661
H77 (NC_004102)	GCCATGCCAGAAAGGCCGTAGCCCACATCAACTCCGTGTGGAAAGACCTTCTGGAAGACAGTGTAACACCAATAGA
HCV1	GCCATGCCAGAAAGGCCGTAAACCACATCAACTCCGTGTGGAAAGACCTTCTGGAAGACAGTGTAACACCAATAGA
HCV2	GCTTATCCAGGAGGGCCGTTAACCACATCCGGTCCGTGTGGGAGGACCTCCTGGAAGACCAACATACACCAATTGA
HCV3	CCTTGTCCAGCAAGGCCATTAACCAGATCCGCTCCGTCTGGGAGGACTTGCTGGAAGACACCACAACTCCAATTCC
HCV4	GCCATTCCCGCAAGGCCATTAACCACATCAACTCCGTGTGGGAGGACTTGCTGGAAGACAACACTACCCCCATTCC
HCV5	CTCTCGACAAGAAAGCCCTTAAGCACATCGAGGGTGTGTGGCAAGACTTACTGGATGACTCTGATACCCCATTGCC
HCV6	GCCATGCTAGCAAGGCCATTAACCACATCAACTCCGTGTGGGAGGACTTGCTAGAAGACACTGAAACACCAATTCC
	7662 7670 7680 7690 7700 7710 7720 7730 7737
H77 (NC_004102)	CACTACCATCATGGCCAAGAACGAGGTTTTCTGCGTTCAGCCTGAGAAGGGGGGTCGTAAGCCAGCTCGTCTCATC
HCV1	CACC---ATCATGGCTAAGAACGAGGTTTTCTGCGTTCAGCCTGAGAAGGGGGGTCGTAAGCCAGCTCGTCTCATC
HCV2	CACC---ATCATGGCCAAAAATGAGGTGTTCTGTGTTGATCCCACCAAAGGCGGGAAAAAGCCAGCTCGCCTCATC
HCV3	AACC---ATCATGGCGAAGAACGAGGTGTTTTGTGTGGACCCCGTTAAGGGGGGCCGCAAGCCCGCTCGCCTCATT
HCV4	AACA---ATCATGGCCAAGAATGAGGTCTTCTCTGTGAAACCGGAGAAGGGCGGCCGAAAGCCAGCTCGTCTAATC
HCV5	GACC---ATCATGGCAAAAAACGAGGTGTTCGCAGTGGAGCCGTCCAAGGGGGGGAAGAAGCCAGCACGGCTAATA
HCV6	TACC---ATCATGGCCAAGAATGAGGTCTTCTGCGTGGATCCGTCGAAGGGGGGACGCAAGCCGGCACGCCTGATC
	7738 7750 7760 7770 7780 7790 7800 7813
H77 (NC_004102)	GTGTTCCCCGACCTGGGCGTGCGCGTGTGCGAGAAGATGGCCCTGTACGACGTGGTTAGCAAGCTCCCCCTGGCCG
HCV1	GTGTTCCCCGACCTGGGTGTGCGCGTGTGCGAGAAGATGGCCCTGTACGACGTGGTCAGCAAGCTCCCTCTGGCCG
HCV2	GTATACCCTGACCTTGGGGTCAGGGTGTGCGAGAAGATGGCCCTCTATGACATTGCACAAAAGCTTCCCAAGGCAG
HCV3	GTGTACCCTGACCTGGGGGTGCGTGTCTGTGAGAAACGCGCCCTATATGACGTGATACAGAAGTTGTCAATTGAGA
HCV4	GTATACCCGGACCTGGGGGTGCGGGTGTGCGAGAAGAGGGCGCTTTATGACGTCGTCAAAAAACTTCCTAAGGCCG
HCV5	GTCTACCCCGACCTTGGAGTCCGCGTCTGCGAGAAGCGGGCATTGTACGACATAGCTCAAAAACTGCCGACAGCCC
HCV6	GTTTACCCGGACCTAGGCGTGCGGGTGTGCGAGAAGAGGGCCCTATACGACATCACGCGGAAGTTACCACTGGCCG

Table S5. *Cont.*

	7814 7820 7830 7840 7850 7860 7870 7880 7889
H77 (NC_004102)	TGATGGGAAGCTCCTACGGATTCCAATACTCACCAGGACAGCGGGTTGAATTCCTCGTGCAAGCGTGGAAGTCCAA
HCV1	TGATGGGAAGCTCCTACGGATTCCAATACTCACCAGGACAGCGGGTTGAATTCCTCGTGCAAGCGTGGAAGTCCAA
HCV2	T**A**ATGGG**GCCA**TCCTA**T**GG**G**TTCCAATACTC**T**CC**T**G**C**A**G**A**A**CGGGT**C**GA**T**TT**T**CTC**C**T**CA**AAGC**T**TGG**GGAAGT**AA
HCV3	**C**GATGGG**TCCTG**C**T**TA**T**GGATTCCAATACTC**G**CC**TCAG**CAGCGGGT**C**GAA**CGT**CT**GC**TG**A**A**GAT**GTGGA**CC**TC**A**AA
HCV4	TGATGGGA**GC**C**G**C**T**TACGG**C**TTCCAATACTCACCAG**CG**CAGCGGGT**C**GAAT**A**CCTC**T**TG**A**AAGC**C**TGGA**G**GTC**A**AA
HCV5	T**T**ATGGG**GCC**CTC**G**TA**T**GG**G**TT**T**CA**G**TACTC**C**CCAG**CG**CAGCGGGTTGAATTC**T**T**AC**TG**A**A**G**GCGTGGAAGTCCAA
HCV6	TGATGGGA**GCAG**C**A**TACGG**C**TTCCA**G**TACTC**C**CC**TAAG**CAG**A**GGGTTGA**G**T**A**C**T**T**GC**T**CA**AA**AT**GTGG**CGC**TC**A**AA
	7890 7900 7910 7920 7930 7940 7950 7960 7965
H77 (NC_004102)	GAAGACCCCGATGGGGTTCTCGTATGATACCCGCTGTTTTGACTCCACAGTCACTGAGAGCGACATCCGTACGGAG
HCV1	GAAGACCCC**A**ATGGGGTTCTC**A**TATGATACCCGCTG**C**TTTGACTCCACAGTCACTGAGAGCGACATCCGTACGGAG
HCV2	GAAG**GA**CCC**A**ATGGGGTTCTCGTATGA**C**ACCCGCTG**C**TTTGACTC**A**AC**C**GTCAC**G**GAGAG**G**GACAT**AA**G**A**AC**A**GAG
HCV3	GAA**A**ACCCC**CT**TGGGGTTCTCGTATGA**C**ACCCGCTG**C**TTTGACTC**G**AC**T**GTCACTGA**ACAG**GACATC**A**G**GGT**GGA**A**
HCV4	GAAG**GTT**CC**C**ATGGGGTTCTC**A**TATGA**C**ACCCGCTG**C**TTTGACTCCAC**T**GT**G**ACTGA**A**AG**G**GACATC**A**G**GGTC**GAG
HCV5	**A**AAGACCCC**C**ATGG**CC**TTCTC**C**TATGA**C**ACCCGCTG**C**TTTGACTC**A**AC**C**GT**T**AC**C**GA**ACAT**GACAT**AATG**AC**T**GA**A**
HCV6	GAAGAC**G**CC**T**ATGGG**C**TTCTC**T**TATGA**C**ACC**A**G**G**TGTTTTGACTC**A**ACAGTCAC**C**GAG**C**GCGACATCCG**G**AC**T**GAG
	7966 7970 7980 7990 8000 8010 8020 8030 8041
H77 (NC_004102)	GAGGCAATTTACCAATGTTGTGACCTGGACCCCCAAGCCCGCGTGGCCATCAAGTCCCTCACTGAGAGGCTTTATG
HCV1	GAGGCAAT**C**TACCAATGTTGTGACCTGGACCCCCAAGCCCGCGTGGCCATCAAGTCCCTCACTGAGAGGCTTTATG
HCV2	GA**AT**C**C**AT**A**TA**T**CA**GGC**TTGT**TC**CCTG**CCTGAGG**A**G**GCC**A**G**AACT**GCCAT**AC**A**C**TC**G**CTCACTGAGAG**A**CT**C**TA**C**G
HCV3	GAGG**AG**AT**A**TACCAATG**C**TGT**A**ACCT**T**GA**A**CC**GG**A**G**GCC**A**G**GAAA**G**TG**ATC**TCC**TCCCTCAC**G**GAG**C**GGCTTTA**CT**
HCV4	GAGG**AGG**T**C**TA**T**CA**G**TGTTGTGACCTGGA**G**CCC**G**AAGCCCGC**AA**GG**TA**AT**ATCCG**CCCTCAC**A**GAGAG**A**CT**C**TA**C**G
HCV5	GAG**T**C**C**ATTTACCAAT**CA**TGTGAC**T**TG**C**A**G**CC**TG**A**G**GC**G**CG**C**GGC**A**AT**ACG**GTC**A**CTCAC**CC**A**AC**G**C**CT**A**TA**CT**
HCV6	**C**A**C**G**AC**AT**C**TACCA**GTC**TTG**CC**A**G**CTGGACCC**AG**A**G**GCC**A**G**GAA**GGCCAT**A**A**CA**TCCCT**G**AC**A**GA**A**AGGCT**C**TA**C**G

Table S5. *Cont.*

	8042 8050 8060 8070 8080 8090 8100 8110 8117
H77 (NC_004102)	TTGGGGGCCCTCTTACCAATTCAAGGGGGGAAAACTGCGGCTACCGCAGGTGCCGCGCGAGCGGCGTACTGACAAC
HCV1	T**C**GGGGGCCCTCTTACCAATTCAAGGGGGGA**G**AACTGCGGCTA**T**CGCAGGTGCCGCGCGAGCGGCGTACTGAC**G**AC
HCV2	T**A**GG**A**GG**G**CC**CA**T**G**AC**A**AA**CAGC**A**A**GGGG**C**AA**TC**CTGCGG**T**TAC**A**G**GC**G**T**TGCCGCGC**A**AGCGG**T**GT**TT**T**C**ACC**C**AC
HCV3	**GC**GGGGGCCCT**A**T**GTT**CAA**CAGC**A**A**GGGGG**CCC**A**G**TG**T**GG**T**TA**T**CGC**C**G**T**TGCCG**T**GC**C**AG**T**GG**A**GT**T**CTG**C**C**T**AC
HCV4	T**G**GG**C**GGCCC**CA**T**GTAT**AA**CAGC**A**A**GGG**A**GA**CCTT**TGCGG**G**TA**T**CG**G**AG**A**TGCCGCGC**A**AGCGGCGT**CTAC**ACC**C**AC
HCV5	**G**TGG**A**GGCCC**CA**T**GTAT**AA**CAGC**A**A**GGGG**C**AA**C**A**A**TG**T**GG**T**TA**T**CG**T**AG**A**TGCCGCGC**C**AGCGGCGT**CT**T**C**ACC**C**AC
HCV6	TTGG**T**GGCCC**CA**T**GTT**CAA**C**TCAA**A**GGG**CC**A**GTC**CTG**T**GGCTACCGCAG**A**TGCCGCGC**C**AG**T**GG**G**GT**G**CT**CC**C**C**AC
	8118 8130 8140 8150 8160 8170 8180 8193
H77 (NC_004102)	TAGCTGTGGTAACACCCTCACTTGCTACATCAAGGCCCGGGCAGCCTGTCGAGCCGCAGGGCTCCAGGACTGCACC
HCV1	TAGCTGTGGTAACACCCTCACTTGCTACATCAAGGCCC**CA**GCAGCCTGTCGAGCCGCAGGGCTCCAGGACTGCACC
HCV2	**C**AGC**ATG**GG**G**AACACC**A**T**G**AC**A**TGCTACATCAA**A**GCCC**TT**GCAGC**A**TG**CAA**AGC**T**GCAGGG**A**TC**GT**GGAC**CC**CAC**T**
HCV3	**C**AGCT**TC**GG**C**AACAC**AA**TCACTTG**T**TACATCAAGGCC**ACA**GC**G**GC**TGCAA**G**G**GCCGCAGG**C**CTCC**G**G**A**AC**CCGGA**C
HCV4	**C**AGCT**TC**GG**G**AACAC**A**CT**G**AC**G**TGCTA**TC**T**T**AAGGCC**ACA**GCAGCC**ACCA**G**G**GCCGCAGG**C**CT**AA**AGGACTGCACC
HCV5	TAG**TATG**GG**C**AACACC**A**T**G**AC**G**TGCTACAT**T**AAGGC**TTTA**GC**CT**CCTGT**A**GAGC**T**GCA**AA**GCTCC**G**GGACTGCAC**G**
HCV6	**C**AGC**ATG**GG**C**AACACC**A**TCAC**A**TGCTAC**C**T**G**AA**A**GC**A**C**A**GGC**C**GCCTG**CA**G**G**GC**A**GCCGG**C**CTC**A**AGGACT**TTGA**C
	8194 8200 8210 8220 8230 8240 8250 8260 8269
H77 (NC_004102)	ATGCTCGTGTGTGGCGACGACTTAGTCGTTATCTGTGAAAGTGCGGGGGTCCAGGAGGACGCGGCGAGCCTGAGAG
HCV1	ATGCTCGTGTGTGGCGACGAC**C**TAGTCGTTATCTGTGAAAGTGCGGG**A**GTCCAGGAGGACGCGGCGAGCCTGAGAG
HCV2	ATGCT**G**GTGTGTGG**A**GACGAC**C**T**G**GTCGT**C**ATCT**CA**GA**G**AG**CCAA**GGG**AA**C**G**AGGAGGACG**A**G**CGA**A**A**CCTGAGAG
HCV3	**T**T**T**CT**T**GT**C**TG**C**GG**A**GA**T**GA**TC**T**G**GTCGT**GG**T**GGC**TGA**G**AGTG**AT**GG**C**GTC**G**AGGAGGA**TAGA**GC**AGC**CCTGAGAG
HCV4	ATGCT**G**GT**C**TG**C**GG**T**GACGACTTAGTCGTTATC**GCC**GAAAG**C**G**AT**GG**C**GT**GG**AGGAGGAC**AACCGAGC**CCT**CG**GAG
HCV5	**C**T**C**CT**G**GTGTGTGG**T**GACGAC**C**T**T**GT**G**G**CC**ATCTG**C**GA**G**AG**CCA**GGGG**ACA**CA**C**GAGGA**T**G**AA**GCGAGCCTGAGAG
HCV6	ATG**T**T**G**GT**C**TG**C**GG**A**GACGA**T**TT**G**GT**G**GT**C**AT**TT**GTGA**G**AGTGC**T**GG**C**GTCCAGGAGGAC**A**C**T**GC**TGC**CCTG**C**GAG

Table S5. *Cont.*

	8270 8280 8290 8300 8310 8320 8330 8340 8345
H77 (NC_004102)	CCTTCACGGAGGCTATGACCAGGTACTCCGCCCCCCCCGGGGACCCCCCACAACCAGAATACGACTTGGAGCTTAT
HCV1	CCTTCACGGAGGCTATGACCAGGTACTCCGCCCCCCCCGGGGACCCCCCCCAACCAGAATACGACTTGGAGCTCAT
HCV2	CCTTCACGGAGGCTATGACCAGGTATTCCGCCCCTCCCGGTGACCTTCCCAGACCGGAATATGACTTGGAGCTTAT
HCV3	CCTTCACGGAGGCTATGACCAGGTATTCTGCTCCACCCGGAGATGCTCCACAGCCCACCTACGACCTTGAGCTCAT
HCV4	CCTTCACGGAGGCTATGACGAGGTACTCGGCTCCCCCCGGAGACGCCCCGCAACCAGCATATGACCTGGAGCTCAT
HCV5	CCTTCACGGAGGCTATGACCAGGTACTCTGCTCCCCCGGGTGATCCCCCGGTTCCTGCTTATGACCTGGAGCTCGT
HCV6	CATTCACGGATGCTATGACCAGGTACTCAGCTCCCCCTGGAGACGAACCGCACCCAACGTATGACCTGGAGCTGAT
	8346 8350 8360 8370 8380 8390 8400 8410 8421
H77 (NC_004102)	AACATCATGCTCCTCCAACGTGTCAGTCGCCCACGACGGCGCTGGAAAGAGGGTCTACTACCTTACCCGTGACCCT
HCV1	AACATCATGCTCCTCCAACGTGTCAGTCGCCCACGACGGCGCTGGAAAGAGGGTCTACTACCTCACCCGTGACCCC
HCV2	AACATCCTGCTCCTCAAACGTATCGGTAGCGCTGGACCCTCGGGGTCGCCGCCGGTACTACCTAACCAGAGACCCT
HCV3	TACATCTTGCTCCTCCAACGTCTCCGTGGCACGGGACGACAAGGGGAAGAGGTACTATTACCTCACCCGTGATGCC
HCV4	AACATCATGCTCATCCAATGTCTCGGTCGCACACGACGCGACCGGCAAGAGGGTCTACTACCTGACCCGCGACCCT
HCV5	AACATCTTGCTCCTCAAACGTGTCCGTCGCCCGCGATGCTTCAGGCAACAGAGTTTACTACCTCACGCGCGACCCC
HCV6	AACATCGTGCTCCTCCAACGTCTCCGTAGCCCACGACGGCACTGGAAAGAGATATTACTACCTCACACGTGACCCA
	8422 8430 8440 8450 8460 8470 8480 8490 8497
H77 (NC_004102)	ACAACCCCCCTCGCGAGAGCCGCGTGGGAGACAGCAAGACACACTCCAGTCAATTCCTGGCTAGGCAACATAATCA
HCV1	ACAACCCCCCTCGCGAGAGCTGCGTGGGAGACAGCAAGACACACTCCAGTCAATTCCTGGCTAGGCAACATAATCA
HCV2	ACCACTCCAATCGCCCGAGCTGCTTGGGAAACAGTAAGACACTCCCCTGTCAATTCTTGGCTGGGAAACATCATCC
HCV3	ACTACTCCCCTGGCCCGTGCGGCTTGGGAGACAGCTCGTCACACTCCAGTTAACTCCTGGTTGGGCAACATCATCA
HCV4	GAGACTCCCCTGGCACGAGCTGCCTGGGAGACAGCCCGACACACTCCAGTCAACTCCTGGTTGGGGAACATCATAC
HCV5	CAAGTACCACTTGCGAGGGCCGCGTGGGAAACTGCCAAACACTCCCCGGTTAACTCCTGGTTAGGGAACATCATCA
HCV6	ACCACTCCACTTGCGCGGGCCGCTTGGGAGACAGCCCGCCACACTCCAGTCAATTCGTGGTTGGGCAACATCATTA

Table S5. *Cont.*

	8498 8510 8520 8530 8540 8550 8560 8573
H77 (NC_004102)	TGTTTGCCCCCACACTGTGGGCGAGGATGATACTGATGACCCATTTCTTTAGCGTCCTCATAGCCAGGGATCAGCT
HCV1	TGTTTGCCCCCACA**T**TGTGGGCGAGGATGATACTGATGACCCA**C**TTCTTCAGCGTCCTCATAGCCAGGGATCAGCT
HCV2	**AA**T**AC**GCCCC**T**ACA**A**T**C**TGGG**TCC**GGATG**G**T**CA**T**A**ATGACCCA**C**TTCTTC**TC**C**A**T**A**CT**AT**T**G**GCC**CA**GGA**CACT**CT
HCV3	TGT**AC**GC**G**CC**T**AC**CA**T**C**TGGG**T**G**C**G**C**ATG**G**TA**A**TGATGAC**A**CA**C**TT**T**TTC**TC**C**A**T**A**CTC**CA**A**T**CC**CA**GGA**GATA**CT
HCV4	T**C**T**AC**GC**G**CCCAC**CA**T**A**TGGG**T**G**C**G**C**ATG**G**TA**T**TGATGACCCA**C**TTCTTC**TCAA**T**A**CTC**CA**A**AG**C**CA**GGA**AGCC**CT
HCV5	TGT**AC**GC**G**CC**G**AC**C**CTGTGGGC**TC**GGAT**AG**T**G**CTGATGAC**G**CA**C**TTCTTCAGCGT**A**CT**GCAGAG**C**CA**GGA**G**CA**A**CT
HCV6	TGT**A**TGCCCCCAC**GA**T**A**TGGG**T**G**C**G**C**ATG**G**T**T**CTGATGACCCATTTCTTC**TC**C**A**T**A**CTC**CAG**GC**GCA**GGA**A**CAGCT

	8574 8580 8590 8600 8610 8620 8630 8640 8649
H77 (NC_004102)	TGAACAGGCTCTTAACTGTGAGATCTACGGAGCCTGCTACTCCATAGAACCACTGGATCTACCTCCAATCATTCAA
HCV1	TGAACAGGC**C**CTT**G**A**T**TG**C**GAGATCTACGG**G**GCCTGCTACTCCATAGAACCACTGGA**C**CTACCTCCAATCATTCAA
HCV2	**GA**A**C**CA**AAA**TCT**C**AA**T**T**T**TGAGAT**G**TACGG**G**GC**AGTA**TA**T**TC**GG**T**CA**A**T**CCA**T**TGGA**C**CTACC**AG**C**C**AT**A**ATT**G**AA
HCV3	TGA**T**C**GAC**C**C**CTT**G**ACT**T**TGA**A**AT**G**TACGG**G**GCC**ACT**TACTC**TG**T**CACT**CC**G**CTGGAT**T**TACC**AG**CAATCATT**G**AA
HCV4	TGAA**A**A**A**GC**A**CT**CG**ACT**TC**GA**C**AT**G**TACGGAG**T**C**ACA**TACTC**T**AT**CACT**CC**G**CTGGATCT**G**CC**AG**C**C**AT**A**ATTCAA
HCV5	**G**GAA**A**A**AA**C**C**CT**AGCA**T**T**TGA**A**AT**G**TA**T**GG**GT**C**AGT**CTACTC**TG**T**CACT**CC**G**CTGGATCT**T**CC**GG**C**T**ATCATTCA**G**
HCV6	**G**GA**CA**A**A**GC**A**CT**CG**ACT**TC**GA**C**ATCTACGGAG**T**C**AC**CTA**T**TC**AG**T**CACT**CCACT**T**GATCT**C**CC**AG**CAATCATTCAA

	8650 8660 8670 8680 8690 8700 8710 8720 8725
H77 (NC_004102)	AGACTCCATGGCCTCAGCGCATTTTCACTCCACAGTTACTCTCCAGGTGAAATCAATAGGGTGGCCGCATGCCTCA
HCV1	AGACTCCATGGCCT**T**AGCGCATTTTCACTCCACAGTTACTCTCCAGGTGAAATCAATAGGGTGGCCGCATGCCTCA
HCV2	AG**G**CT**A**CATGG**G**CT**TGA**CGC**C**TTTTCACT**G**CACA**CA**TACTCTCC**CCAC**GAA**C**TC**TCAC**GGGTGGC**A**GCA**ACT**CTCA
HCV3	AGACTCCATGG**T**CT**A**AGCGC**G**TT**CA**C**G**CTCCACAGTTACTCTCCAG**TA**GA**GC**TCAATAGGGT**C**GC**GGGGACA**CTCA
HCV4	AGACTCCATGGC**T**T**G**AGCGCATTT**A**CACT**G**CAC**G**G**A**TACTCTCCA**CAC**GAA**C**TCAAT**C**GGGTGGC**A**G**G**A**A**GCCTCA
HCV5	AGACTCCATGG**A**CT**T**AGCGC**G**TTTTCACTCCA**TTCA**TACTCTCC**GA**G**C**GAAATCAATAGGGTGGC**TAGT**TGCCTCA
HCV6	**C**GACTCCATGGC**A**T**GGC**CGC**T**TTTTCACTCCA**TG**G**A**TACTCTCCAGGTGAA**C**TCAAT**C**GGGTGG**GA**GCATGCCTCA

Table S5. *Cont.*

	8726 8730 8740 8750 8760 8770 8780 8790 8801
H77 (NC_004102)	GAAAACTTGGGGTCCCGCCCTTGCGAGCTTGGAGACACCGGGCCCGGAGCGTCCGCGCTAGGCTTCTGTCCAGAGG
HCV1	GGAAACTTGGGGTCCCGCCCTTGCGAGCTTGGAGACACCGGGCCCGGAGTGTCCGCGCTAAGCTTCTGTCCAGAGG
HCV2	GAAAACTTGGAGCGCCTCCCCTTAGAGCGTGGAAGAGTCGGGCGCGTGCTGTGAGGGCCTCACTCATCGCCCAG--
HCV3	GGAAGCTTGGGTGCCCCCCCTACGAGCTTGGAGACATCGGGCACGAGCAGTGCGCGCCAAGCTTATCGCCCAGGG
HCV4	GGAAACTTGGGGTACCCCCATTGAGAGCGTGGAGACATCGGGCCCGAGCAGTCCGCGCCAAGCTCATCGCCCAGGG
HCV5	GGAAGCTTGGGGTACCCCCTTTGAGAGCTTGGAGACATCGGGCTCGAGCTGTACGCGCTAAGCTCATCGCCCAGGG
HCV6	GGAAACTTGGGGCACCTCCCTTGAGAGCCTGGAGACATCGAGCCAGAGCGGTCAGAGCCAAGCTCATTGCCCAGGG
	8802 8810 8820 8830 8840 8850 8860 8870 8877
H77 (NC_004102)	AGGCAGGGCTGCCATATGTGGCAAGTACCTCTTCAACTGGGCAGTAAGAACAAAGCTCAAACTCACTCCAATAGCG
HCV1	AGGCAGGGCTGCCATATGTGGCAAGTACCTCTTCAACTGGGCAGTAAGGACAAAGCTCAAACTCACTCCAATAGCG
HCV2	--
HCV3	AGGGAAGGCCAAAATATGCGGCCTTTATCTCTTTAATTGGGCGGTACGCACCAAGACCAAACTCACTCCATTGCCA
HCV4	AGGGAGAGCCAGAATCTGTGGCATATACCTCTTTAACTGGGCGGTAAAAACCAAACTCAAACTCACTCCATTGCCC
HCV5	AGGTAGGGCCGCCATCTGCGGCATCTATCTCTTCAACTGGGCGGTGAAGACAAAACGCAAACTCACTCCATTAGCT
HCV6	GGGGAAAGCCGCTATATGCGGCAAGTACCTCTTCAACTGGGCGGTGAAGACCAAGCTCAAACTCACTCCATTGCGC
	8878 8890 8900 8910 8920 8930 8940 8953
H77 (NC_004102)	GCCGCTGGCCGGCTGGACTTGTCCGGTTGGTTCACGGCTGGCTACAGCGGGGGAGACATTTATCACAGCGTGTCTC
HCV1	GCCGCTGGCCAGCTGGACTTGTCCGGCTGGTTCACGGCTGGCTACAGCGGGGGAGACATTTATCACAGCGTGTCTC
HCV2	--
HCV3	GCCGCTGGCCAGTTGGATTTATCCAGCTGGTTTACGGTTGGCGTCGGCGGGAACGACATTTATCACAGCGTGTCAC
HCV4	GCTGCTGCCAAACTCGACTTGTCGAGTTGGTTTACGGTGGGCGCCGGCGGGGGGGACATTTATCACAGCGTGTCCC
HCV5	GACGCTGACCGGCTGGACTTGTCCAGCTGGTTCACCGTTGGCGCTGGCGGGGGGGACATTTATCACAGCATGTCCC
HCV6	GCCGCGACCAACCTTGACTTGTCAGGCTGGTTCGTGGCAGGCTACAGCGGGGGGGACATTTATCACAGCGTGTCCC

Table S5. *Cont.*

		8954	8960	8970	8980	8990	9000
H77 (NC_004102)	ATGCCCGGCCCCGCTGGTTCTGGTTTTGCCTACTCCTGCTCGCTGCA						
HCV1	**G**TGCCCGGCCCCGCTGGTTCTGGTTTTGCCTACTCCTGCT**T**GCTGCA						
HCV2	--						
HCV3	**G**TGCCCG**AA**CCCGC**CAT**TT**GCT**G**C**TTTGCCTACTCCT**A**CT**AA**C-G**T**A						
HCV4	**G**TGCCCG**A**CCCCGCT**AT**TT**ACTCC**T**G**TGCCTACTCCT**A**CT**TT**C**C**G**T**A						
HCV5	**G**TGCCCG**A**CCCCGCT**AC**TT**ACTCC**T**G**TGCCTACTCCT**A**CT**TAGC**G**T**A						
HCV6	**GG**GC**T**CG**A**CCCCGC**ATA**TT**ACTCC**T**G**TGCCTACTCCT**A**CT**CA**C**C**G**T**A						

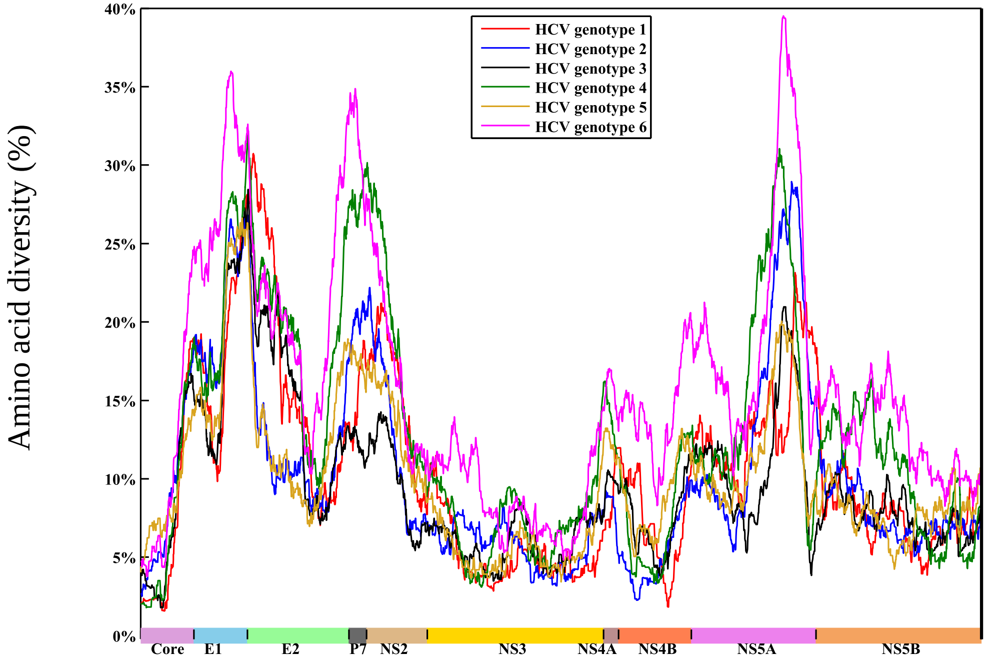

Figure S1. Full-genome sliding window plot for within-genotype amino acid diversity. A sliding window of 100 amino acid positions with a step size of one amino acid was used. The six genotypes were plotted separately in color-coded solid lines.

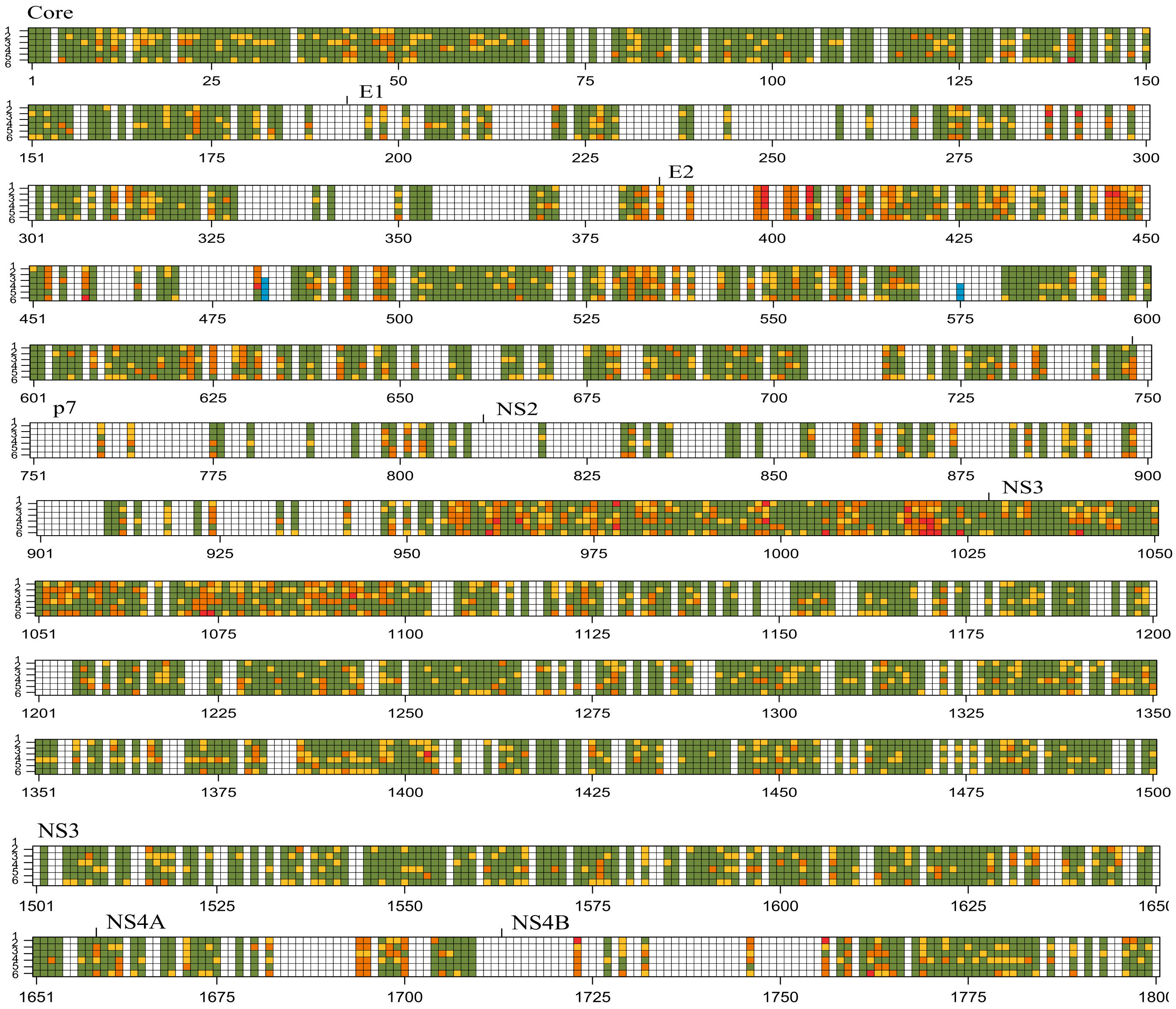

Figure S2. *Cont.*

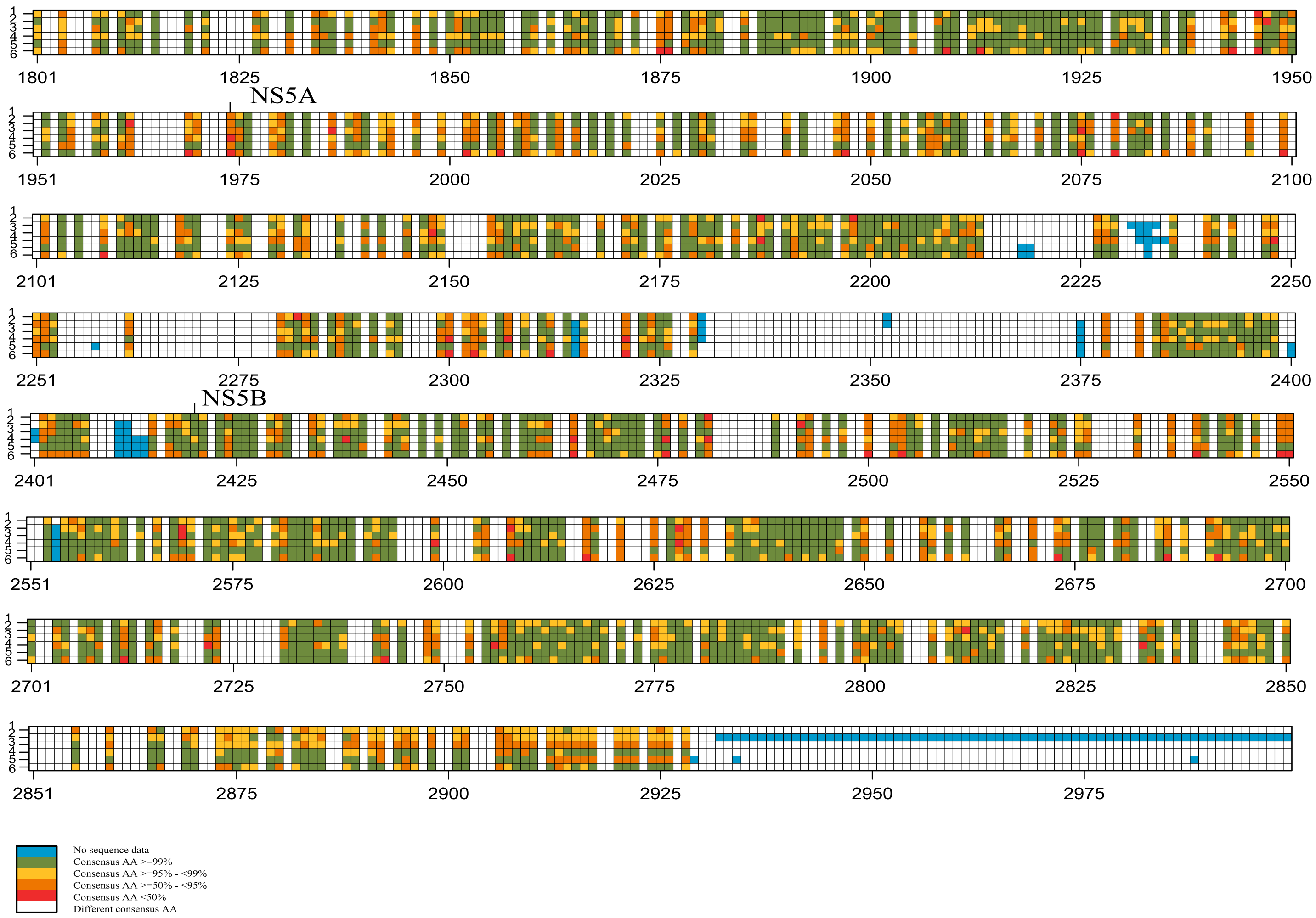

Figure S2. Discretized frequencies of pan-genotypic consensus positions in the HCV full-genome, on amino acid level. The distribution of positions that shared a consensus amino acid across genotypes 1-6, aligned against the general reference sequence H77, is shown for the whole HCV genome. HCV genotype 1 is placed at the top and each square represents a single position. Positions that shared a consensus amino acid across all six genotypes were colored according to the frequency of the consensus amino acid in the respective genotype (for frequency x: category x<50% in red, 50% ≤ x < 95% in orange, 95% ≤ x < 99% in yellow and x ≥ 99% in green). Positions with different consensus amino acids were colored white and positions with no sequence data or a deletion were indicated in blue. It can be seen that HCV2 sequences of NS5B are shorter compared to other HCV genotypes.

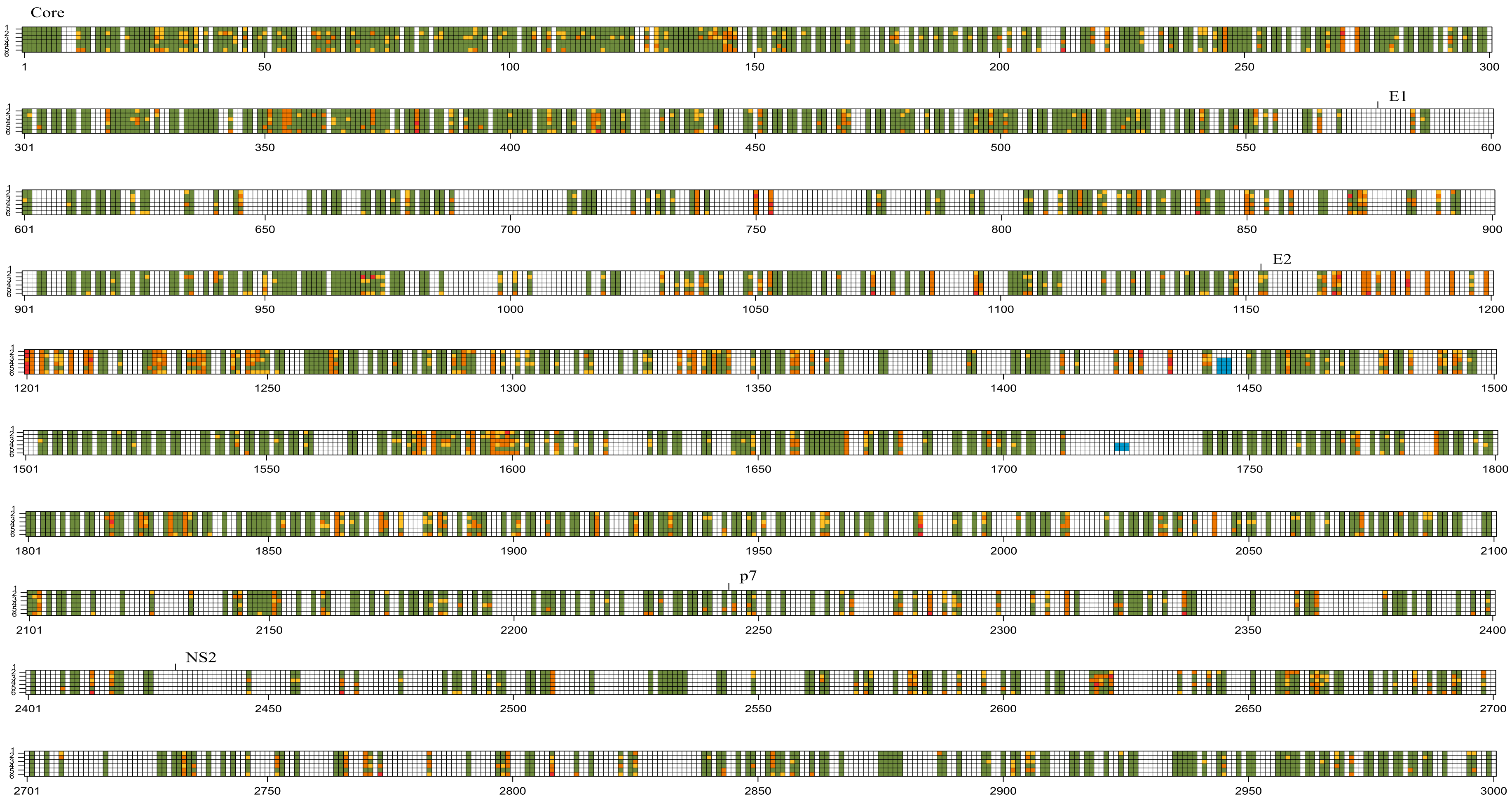

Figure S3. *Cont.*

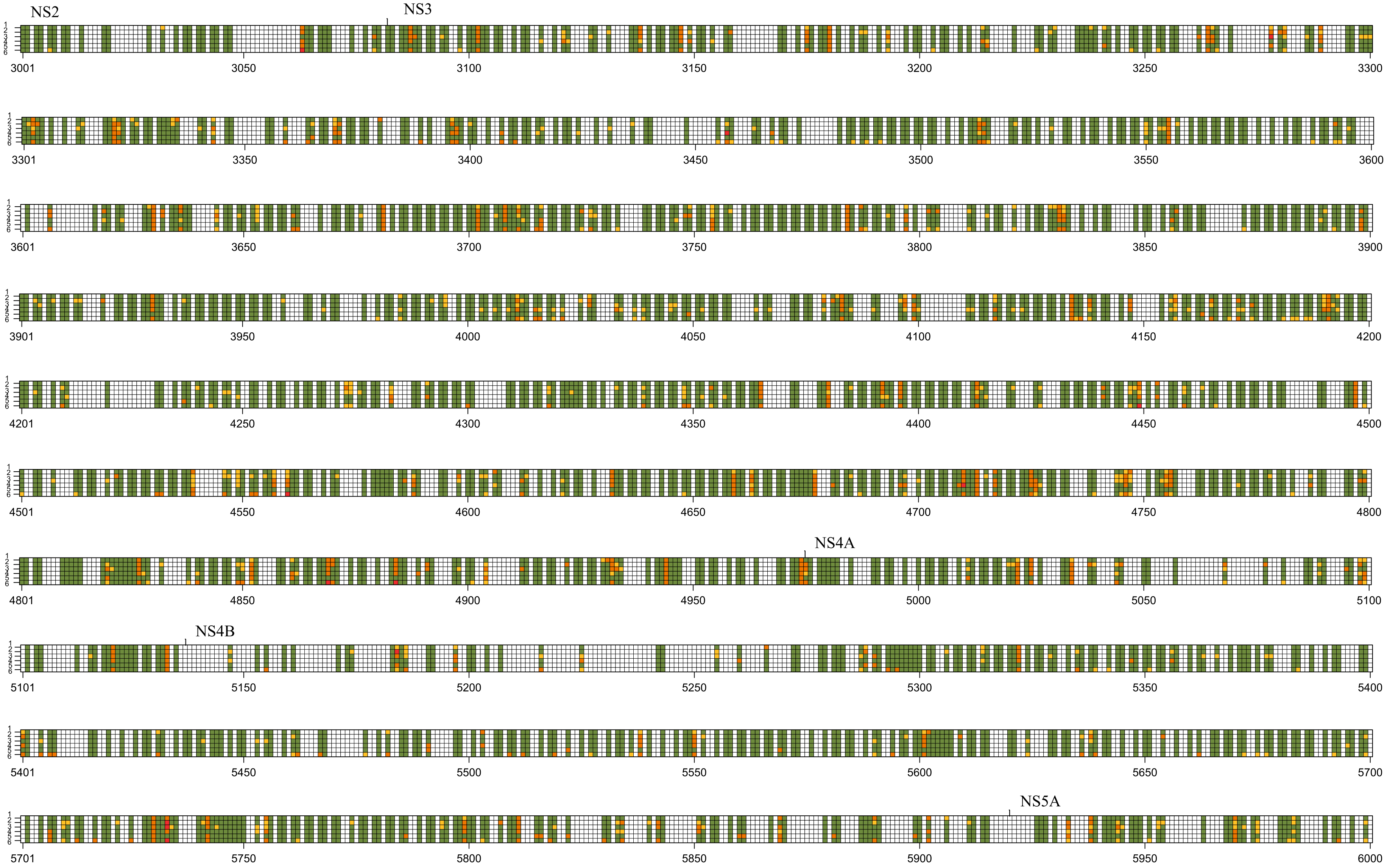

Figure S3. *Cont.*

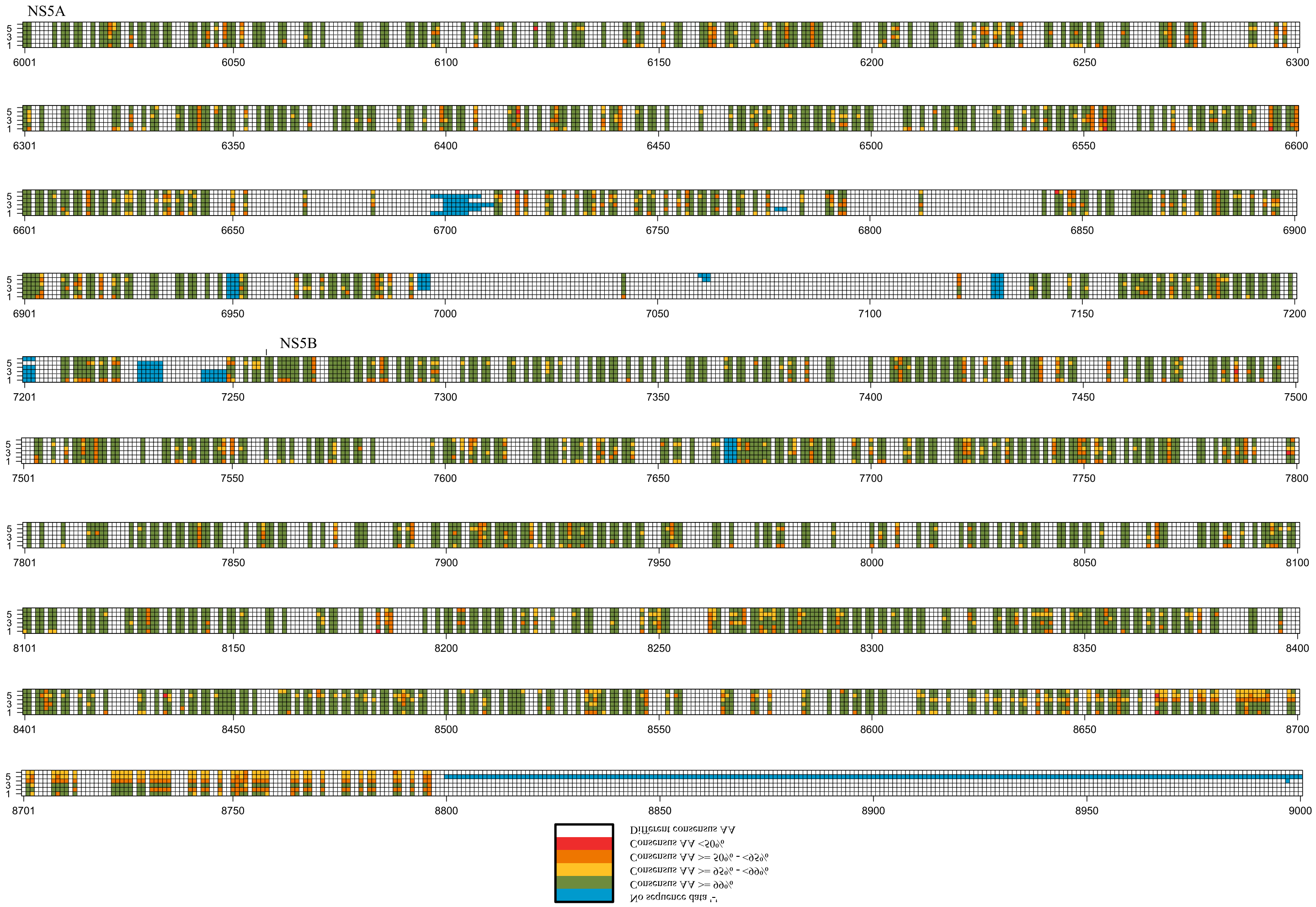

Figure S3. Discretized frequencies of pan-genotypic consensus positions in the HCV full-genome, on nucleotide level. The distribution of positions that shared a consensus nucleotide across genotypes 1–6, aligned against the general reference sequence H77 , is shown for the whole HCV genome. Genotype 1 is placed at the top and each square represents a single position. Positions that shared a consensus nucleotide in all six genotypes were colored according to the frequency of the consensus nucleotide in the respective genotype (for frequency x: category x < 50% in red, 50% ≤ x < 95% in orange, 95% ≤ x < 99% in yellow and x ≥ 99% in green). Positions with different consensus nucleotides were colored white and positions with no sequence data or a deletion were indicated in blue. It can be seen that HCV2 sequences of NS5B are shorter compared to other genotypes.

Table S6. Amino acid alignment of the H77 (NC_004102) reference sequence with the consensus of all six HCV genotypes. For each genotype, a consensus alignment was obtained, based on the most frequently occurring amino acids in the respective datasets. Consensus amino acids indicated in bold, highlight positions that display a different consensus amino acid compared to reference sequence H77.

	1 10 20 30 40 50 60 70 80
H77 (NC_004102)	MSTNPKPQRKTKRNTNRRPQDVKFPGGGQIVGGVYLLPRRGPRLGVRATRKTSERSQPRGRRQPIPKARRPEGRTWAQPG
HCV1	MSTNPKPQRKTKRNTNRRPQDVKFPGGGQIVGGVYLLPRRGPRLGVRATRKTSERSQPRGRRQPIPKARRPEGRTWAQPG
HCV2	MSTNPKPQRKTKRNTNRRPQDVKFPGGGQIVGGVYLLPRRGPRLGVRATRKTSERSQPRGRRQPIPK**D**RR**ST**G**KS**W**GK**PG
HCV3	MST**L**PKPQRKTKR**I**TNRRPQDVKFPGGGQIVGGVY**V**LPRRGPRLGVRATRKTSERSQPRGRRQPIPKARR**S**EGR**S**WAQPG
HCV4	MSTNPKPQRKTKRNTNRRP**M**DVKFPGGGQIVGGVYLLPRRGPRLGVRATRKTSERSQPRGRRQPIPKARRPEGR**S**WAQPG
HCV5	MSTNPKPQRKTKRNTNRRPQDVKFPGGGQIVGGVYLLPRRGPRLGVRATRKTSERSQPRGRRQPIPKAR**Q**P**T**GR**S**W**G**QPG
HCV6	MST**L**PKPQRKTKRNTNRRP**M**DVKFPGGGQIVGGVYLLPRRGPRLGVRATRKTSERSQPRGRRQPIPKAR**Q**P**T**GR**H**WAQPG
	Core
	81 90 100 110 120 130 140 150 160
H77 (NC_004102)	YPWPLYGNEGCGWAGWLLSPRGSRPSWGPTDPRRRSRNLGKVIDTLTCGFADLMGYIPLVGAPLGGAARALAHGVRVLED
HCV1	YPWPLYGNEGCGWAGWLLSPRGSRPSWGPTDPRRRSRNLGKVIDTLTCGFADLMGYIPLVGAPLGGAARALAHGVRVLED
HCV2	YPWPLYGNEGCGWAGWLLSPRGSRP**T**WGPTDPR**H**RSRNLGKVIDTLTCGFAD**I**MGYIPLVGAP**V**GG**V**ARALAHGVRVLED
HCV3	YPWPLYGNEGCGWAGWLLSPRGSRPSWGP**N**DPRRRSRNLGKVIDTLTCGFADLMGYIPLVGAP**V**GG**V**ARALAHGVR**A**LED
HCV4	YPWPLYGNEGCGWAGWLLSPRGSRPSWGP**N**DPRRRSRNLGKVIDTLTCGFADLMGYIPLVGAP**V**GG**V**ARALAHGVR**AV**ED
HCV5	YPWPLY**A**NEG**L**GWAGWLLSPRGSRP**N**WGP**N**DPRR**K**SRNLGKVIDTLTCGFADLMGYIPLVG**G**P**V**GG**V**ARALAHGVRVLED
HCV6	YPWPLYGNEGCGWAGWLLSPRGSRP**H**WGP**N**DPRRRSRNLGKVIDTLTCG**L**ADLMGYIPLVGAPLGG**V**A**A**ALAHGVR**AI**ED
	16 170 180 190 200 210 220 230 240
H77 (NC_004102)	GVNYATGNLPGCSFSIFLLALLSCLTVPASAYQVRNSSGLYHVTNDCPNSSIVYEAADAILHTPGCVPCVREGNASRCWV
HCV1	GVNYATGNLPGCSFSIFLLALLSCLTVPASAYQVRNSSGLYHVTNDCPNSSIVYEAADAILHTPGCVPCVREGN**S**SRCWV
HCV2	G**I**NYATGNLPGCSFSIFLLALLSC**I**TVP**V**SA**VE**VRN**I**S**SS**Y**YA**TNDC**S**N**N**SI**TWQLTN**A**V**LH**L**PGCVPC**ENDNGTL**RCW**I**
HCV3	G**I**N**F**ATGNLPGCSFSIFLLAL**F**SCL**IH**PA**ASLEW**RN**T**SGLY**VL**TNDC**S**NSSIVYEA**D**D**V**ILHTPGCVPCV**QD**GN**T**S**T**CW**T**
HCV4	G**I**NYATGNLPGCSFSIFLLALLSCLTVPASA**VHY**RN**A**SG**I**YH**I**TNDCPNSSIVYEA**DHH**ILH**L**PGCVPCVR**T**GN**Q**SRCWV
HCV5	GVNYATGNLPGCSFSIF**I**LALLSCLTVPASA**VPY**RN**A**SG**V**YHVTNDCPNSSIVYEA**DNL**ILH**A**PGCVPCVREGN**V**SRCWV
HCV6	G**I**NYATGNLPGCSFSIFLLALLSCLT**T**PASA**VHYG**NSSG**I**YH**L**TNDCPNSSIVYEA**DAM**ILH**L**PGCVPCVR**V**GN**Q**SRCW**H**
	Core|E1
	241 250 260 270 280 290 300 310 320
H77 (NC_004102)	AVTPTVATRDGKLPTTQLRRHIDLLVGSATLCSALYVGDLCGSVFLVGQLFTFSPRRHWTTQDCNCSIYPGHITGHRMAW
HCV1	A**L**TPTVATRDGKLPTTQLRRHIDLLVGSATLCSALYVGDLCGSVFLVGQLFTFSPRRHWTTQDCNCSIYPGHITGHRMAW
HCV2	**Q**VTP**N**VA**VKHRGAL**T**HN**LR**T**H**V**D**MIVMA**AT**V**CSALYVGD**V**CG**A**V**MI**V**S**Q**A**F**IV**SP**Q**RH**NF**TQ**E**CNCSIY**Q**GHITGHRMAW
HCV3	**P**VTPTVA**VRYVGATT**T**ASI**R**S**H**V**DLLVG**A**AT**M**CSALYVGD**M**CG**A**VFLVGQ**A**FTF**R**PRRH**Q**T**V**Q**T**CNCS**L**YPGH**L**TGHRMAW
HCV4	A**L**TPTVA**APYLGA**P**LES**LRRH**V**DL**M**VG**A**ATLCSALYVGDLCG**GA**FLVGQ**M**FTF**Q**PRRHWTTQDCNCSIY**T**GHITGHRMAW
HCV5	**QI**TPTV**SAPSFGAV**T**AP**LRR**AV**D**Y**L**A**G**G**A**A**LCSALYVGD**A**CG**A**VFLVGQ**M**FT**YR**PR**Q**H**TTV**QDCNCSIY**S**GHITGHRMAW
HCV6	**P**V**S**PT**L**A**VPNAST**P**ATGF**RRH**V**DLLVG**A**A**AV**CSALY**I**GDLCG**G**VFLVGQLFTF**R**PRRHWT**V**QDCNCSIY**T**GH**V**TGHRMAW

Table S6. *Cont.*

	Core∣E1
	321 330 340 350 360 370 380 390 400
H77 (NC_004102)	DMMMNWSPTAALVVAQLLRIPQAIMDMIAGAHWGVLAGIAYFSMVGNWAKVLVVLLLFAGVDAETHVTGGSAGRTTAGLV
HCV1	DMMMNWSPTTALVVAQLLRIPQAILDMIAGAHWGVLAGIAYFSMVGNWAKVLVVLLLFAGVDAETHVTGGSAARTTSGLT
HCV2	DMMLNWSPTLTMILAYAARVPELVLEIIFGGHWGVMFGLAYFSMQGAWAKVIAILLLVAGVDATTYSTGGQAGRTTSGLA
HCV3	DMMMNWSPAVGMVVAHVLRLPQTLFDIIAGAHWGILAGLAYYSMQGNWAKVAIIMVMFSGVDATTYTTGGSAARTARGLT
HCV4	DMMMNWSPTTTLVLAQLMRIPSALVDMLAGGHWGVLAGLAFFSMQANWAKVILVLFLFAGVDAETHVTGGAAGRTASGLT
HCV5	DMMMNWSPTTALLMAQLLRIPQVVIDIIAGGHWGVLFAAAYFASAANWAKVALVLFLFAGVDAETHTVGGSAAQGAGGLA
HCV6	DMMMNWSPTTTYVLSSILRVPQILLEIFLGGHWGVLGAVLYFSMVGNWAKVLAVLLLFAGVDATTTTTGSAVARTTSGLT
	E1∣E2
	401 410 420 430 440 450 460 470 480
H77 (NC_004102)	GLLTPGAKQNIQLINTNGSWHINSTALNCNESLNTGWLAGLFYQHKFNSSGCPERLASCRRLTDFAQGWGPISYANGSGL
HCV1	SLFSPGAKQNIQLINTNGSWHINRTALNCNDSLNTGWLAGLFYYHKFNSSGCPERMASCRPLTDFAQGWGPISYANGSGP
HCV2	GLFSPGPQQNIQLINTNGSWHINRTALNCNDSLQTGFIASLFYTHKFNSSGCPERLSSCRGLDDFRIGWGTLEYETTNGP
HCV3	SLFSPGAQQNLQLVNTNGSWHINSTALNCNDSINTGFIAGLFYYHKFNSTGCPQRLSSCKPITFFKQGWGPLTDANGPSD
HCV4	SLFTPGSRQNLQLINTNGSWHINRTALNCNDSLNTGFLAGLFYAHKFNSSGCPERLASCKRLDDFPQGWGPLGVANGPSD
HCV5	SLFTPGPQQNLQLINTNGSWHINRTALNCNDSLQTGFIAGLLYYHKFNSSGCPERMASCKPLAAFDQGWGTISYANGPSD
HCV6	GLFSPGAKQNLQLINTNGSWHINRTALNCNDSLQTGFIAGLFYYHKFNSSGCPERMSSCKPLTDFDQGWGPITYANGPSS
	481 490 500 510 520 530 540 550 560
H77 (NC_004102)	DERPYCWHYPPRPCGIVPAKSVCGPVYCFTPSPVVVGTTDRSGAPTYSWGANDTDVFVLNNTRPPLGNWFGCTWMNSTGF
HCV1	DQRPYCWHYPPKPCGIVPAKSVCGPVYCFTPSPVVVGTTDRSGAPTYNWGENETDVFVLNNTRPPLGNWFGCTWMNSTGF
HCV2	DEDMRCWHYPPKPCGIVPARTVCGPVYCFTPSPVVVGTTDRQGVPTYTWGENETDVFLLNSTRPPQGAWFGCTWMNGTGF
HCV3	D-RPYCWHYAPRPCDTVPASSVCGPVYCFTPSPVVVGTTDAKGAPTYTWGENETDVFLLESLRPPSGRWFGCTWMNSTGF
HCV4	D-RPYCWHYAPRPCGVVPASTVCGPVYCFTPSPVVVGTTDRLGVPTYTWGENETDVFILNSTRPPGGAWFGCTWMNSTGF
HCV5	D-KPYCWHYPPRPCGVVPARDVCGPVYCFTPSPVVVGTTDRRGCPTYNWGENETDIFLLNNTRPPTGNWFGCTWMNSTGF
HCV6	D-RPYCWHYAPRPCDVVPARTVCGPVYCFTPSPVVVGTTDRRGNPTYTWGENETDVFLLESLRPPTGGWFGCTWMNSTGF
	561 570 580 590 600 610 620 630 640
H77 (NC_004102)	TKVCGAPPCVIGGVGNNTLLCPTDCFRKHPEATYSRCGSGPWITPRCMVDYPYRLWHYPCTINYTIFKVRMYVGGVEHRL
HCV1	TKVCGAPPCVIGGVGNNTLHCPTDCFRKHPEATYSRCGSGPWITPRCLVDYPYRLWHYPCTVNYTIFKVRMYVGGVEHRL
HCV2	TKTCGAPPCRIRRDFTLDLLCPTDCFRKHPDATYIKCGAGPWLTPRCLVDYPYRLWHYPCTVNFTIFKVRMYVGGVEHRL
HCV3	VKTCGAPPCNIYGGPESDLFCPTDCFRKHPEATYSRCGAGPWLTPRCMVDYPYRLWHYPCTVNFTLFKVRMFVGGFEHRF
HCV4	TKTCGAPPCSVQTN-NGSWSCPTDCFRKHPETTYTKCGSGPWLTPRCLVHYPYRLWHYPCTVNYTIFKVRMYVGGIEHRL
HCV5	VKTCGAPPCNLGPT-NNSLKCPTDCFRKHPDATYTRCGSGPWLTPRCLVHYPYRLWHYPCTVNYTIFKVRMFIGGLEHRL
HCV6	VKTCGAPPCQIVPG-NNELLCPTDCFRKHPEATYARCGSGPWLTPRCLVDYPYRLWHYPCTVNFTIHKVRMFVGGVEHRF

Table S6. *Cont.*

	E1/E2
	641 650 660 670 680 690 700 710 720
H77 (NC_004102)	EAACNWTRGERCDLEDRDRSELSPLLLSTTQWQVLPCSFTTLPALSTGLIHLHQNIVDVQYLYGVGSSIASWAIKWEYVV
HCV1	EAACNWTRGERCDLEDRDRSELSPLLLSTTQWQVLPCSFTTLPALSTGLIHLHQNIVDVQYLYGVGSSI**V**SWAIKWEYVV
HCV2	**S**AACN**F**TRG**D**RC**R**LEDRDR**GQQ**SPLL**H**STT**E**W**A**VLPCSF**SD**LPALSTGL**L**HLHQNIVDVQYLYG**LSPA**I**TRYIV**KWE**W**VV
HCV3	**T**AACNWTRGERCD**I**EDRDRSE**QH**PLL**H**STT**ELAI**LPCSFT**PM**PALSTGLIHLHQNIVDVQYLYGVGS**GMVG**WA**L**KWE**F**V**I**
HCV4	EAACNWTRGE**P**C**G**LE**H**RDR**T**ELSPLLLSTTQWQVLPCSFTTLPALSTGLIHLHQNIVDVQYLYGVGS**AVV**SWA**L**KWEYVV
HCV5	EAACNWTRGERCDLEDRDR**A**ELSPLL**HT**TTQW**AI**LPCSFT**PT**PALSTGLIHLHQNIVD**T**QYLYG**LS**SSI**V**SWA**V**KWEY**I**V
HCV6	**D**AACNWTRGERC**E**L**D**DRDR**IEM**SPLL**F**STTQ**LAI**LPCSFTT**M**PALSTGLIHLHQNIVDVQYLYGV**S**SS**VV**SWA**V**KWEYVV
	721 730 740 750 760 770 780 790 800
H77 (NC_004102)	LLFLLLADARVCSCLWMMLLISQAEAALENLVILNAASLAGTHGLVSFLVFFCFAWYLKGRWVPGAVYAFYGMWPLLLLL
HCV1	LLFLLLADARVCSCLWMMLLISQAEAALENLV**V**LNAASLAGTHGLVSFLVFFCFAWYLKGRWVPGA**A**YA**L**YGMWPLLLLL
HCV2	LLFLLLADARVC**A**CLWM**LIILG**QAEAALE**KLIILHS**ASA**A**A**SANG**P**LW**F**FI**FF**IA**AWYLKGR**V**VP**LAT**Y**SVL**G**L**W**SF**LLL**V**
HCV3	L**V**FLLLADARVC**VA**LW**LM**L**M**ISQAEAALENLV**T**LNA**VAA**AGTHG**IGWY**LV**A**FC**A**AW**HVR**G**KL**VP**LVT**Y**SLT**GLW**S**L**A**LL**V**
HCV4	L**A**FLLLADARVC**A**CLWMMLL**V**SQ**V**EAAL**A**NL**ITI**NAAS**A**AGTHG**FWYAIL**F**ICV**AWY**V**KGR**LPAA**A**T**YA**AC**GMWPLLLLL
HCV5	L**A**FLLLADAR**ICT**CLW**I**MLL**VC**QAEAALENV**IV**LNAA**AA**AGTHG**FFWGLLVI**CFAW**HF**KGR**L**VPGA**TYLCL**G**I**WPLLLLL
HCV6	L**A**FL**V**LADAR**ICA**CLW**L**MLL**VG**QAEAALENL**IV**LNAAS**A**AS**TQ**G**WLWG**LLFIC**C**AWY**V**KGR**L**VP**LCT**YA**ALQL**WPLLLL**V**
	E2/p7
	801 810 820 830 840 850 860 870 880
H77 (NC_004102)	LALPQRAYALDTEVAASCGGVVLVGLMALTLSPYYKRYISWCMWWLQYFLTRVEAQLHVWVPPLNVRGGRDAVILLMCVV
HCV1	LALPQRAYALDTEVAASCGGVVLVGLMALTLSPYYKRYISWC**L**WWLQYFLTR**A**EAQLHVWVPPLNVRGGRDAVILLMCVV
HCV2	LALPQ**Q**AYALD**AAEQGEL**GLV**L**LV**IISIF**TL**T**P**A**YK**ILL**S**RSV**WWL**S**Y**M**L**VLA**EAQ**IQQ**WVPPL**EA**RGGRD**GIIWAAVIL**
HCV3	L**L**LPQRAYA**WSGEDS**A**TL**G**AG**VL**ALFGFF**TLSP**W**YK**HWIGRL**MWW**N**QY**TIC**R**C**E**A**AL**Q**VWVPPL**A**VRG**S**RD**G**VILL**TSLL**
HCV4	LALP**E**RAYA**F**D**R**EVA**GSL**GG**G**V**V**VLL**TI**LTLSP**H**YKR**WLARGI**WWLQYF**IA**R**A**EA**L**LHV**Y**VP**S**L**E**VRG**P**RD**S**VI**I**L**TALF**
HCV5	L**L**LP**H**RA**L**ALD**SSDGG**S**V**G**CL**VL**II**L**TIF**TL**T**P**G**YK**KMVVLV**MWWLQYF**IA**RVEA**AI**HVWVPPL**Q**VRGGRDA**II**M**L**T**CLF**
HCV6	LALP**R**RAYA**M**DN**EQ**AAS**L**G**AL**VL**LIITIF**TLSP**A**YK**KLLTSFI**WW**N**QYF**IA**R**A**EA**M**LHVWVP**S**L**R**VRGGRDAVILL**T**C**LL**
	p7/NS2
	881 890 900 910 920 930 940 950 960
H77 (NC_004102)	HPTLVFDITKLLLAIFGPLWILQASLLKVPYFVRVQGLLRICALARKIAGGHYVQMAIIKLGALTGTYVYNHLTPLRDWA
HCV1	HPTLVFDITKLLLA**V**FGPLWILQASLLKVPYFVRVQGLLRICALARK**M**AGGHYVQMAIIKLGALTGTYVYNHLTPLRDWA
HCV2	HP**R**LVF**E**ITK**W**LLAI**L**GP**AYL**L**K**ASLL**R**VPYFVR**AHA**LLR**V**C**TL**V**RHL**AG**AR**Y**I**QM**LLIT**LG**RW**TGTY**I**Y**D**HL**S**PLRDWA
HCV3	**Y**P**S**L**I**FDITKLL**IA**V**L**GPL**YLI**QA**AITTT**PYFVR**AHV**L**VRL**C**M**L**V**R**SVM**GG**K**Y**F**QM**IILSIGRWFN**TY**L**Y**D**HL**A**PLRDWA
HCV4	**H**P**H**L**A**FDITK**Y**LLAI**L**GPL**Y**ILQASLL**R**VPYFVR**AHA**L**IK**IC**S**L**V**R**GVVY**G**K**Y**C**QMA**LL**KLGALTGTY**I**Y**D**HL**A**PLRDWA
HCV5	**H**P**A**L**G**F**E**ITK**I**LL**GI**LGPL**YL**LQ**H**SL**T**K**L**PYF**I**R**ARA**LLR**L**C**L**LA**KHLVY**G**K**YVQ**A**A**LLH**LG**R**LTGTY**I**Y**D**HL**A**PLRDWA
HCV6	**H**P**Q**L**G**F**EV**TK**I**LLA**LL**GPL**YL**LQ**Y**SLLKVPYFVR**AHI**LLR**A**C**M**L**V**R**RL**AGG**K**YVQ**ACLLR**LGA**W**TGTY**I**Y**D**HL**A**PLRDWA

Table S6. *Cont.*

	p7 | NS2
	961 970 980 990 1000 1010 1020 1030 1040
H77 (NC_004102)	HNGLRDLAVAVEPVVFSRMETKLITWGADTAACGDIINGLPVSARRGQEILLGPADGMVSKGWRLLAPITAYAQQTRGLL
HCV1	HNGLRDLAVAVEPVVFSRMETKLITWGADTAACGDIINGLPVSARRGQEILLGPADGMVSKGWRLLAPITAYAQQTRGLL
HCV2	HNGLRDLAVAVEPVVFSRMETKLITWGADTAACGDIINGLPVSARRGQEILLGPADGMVSKGWRLLAPITAYAQQTRGLL
HCV3	HNGLRDLAVAVEPVVFSRMETKLITWGADTAACGDIINGLPVSARRGQEILLGPADGMVSKGWRLLAPITAYAQQTRGLL
HCV4	HNGLRDLAVAVEPVVFSRMETKLITWGADTAACGDIINGLPVSARRGQEILLGPADGMVSKGWRLLAPITAYAQQTRGLL
HCV5	HNGLRDLAVAVEPVVFSRMETKLITWGADTAACGDIINGLPVSARRGQEILLGPADGMVSKGWRLLAPITAYAQQTRGLL
HCV6	HNGLRDLAVAVEPVVFSRMETKLITWGADTAACGDIINGLPVSARRGQEILLGPADGMVSKGWRLLAPITAYAQQTRGLL
	NS2 | NS3
	1041 1050 1060 1070 1080 1090 1100 1110 1120
H77 (NC_004102)	GCIITSLTGRDKNQVEGEVQIVSTATQTFLATCINGVCWTVYHGAGTRTIASPKGPVIQMYTNVDQDLVGWPAPQGSRSL
HCV1	GCIITSLTGRDKNQVEGEVQIVSTA**A**QTFLATCINGVCWTVYHGAGTRTIASPKGPVIQMYTNVDQDLVGWPAPQG**A**RSL
HCV2	GCIITSLTGRDKNQVEGEVQIVSTATQ**S**FLATCINGVCWTVYHGAGTRTIASPKGPVIQMYTN**AEG**DLVGWP**S**P**P**G**TK**SL
HCV3	GCIITSLTGRDKNQVEGEVQIVSTATQTFLATCINGVCWTVYHGAGTRTIASPKGPVIQMYTNVDQDLVGWPAP**P**G**AK**SL
HCV4	GCIITSLTGRDKNQVEGEVQIVSTATQ**S**FLATCINGVCWTVYHGAGTRTIASPKGPVIQMYTNVDQDLVGWPAP**P**G**VK**SL
HCV5	GCIITSLTGRDKNQVEGEVQIVSTATQTFLATCINGVCWTVYHGAGTRTIASPKGPVIQMYTNVD**K**DLVGWP**S**P**P**G**T**RSL
HCV6	GCIITSLTGRDKNQVEGEVQIVSTATQ**S**FLATCINGVCWTVYHGAGTRTIASPKGPVIQMYTNVDQDLVGWPAP**P**G**A**RSL
	1121 1130 1140 1150 1160 1170 1180 1190 1200
H77 (NC_004102)	TPCTCGSSDLYLVTRHADVIPVRRRGDSRGSLLSPRPISYLKGSSGGPLLCPAGHAVGLFRAAVCTRGVAKAVDFIPVEN
HCV1	TPCTCGSSDLYLVTRHADVIPVRRRGDSRGSLLSPRPISYLKGSSGGPLLCPAGHAVG**I**FRAAVCTRGVAKAVDFIPVE**S**
HCV2	**D**PCTCG**AV**DLYLVTR**N**ADVIPVRR**KD**D**R**RG**A**LLSPRP**L**S**T**LKGSSGGP**V**LCP**R**GHAVGLFRAAVC**S**RGVAK**SI**DFIPVE**S**
HCV3	**E**PCTCGS**A**DLYLVTR**D**ADVIP**A**RRRGDS**TA**SLLSPRP**LAC**LKGSSGGP**VM**CP**S**GH**VAGI**FRAAVCTRGVAKA**LQ**FIPVE**T**
HCV4	**A**PCTCG**A**SDL**F**LVTRHADV**V**PVRRRGD**T**RG**A**LLSPRPIS**T**LKGSSGGPLLCP**L**GHAVG**I**FRAAVCTRGVAKAVDF**V**PVE**S**
HCV5	TPCTCGS**A**DLYLVTRHADVIP**A**RRRGD**T**R**A**SLLSPRPISYLKGSSGGP**VM**CP**S**GH**V**VG**V**FRAAVCTRGVAKA**LE**FIPVEN
HCV6	TPCTCGSSDLYLVTR**N**ADVIP**A**RRRGD**N**R**AA**LLSPRPIS**T**LKGSSGGP**I**LCP**S**GH**V**VGLFRAAVCTRGVAK**SL**DF**V**PVEN
	1201 1210 1220 1230 1240 1250 1260 1270 1280
H77 (NC_004102)	LETTMRSPVFTDNSSPPAVPQSFQVAHLHAPTGSGKSTKVPAAYAAQGYKVLVLNPSVAATLGFGAYMSKAHGVDPNIRT
HCV1	LETTMRSPVFTDNSSPPAVPQSFQVAHLHAPTGSGKSTKVPAAYAAQGYKVLVLNPSVAATLGFGAYMSKAHGVDPNIRT
HCV2	L**DIAT**RSP**S**F**S**DNS**T**PPAVPQ**TY**QV**GY**LHAPTGSGKSTKVPAAYA**S**QGYKVLVLNPSVAATLGFGAYMSKAHG**IN**PNIRT
HCV3	L**S**T**QA**RSP**S**F**S**DNS**T**PPAVPQS**Y**QV**GY**LHAPTGSGKSTKVPAAY**V**AQGY**N**VLVLNPSVAATLGFG**SF**MS**R**A**Y**G**I**DPNIRT
HCV4	LETTMRSPVFTDNS**T**PPAVPQ**TY**QVAHLHAPTGSGKSTKVPAAYAAQGYKVLVLNPSVAATLGFGAYMSKAHG**I**DPNIR**S**
HCV5	LETTMRSPVFTDNS**T**PPAVP**HE**FQV**G**HLHAPTGSGKSTKVPAAYAAQGYKVLVLNPSVAATLGFGAYMSRAYGVDPNIRT
HCV6	**M**ETTMRSP**S**FTDNS**T**PPAVPQ**TY**QV**GY**LHAPTGSGKSTKVPAAYA**S**QGYKVLVLNPSVAATLGFG**S**YMS**Q**AHG**I**DPNIRT

Table S6. *Cont.*

	NS2|NS3
	1281 1290 1300 1310 1320 1330 1340 1350 1360
H77 (NC_004102)	GVRTITTGSPITYSTYGKFLADGGCSGGAYDIIICDECHSTDATSILGIGTVLDQAETAGARLVVLATATPPGSVTVSHP
HCV1	GVRTITTGSPITYSTYGKFLADGGCSGGAYDIIICDECHSTDATSILGIGTVLDQAETAGARLVVLATATPPGSVTV**P**HP
HCV2	GVRT**V**TTG**D**PITYSTYGKFLADGGCS**A**GAYDIIICDECHS**V**DAT**T**ILGIGTVLDQAETAG**V**RLVVLATATPPG**T**VT**TP**H**S**
HCV3	G**N**RT**V**TTG**AKL**TYSTYGKFLADGGCSGGAYD**V**IICDECH**AQ**DATSILGIGTVLDQAETAG**V**RL**T**VLATATPPGS**I**TV**P**H**S**
HCV4	GVRTITTG**A**PITYSTYGKFLADGGC**G**GGAYDIIICDECHSTD**S**T**T**ILGIGTVLDQAETAGARLVVLATATPPGSVT**TP**H**A**
HCV5	GVRTVTTGAAITYSTYGKFLADGGCSGGAYD**V**IICDECHS**Q**DAT**T**ILGIGTVLDQAETAGARLVVLATATPPGSVT**TP**HP
HCV6	GVRTITTG**GA**ITYSTYGKFLADGGCSGGAYDIIICDECHSTD**PTTV**LGIGTVLDQAETAG**V**RL**T**VLATATPPGSVTV**P**HP
	1361 1370 1380 1390 1400 1410 1420 1430 1440
H77 (NC_004102)	NIEEVALSTTGEIPFYGKAIPLEVIKGGRHLIFCHSKKKCDELAAKLVALGINAVAYYRGLDVSVIPTSGDVVVVSTDAL
HCV1	NIEEVALSTTGEIPFYGKAIPLEVIKGGRHLIFCHSKKKCDELAAKLVALGINAVAYYRGLDVSVIPTSGDVVVV**A**TDAL
HCV2	NIEEVAL**GHE**GEIPFYGKAIPL**AY**IKGGRHLIFCHSKKKCDELAA**A**L**RGM**G**V**NAVAYYRGLDVSVIPT**Q**GDVVVV**A**TDAL
HCV3	NIEEVAL**GSE**GEIPFYGKAIP**IALL**KGGRHLIFCHSKKKCDELA**S**KL**RGM**G**L**NAVAYYRGLDVSVIPT**T**GDVVV**CA**TDAL
HCV4	NIEEVAL**P**TTGEIPFYG**R**AIPL**AL**IKGGRHLIFCHSKKKCDELA**KQ**L**SS**LG**L**NAVAYYRGLDVSVIPTSGDVVV**CA**TDAL
HCV5	NIEEVAL**PSE**GEIPFYG**R**AIPL**AL**IKGGRHLIFCHSKKKCDELA**KQ**L**TSQ**G**V**NAVAYYRGLDV**A**VIP**AT**GDVVV**C**STDAL
HCV6	NI**TET**AL**P**TTGEIPFYGKAIPLE**Y**IKGGRHLIFCHSKKKCDELA**KQ**L**TS**LG**L**NAVA**F**YRG**V**DVSVIPTSGDVVV**CA**TDAL
	1441 1450 1460 1470 1480 1490 1500 1510 1520
H77 (NC_004102)	MTGFTGDFDSVIDCNTCVTQTVDFSLDPTFTIETTTLPQDAVSRTQRRGRTGRGKPGIYRFVAPGERPSGMFDSSVLCEC
HCV1	MTGFTGDFDSVIDCNTCVTQTVDFSLDPTFTIETTTLPQDAVSRTQRRGRTGRGKPGIYRFVAPGERPSGMFDSSVLCEC
HCV2	MTG**Y**TGDFDSVIDCN**VA**VTQ**I**VDFSLDPTFTI**T**T**Q**T**V**PQDAVSR**S**QRRGRTGRG**RL**GIYR**Y**V**SS**GERPSGMFDS**V**VLCEC
HCV3	MTGFTGDFDSVIDCN**VA**V**E**Q**Y**VDFSLDPTF**S**IET**R**T**A**PQDAVSR**S**QRRGRTGRG**RL**G**T**YR**Y**V**T**PGERPSGMFDS**V**VLCEC
HCV4	MTGFTGDFDSVIDCNT**S**V**I**QTVDFSLDPTF**S**IETTT**V**PQDAVSR**S**QRRGRTGRG**RL**GIYR**Y**V**T**PGERPSGMFD**T**SVLCEC
HCV5	MTGFTGDFDSVIDCN**SA**VTQTVDFSLDPTFTIETTT**V**PQDAVSR**S**QRRGRTGRG**RH**GIYR**Y**V**SS**GERPSG**I**FDS**V**VLCEC
HCV6	MTGFTGDFDSVIDCN**VA**VTQ**V**VDFSLDPTF**S**IETTT**V**PQDAVSR**S**QRRGRTGRGKPG**V**YR**Y**V**SQ**GERPSGMFD**TV**VLCE**A**
	1521 1530 1540 1550 1560 1570 1580 1590 1600
H77 (NC_004102)	YDAGCAWYELTPAETTVRLRAYMNTPGLPVCQDHLEFWEGVFTGLTHIDAHFLSQTKQSGENFPYLVAYQATVCARAQAP
HCV1	YDAGCAWYELTPAETTVRLRAYMNTPGLPVCQDHLEFWEGVFTGLTHIDAHFLSQTKQSGENFPYLVAYQATVCARAQAP
HCV2	YDAG**A**AWYELTPAETTVRLRAY**F**NTPGLPVCQDHLEFWE**A**VFTGLTHIDAHFLSQTKQ**G**GENF**A**YL**T**AYQATVCARA**K**AP
HCV3	YDAGC**S**WY**D**L**Q**PAETTVRLRAY**LS**TPGLPVCQDHL**D**FWE**S**VFTGLTHIDAHFLSQTKQ**Q**G**L**NF**S**YL**T**AYQATVCARAQAP
HCV4	YDAGCAWYELTPAETT**T**RLRAY**F**NTPGLPVCQDHLEFWE**S**VFTGLTHIDAHFLSQTKQ**A**G**D**N**Y**PYLVAYQATVCARA**L**AP
HCV5	YDAGCAWY**D**LTPAETTVRLRAY**L**NTPGLPVCQDHLEFWEGVFTGLT**N**IDAH**M**LSQTKQ**G**GENFPYLVAYQATVC**V**RA**K**AP
HCV6	YD**T**GCAWYELTP**S**ETTVRLRAY**L**NTPGLPVCQDHLEFWEGVFTGLTHIDAHFLSQTKQ**A**GENF**A**YLVAYQATVCARA**K**AP

Table S6. *Cont.*

	NS2 ǀ NS3
	1601 1610 1620 1630 1640 1650 1660 1670 1680
H77 (NC_004102)	PPSWDQMWKCLIRLKPTLHGPTPLLYRLGAVQNEVTLTHPITKYIMTCMSADLEVVTSTWVLVGGVLAALAAYCLSTGCV
HCV1	PPSWDQMWKCLIRLKPTLHGPTPLLYRLGAVQNEVTLTHPITKYIMTCMSADLEVVTSTWVLVGGVLAALAAYCLSTGCV
HCV2	PPSWDVMWKCLTRLKPTLTGPTPLLYRLGAVTNEVTLTHPVTKYIATCMQADLEIMTSTWVLAGGVLAAVAAYCLATGCV
HCV3	PPSWDEMWKCLVRLKPTLHGPTPLLYRLGPVQNEICLTHPITKYIMACMSADLEVTTSTWVLLGGVLAALAAYCLSVGCV
HCV4	PPSWDTMWKCLIRLKPTLHGPTPLLYRLGAVQNEVTLTHPITKYIMACMSADLEVVTSTWVLVGGILAALAAYCLSVGSV
HCV5	PPSWDTMWKCMLRLKPTLTGPTPLLYRLGAVQNEITLTHPITKYIMACMSADLEVITSTWVLVGGVVAALAAYCLTVGSV
HCV6	PPSWDTMWKCLIRLKPTLTGPTPLLYRLGAVQNEIITTHPITKYIMTCMSADLEVITSTWVLVGGVLAALAAYCLSVGCV
	NS3 ǀ NS4A
	1681 1690 1700 1710 1720 1730 1740 1750 1760
H77 (NC_004102)	VGRIVLSGKPAIIPDREVLYQEFDEMEECSQHLPYIEQGMMLAEQFKQKALGLLQTASRQAEVITPAVQTNWQKLEVFWA
HCV1	VGRIVLSGKPAIIPDREVLYREFDEMEECSQHLPYIEQGMMLAEQFKQKALGLLQTASRQAEVIAPAVQTNWQKLEAFWA
HCV2	IGRLHLNDRVVVAPDKEVLYEAFDEMEECASKAALIEQAQRIAEMLKSKIQGLLQQATKQAQDIQPAIQSSWPKLEQFWA
HCV3	VGHIELGGKPALVPDKEVLYQQYDEMEECSQAAPYIEQAQVIAHQFKEKVLGLLQRATQQQAVIEPIVATNWQKLEAFWH
HCV4	VGRVVLSGQPAVIPDREVLYQQFDEMEECSKHLPLVEHGLQLAEQFKQKALGVLSVAGKQAQEAAPVVQSNFAKLEQFWA
HCV5	VGRIILSGRPAIIPDREVLYQQFDEMEECSASLPYMDEARAIAGQFKEKVLGLIGTAGQKAETLKPAATSMWSRAEQFWA
HCV6	CGRITTTGKPAVVPDREVLYQQFDEMEECSRHIPYLAEGQQIAEQFKQKVLGLIQTSAKQAEELKPAVHSAWPKLEQFWQ
	NS4A ǀ NS4B
	1761 1770 1780 1790 1800 1810 1820 1830 1840
H77 (NC_004102)	KHMWNFISGIQYLAGLSTLPGNPAIASLMAFTAAVTSPLTTGQTLLFNILGGWVAAQLAAPGAATAFVGAGLAGAAIGSV
HCV1	KHMWNFISGIQYLAGLSTLPGNPAIASLMAFTAAVTSPLTTSQTLLFNILGGWVAAQLAAPGAATAFVGAGLAGAAIGSV
HCV2	KHMWNFISGIQYLAGLSTLPGNPAVASMMAFSAALTSPLSTSTTILLNIMGGWLASQIAPPAGATGFVVSGLVGAAVGSI
HCV3	KHMWNFVSGIQYLAGLSTLPGNPAVASLMAFTASVTSPLTTNQTMFFNILGGWVATHLAGPQSSSAFVVSGLAGAAIGGI
HCV4	KHMWNFISGIQYLAGLSTLPGNPAIASLMSFTAAVTSPLTTQQTLLFNILGGWVASQIATPTASTAFVVSGLAGAAVGSV
HCV5	KHMWNFVSGIQYLAGLSTLPGNPAVATLMSFTAAVTSPLTTQQTLLFNILGGWVASQIAPPTAATAFVVSGMAGAAVGSI
HCV6	KHMWNFVSGIQYLAGLSTLPGNPAVASLMSFSASLTSPLSTSQTLLLNILGGWVASQLAPPPASTAFVVSGLAGAAVGSI
	1841 1850 1860 1870 1880 1890 1900 1910 1920
H77 (NC_004102)	GLGKVLVDILAGYGAGVAGALVAFKIMSGEVPSTEDLVNLLPAILSPGALVVGVVCAAILRRHVGPGEGAVQWMNRLIAF
HCV1	GLGKVLVDILAGYGAGVAGALVAFKIMSGEVPSTEDLVNLLPAILSPGALVVGVVCAAILRRHVGPGEGAVQWMNRLIAF
HCV2	GLGKILVDVLAGYGAGISGALVAFKIMSGEKPSVEDVVNLLPAILSPGALVVGVICAAILRRHVGQGEGAVQWMNRLIAF
HCV3	GLGRVLLDILAGYGAGVSGALVAFKIMGGELPTAEDMVNLLPAILSPGALVVGVICAAILRRHVGPGEGAVQWMNRLIAF
HCV4	GLGKILVDILAGYGAGVAGAVVTFKIMSGEVPSTEDLVNLLPAILSPGALVVGVVCAAILRRHVGPGEGAVQWMNRLIAF
HCV5	GLGRVLIDILAGYGAGVAGALVAFKIMCGEKPSAEDLVNLLPSILCPGALVVGVICAAVLRRHIGPGEGAVQWMNRLIAF
HCV6	GLGKVLVDILAGYGAGVSGALVAFKIMSGETPSTEDMVNLLPALLSPGALVVGVVCAAILRRHVGPAEGANQWMNRLIAF

Table S6. *Cont.*

	NS4A∣NS4B
	1921 1930 1940 1950 1960 1970 1980 1990 2000
H77 (NC_004102)	ASRGNHVSPTHYVPESDAAARVTAILSSLTVTQLLRRLHQWISSECTTPCSGSWLRDIWDWICEVLSDFKTWLKAKLMPQ
HCV1	ASRGNHVSPTHYVPESDAAARVTAILSSLTVTQLLRRLHQWISSECTTPCSGSWLRDIWDWICEVLSDFKTWLKAKLMPQ
HCV2	ASRGNHV**A**PTHYV**A**ESDA**SQ**RVT**QL**LSSLT**I**T**S**LLRRLH**A**WI**TED**C**PV**PCSGSWLRDIWDW**V**C**SI**L**T**DFK**N**WL**SS**KL**L**P**K**
HCV3	ASRGNHVSPTHYVPESDAAARVTA**L**LSSLTVT**S**LLRRLHQWI**NEDYPS**PCSG**D**WLR**T**IWDW**V**C**T**VLSDFKTWL**S**AK**I**MP**A**
HCV4	ASRGNHVSPTHYVPESDAAARVTAILSSLTVT**S**LLRRLH**K**WI**NEDCS**TPC**DS**SWLR**WE**WDW**V**C**T**VLSDFKTWLKAKL**L**P**R**
HCV5	ASRGNHVSPTHYVPE**T**DA**S**A**K**VT**QL**LSSLTVT**S**LL**K**RLH**T**WI**GEDYS**TPC**D**G**T**WLR**A**IWDW**V**C**TA**L**T**DFK**A**WL**Q**AKL**L**PQ
HCV6	ASRGNHVSPTHYVPETDASRNVTQILSSLT**ITS**LLR**K**LHQWI**NEDWS**TPCS**T**SWLRDIWDW**V**C**T**VLSDFKTWLKAKL**V**P**S**
	NS4B∣NS5A
	2001 2010 2020 2030 2040 2050 2060 2070 2080
H77 (NC_004102)	LPGIPFVSCQRGYRGVWRGDGIMHTRCHCGAEITGHVKNGTMRIVGPRTCRNMWSGTFPINAYTTGPCTPLPAPNYKFAL
HCV1	LPGIPFVSCQRGYRGVWRGDGIMHTRCHCGAEITGHVKNGTMRIVGP**K**TCRNMWSGTFPINAYTTGPCTPLPAPNY**S**FAL
HCV2	**M**PG**L**PF**I**SCQ**K**GYAGVW**A**G**T**GIM**T**TRC**P**CGA**NIS**GHV**RM**GTM**KIT**GP**K**TC**L**N**L**W**Q**GTFPIN**C**YT**E**GPC**V**P**K**P**P**PNYK**T**A**I**
HCV3	LPG**L**PF**I**SCQ**K**GY**K**GVWRGDG**V**M**S**TRC**P**CGA**S**ITGHVKNG**S**MR**LA**GPRTC**A**NMW**H**GTFPIN**E**YTTGP**S**TP**C**P**S**PNY**TR**AL
HCV4	**M**PGIPF**L**SCQ**K**GY**K**G**E**WRGDG**V**MHT**T**C**P**CGAE**LA**GH**I**KNG**S**MRIVGP**K**TC**S**N**T**W**H**GTFPINAYTTGP**GV**P**I**PAPNYKFAL
HCV5	LPG**V**PF**L**SCQRGY**K**GVWRGDG**VNS**T**K**C**P**CGA**T**I**S**GHVKNGTMRIVGP**KL**C**S**N**T**W**H**GTFPINA**T**TTGP**SV**P**A**PAPNYKFAL
HCV6	LPGIPF**L**SCQRGYRGVWRGDG**VC**HT**T**C**T**CGA**VIA**GHVKNGTM**KIT**GPRTC**S**N**T**W**H**GTFPINA**T**TTGP**SV**P**I**PAPNY**TR**AL
	2081 2090 2100 2110 2120 2130 2140 2150 2160
H77 (NC_004102)	WRVSAEEYVEIRRVGDFHYVSGMTTDNLKCPCQIPSPEFFTELDGVRLHRFAPPCKPLLREEVSFRVGLHEYPVGSQLPC
HCV1	WRVSAEEYVEIRRVGDFHYV**T**GMTTDNLKCPCQ**V**PSPEFFTELDGVRLHRFAPPCKPLLREEVSFRVGLH**Q**YPVGSQLPC
HCV2	WRV**A**A**S**EYVE**VTQH**G**S**F**S**YV**T**G**L**T**S**DNLK**V**PCQ**V**P**A**PEFF**SWV**DGV**QI**HRFAP**TPG**P**FF**R**D**EV**T**F**T**VGL**NSFV**VGSQLPC
HCV3	WRV**A**A**NS**YVE**V**RRVGDFHY**IT**G**A**T**E**D**E**LKCPCQ**V**P**AA**EFFTE**V**DGVRLHR**Y**APPCKPLLR**D**E**IT**F**M**VGL**NS**Y**AI**GSQLPC
HCV4	WRVSAEEYVE**V**RRVGDFHYV**T**G**V**T**Q**DN**I**KCPCQ**V**P**A**PEFFTE**V**DGVRLHR**H**APPCKPLLR**D**EV**T**F**S**VGL**NTFV**VGSQLPC
HCV5	WRV**G**A**AD**Y**A**E**V**RRVGD**Y**HY**IT**G**V**T**Q**DNLKCPCQ**V**PSPEFFTELDGVR**I**HRFAPPC**N**PLLREEV**C**F**S**VGLH**S**Y**V**VGSQLPC
HCV6	WRVSAEEYVE**V**RRVGD**S**HYV**V**G**V**T**A**DNLKCPCQ**V**P**A**PEFFTE**V**DGVR**I**HR**Y**APPCKPLLR**D**EV**T**F**S**VGL**SS**Y**A**VGSQLPC
	2161 2170 2180 2190 2200 2210 2220 2230 2240
H77 (NC_004102)	EPEPDVAVLTSMLTDPSHITAEAAGRRLARGSPPSMASSSASQLSAPSLKATCTANHDSPDAELIEANLLWRQEMGGNIT
HCV1	EPEPDVAVLTSMLTDPSHITAEAAGRRLARGSPPS**L**ASSSASQLSAPSLKATCT**T**NHDSPDAELIEANLLWRQEMGGNIT
HCV2	**D**PEPD**TE**VL**A**SMLTDPSHITAEAA**A**RRLARGSPPS**Q**ASSSASQLSAPSLKATCT**THKMAY**D**CDMVD**ANL**F**----MGG**DV**T
HCV3	EPEPDV**S**VLTSML**R**DPSHITAE**T**A**A**RRLARGSPPS**E**ASSSASQLSAPSLKATC**QTHRPH**PDAEL**VD**ANLLW**--**EMG**S**NIT
HCV4	EPEPDVAVLTSMLTDPSHITAEAA**R**RRLARGSPPS**L**ASSSASQLSAPSLKATCTA**H**HDSP**G**A**D**LIEANLLW**----**G**A**N**A**T
HCV5	EPEPDV**T**VLTSML**S**DP**A**HITAE**T**A**K**RRL**D**RGSPPS**L**ASSSASQLSAPSLKATCT**TH**H**--**PDAELIEANLLWR-CMGGNIT
HCV6	EPEPDV**T**V**V**TSMLTDPSHITAE**T**A**A**RRLARGSPPS**L**ASSSASQLSAPSLKATCT**TD**H**--**PDAELIEANLLWR-EMGGNIT

Table S6. *Cont.*

	NS4B/NS5A
	2241 2250 2260 2270 2280 2290 2300 2310 2320
H77 (NC_004102)	RVESENKVVILDSFDPLVAEEDEREVSVPAEILRKSRRFARALPVWARPDYNPPLVETWKKPDYEPPVVHGCPLPPPRSP
HCV1	RVESENKVVILDSFDPLVAEEDEREISVPAEILRKSRRFPPALPIWARPDYNPPLLETWKKPDYEPPVVHGCPLPPPKSP
HCV2	RIESDSKVIVLDSLDSMTEVEDDREPSVPSEYLIPRRKFPPALPPWARPDYNPPVIETWKRPDYEPPTVLGCAL-PTPQA
HCV3	RVESETKVVILDSFEPLRAETDDAELSVAAECFKKPPKYPPALPIWARPDYNPPLLDRWKAPDYVPPTVHGCAL-PRGAP
HCV4	RVESEDKVVVLDSFEPLVAEPDDREVSVAAEILRPSKKFPPALPIWARPDYNPPLVETWKQPDYDPPVVHGCAL-PSKPP
HCV5	RVEAENKVVILDSFEPL-KAEDDREISVSADCFRRGPAFPPALPVWARPGYDPPLLETWKRPDYDPPQVSGCPL-PAGLP
HCV6	RVESENKVVVLDSFDPLTPEYDDEISVSAECHIRPRPKFPPALPIWARPDYNPPLLETWKAPDYEPPVVSGCAL-PPKPT
	2321 2330 2340 2350 2360 2370 2380 2390 2400
H77 (NC_004102)	PVPPPRKKR-VVLTESTLSTALAELATKSFG-SSTSGITGDNTTTSSEPAPSGCPPDSDVESYSSMPPLEGEPGDPDLSD
HCV1	PVPPPRKKR-VVLTESTVSTALAELATKSFG-SSTSGITGDNTTTSSEPASSGCPPDSDAESYSSMPPLEGEPGDPDLSD
HCV2	PVPPPRRRR-KVLTQDNVEGVLREMADKVFS-GDSGHSTGADTGGDTGQDPPDE-AASETGSLSSMPPLEGEPGDPDLEK
HCV3	PVPPPRRKR-IQLDGSNVSAALAALAEKSFPSEENSSSSGVDTQSSTTSKVPPS-GESDSESCSSMPPLEGEPGDPDLCD
HCV4	PVPPPRRKR-VVLSESNVSDALADLAAKSFGQPESDSGAGLTTPTETSDPDPII-DKSDDGSYSSMPPLEGEPGDPDLSD
HCV5	PVPPPRRKRKMELSDSTVSQVLADLAHARFKAPSIEGQDSALGTGSQPDSGPEE-DDSDAASYSSMPPLEGEPGDPDLS-
HCV6	PIPPPRRKRLHLDESTVSHALAQLAEKVFPESDPTPSSDSGLSITSGSPPAPTT-AASDAESYSSMPPLEGEPGDPDLS-
	2401 2410 2420 2430 2440 2450 2460 2470 2480
H77 (NC_004102)	GSWSTVSSGADTEDVVCSMSYSWTGALVTPCAAEEQKLPINALSNSLLRHHNLVYSTTSRSACQRQKKVTFDRLQVLDSH
HCV1	GSWSTVSSAADTEDVVCSMSYTWTGALITPCAAEEQKLPINALSNSLLRHHNLVYSTTSRSACQRQKKVTFDRLQVLDSH
HCV2	SSWSTVSDQE--DSVICSMSYSWTGALITPCGPEEEKLPINPLSNSLMRFHNKVYSTTSRSASLRAKKVTFDRVQVLDAH
HCV3	-SWSTVSSEE--QSVVCSMSYSWTGALITPCSAEEEKLPISPLSNSLLRHHNLVYSTSSRSASQRQKKVTFDRLQVLDDH
HCV4	-SWSTVSSED----VVCSMSYSWTGALVTPCAAEESKLPINPLSNSLLRHHNMVYATTSRSATTRQKKVTFDRMQVVDNH
HCV5	GSWSTVSEDS----VVCSMSYSWTGALITPCSAEEEKLPINPLSNTLLRHHNLVYSTSSRSAGQRQKKVTFDRLQVLDDH
HCV6	GSWSTVSEDD----VVCSMSYSWTGALITPCAAEEEKLPINPLSNSLLRHHNLVYSTTSRSASLRQKKVTFDRVQVLDQH
	NS5A/NS5B
	2481 2490 2500 2510 2520 2530 2540 2550 2560
H77 (NC_004102)	YQDVLKEVKAAASKVKANLLSVEEACSLTPPHSAKSKFGYGAKDVRCHARKAVAHINSVWKDLLEDSVTPIDTTIMAKNE
HCV1	YQDVLKEVKAAASKVKANLLSVEEACSLTPPHSARSKFGYGAKDVRCHARKAVNHINSVWKDLLEDSVTPIDTTIMAKNE
HCV2	YDSVLQDVKRAASKVSARLLSVEEACALTPPHSARSRYGFGAKEVRSLSRRAVNHIRSVWEDLLEDQHTPIDT-IMAKNE
HCV3	YKTALKEVKERASRVKARMLTIEEACALVPPHSARSKFGYSAKDVRSLSSKAINQIRSVWEDLLEDTTTPIPT-IMAKNE
HCV4	YHEVLKEIKARASGVKARLLSVEEACDLTPPHSARSKFGYGAKDVRSHSRKAINHINSVWEDLLEDNTTPIPT-IMAKNE
HCV5	YREVVDEMKRLASKVKARLLPLEEACGLTPPHSARSKYGYGAKEVRSLDKKALKHIEGVWQDLLDDSDTPLPT-IMAKNE
HCV6	YYDVLKEIKLRASTVQAKLLSIEEACDLTPPHSARSKFGYGAKDVRSHASKAINHINSVWEDLLEDNDTPIPT-IMAKNE

Table S6. *Cont.*

	NS5A/NS5B
	2561 2570 2580 2590 2600 2610 2620 2630 2640
H77 (NC_004102)	VFCVQPEKGGRKPARLIVFPDLGVRVCEKMALYDVVSKLPLAVMGSSYGFQYSPGQRVEFLVQAWKSKKTPMGFSYDTRC
HCV1	VFCVQPEKGGRKPARLIVFPDLGVRVCEKMALYDVVSKLP**Q**AVMGSSYGFQYSPGQRVEFLVQAWKSKKTPMGFSYDTRC
HCV2	VFCV**D**P**T**KGG**K**KPARLIV**Y**PDLGVRVCEKMALYD**IAQ**KLP**K**AVMG**P**SYGFQYSP**A**ERV**D**FL**LK**AW**G**SKK**D**PMGFSYDTRC
HCV3	VFCV**D**P**V**KGGRKPARLIV**Y**PDLGVRVCEK**R**ALYDV**IQ**KL**SIET**MG**PA**YGFQYSP**Q**QRVE**R**L**LKM**W**T**SKKTP**L**GFSYDTRC
HCV4	VF**S**V**K**PEKGGRKPARLIV**Y**PDLGVRVCEK**R**ALYDVV**KQ**LP**K**AVMG**AA**YGFQYSP**A**QRVE**Y**L**LK**AW**R**SKK**V**PMGFSYDTRC
HCV5	VF**A**V**E**P**S**KGG**K**KPARLIV**Y**PDLGVRVCEK**R**ALYD**IAQ**KLP**T**A**L**MG**P**SYGFQYSP**A**QRVEFL**LK**AWKSKKTPM**A**FSYDTRC
HCV6	VFCV**D**P**S**KGGRKPARLIV**Y**PDLGVRVCEK**R**ALYD**ITR**KLP**Q**AVMG**DA**YGFQYSP**K**QRVE**Y**L**LKM**W**R**SKKTPMGFSYDTRC
	2641 2650 2660 2670 2680 2690 2700 2710 2720
H77 (NC_004102)	FDSTVTESDIRTEEAIYQCCDLDPQARVAIKSLTERLYVGGPLTNSRGENCGYRRCRASGVLTTSCGNTLTCYIKARAAC
HCV1	FDSTVTESDIRTEEAIYQCCDLDPQARVAIKSLTERLYVGGPLTNSRGENCGYRRCRASGVLTTSCGNTLTCYIKA**Q**AAC
HCV2	FDSTVTE**R**DIRTEE**S**IYQ**A**C**S**L**PEE**AR**T**AI**H**SLTERLYVGGP**M**TNS**K**G**QS**CGYRRCRASGV**F**TTS**M**GNT**M**TCYIKA**L**AAC
HCV3	FDSTVTE**Q**DIR**V**EE**E**IYQCC**N**L**E**P**E**AR**KV**I**S**SLTERLY**C**GGP**MF**NS**K**G**AQ**CGYRRCRASGVL**P**TS**F**GNT**I**TCYIKA**T**AA**A**
HCV4	FDSTVTE**R**DIR**V**EE**EV**YQCCDL**E**P**E**AR**KV**I**SA**LTERLYVGGP**MY**NS**K**G**DL**CGYRRCRASGV**Y**TTS**F**GNTLTCY**L**KA**T**AA**T**
HCV5	FDSTVTE**H**DI**M**TEE**S**IYQ**S**CDL**Q**P**E**AR**A**AI**R**SLT**Q**RLY**C**GGP**MY**NS**K**G**QQ**CGYRRCRASGV**F**TTS**M**GNT**M**TCYIKA**L**A**S**C
HCV6	FDSTVTE**R**DIRTE**ND**IYQ**S**CDLDP**E**AR**K**AI**S**SLTERLYVGGP**MY**NS**K**G**QS**CGYRRCRASGVL**P**TS**M**GNTLTCY**L**KA**Q**AAC
	2721 2730 2740 2750 2760 2770 2780 2790 2800
H77 (NC_004102)	RAAGLQDCTMLVCGDDLVVICESAGVQEDAASLRAFTEAMTRYSAPPGDPPQPEYDLELITSCSSNVSVAHDGAGKRVYY
HCV1	RAAGLQDCTMLVCGDDLVVICESAGVQEDAASLRAFTEAMTRYSAPPGDPPQPEYDLELITSCSSNVSVAHDGAGKRVYY
HCV2	**K**AAG**IV**D**P**TMLVCGDDLVVI**S**ES**Q**G**N**EED**ERN**LRAFTEAMTRYSAPPGD**L**P**R**PEYDLELITSCSSNVSVA**LD**P**R**G**R**R**R**YY
HCV3	RAAGL**RNPDF**LVCGDDLVV**VA**ES**D**GV**D**ED**R**A**A**LRAFTEAMTRYSAPPGD**A**PQP**T**YDLELITSCSSNVSVA**R**D**DK**GKR**Y**YY
HCV4	RAAGL**K**DCTMLVCGDDLVVI**A**ES**D**GV**E**EDA**RA**L**G**AFTEAMTRYSAPPGD**A**PQP**A**YDLELITSCSSNVSVAHD**AT**GKRVYY
HCV5	RAA**K**L**R**DCT**L**LVCGDDLV**A**ICES**Q**G**TH**ED**E**ASLRAFTEAMTRYSAPPGDPP**V**P**A**YDLEL**V**TSCSSNVSVA**R**D**AS**G**N**RVYY
HCV6	RAAGL**K**D**FD**MLVCGDDLVVICESAGVQED**T**A**A**LRAFTEAMTRYSAPPGD**E**PQP**T**YDLELITSCSSNVSVAHDG**T**GKR**Y**YY
	2801 2810 2820 2830 2840 2850 2860 2870 2880
H77 (NC_004102)	LTRDPTTPLARAAWETARHTPVNSWLGNIIMFAPTLWARMILMTHFFSVLIARDQLEQALNCEIYGACYSIEPLDLPPII
HCV1	LTRDPTTPLARAAWETARHTPVNSWLGNIIMFAPTLWARMILMTHFFSVLIARDQLEQAL**D**CEIYGACYSIEPLDLPPII
HCV2	LTRDPTTP**I**ARAAWET**V**RH**S**PVNSWLGNII**QY**APT**I**W**V**RM**VI**MTHFFS**IL**LA**Q**D**T**L**N**Q**N**LN**F**E**M**YGA**V**YS**VN**PLDLP**A**II
HCV3	LTRD**A**TTPLARAAWETARHTPVNSWLGNIIM**Y**APT**I**W**V**RM**VM**MTHFFS**ILQSQEI**L**DRP**L**DFEM**YGA**T**YS**VT**PLDLP**A**II
HCV4	LTRDP**E**TPLARAAWETARHTPVNSWLGNII**LY**APT**I**W**V**RM**V**LMTHFFS**ILQSQEA**LE**K**AL**DFDM**YG**VT**YSI**T**PLDLP**A**II
HCV5	LTRDP**QV**PLARAAWETA**K**H**S**PVNSWLGNIIM**Y**APTLWAR**IV**LMTHFFSVL**QSQE**QLE**K**AL**AF**E**M**YG**SV**YS**VT**PLDLP**A**II
HCV6	LTRDPTTPLARAAWETARHTPVNSWLGNIIM**Y**APT**I**W**V**RM**V**LMTHFFS**ILQ**A**QE**QL**HK**AL**DFD**IYG**VT**YS**VT**PLDLP**A**II

Table S6. *Cont.*

	NS5A∣NS5B
	2881 2890 2900 2910 2920 2930 2940 2950 2960
H77 (NC_004102)	QRLHGLSAFSLHSYSPGEINRVAACLRKLGVPPLRAWRHRARSVRARLLSRGGRAAICGKYLFNWAVRTKLKLTPIAAAG
HCV1	QRLHGLSAFSLHSYSPGEINRVAACLRKLGVPPLRAWRHRARSVRA**K**LLSRGGRAAICGKYLFNWAVRTKLKLTPIAAA**S**
HCV2	**E**RLHGL**D**AFSLH**T**YSP**H**E**LS**RVAA**T**LRKLG**A**PPLRAW**KS**RAR**A**VRA**S**L**IAQ-----------------------------**
HCV3	**E**RLHGLSAF**T**LHSYSP**V**E**L**NRVA**GT**LRKLG**C**PPLRAWRHRAR**A**VRA**K**L**IAQ**GG**K**A**K**ICG**L**YLFNWAVRTK**T**KLTP**LP**AAG
HCV4	QRLHGLSAF**T**LH**G**YSP**H**E**L**NRVA**GS**LRKLGVPPLRAWRHRAR**A**VRA**K**L**IAQ**GGRA**R**ICG**I**YLFNWAV**K**TKLKLTP**LP**AA**A**
HCV5	QRLHGLSAFSLHSYSP**S**EINRVA**S**CLRKLGVPPLRAWRHRAR**A**VRA**K**L-**AQ**GG-AAICG**I**YLFNWAV**K**TK**R**KLTP**L**A**D**A**D**
HCV6	QRLHG**MA**AFSLH**G**YSPGE**L**NRV**G**ACLRKLG**A**PPLRAWRHRAR**A**VRA**K**L**IAQ**GG**K**AAICGKYLFNWAV**K**TKLKLTP**LRG**A**S**
	2961 2970 2980 2990 3000
H77 (NC_004102)	RLDLSGWFTAGYSGGDIYHSVSHARPRWFWFCLLLLAVGV
HCV1	**Q**LDLSGWFTAGYSGGDIYHSVS**R**ARPRWFWFCLLLLAVGV
HCV2	**--**
HCV3	**Q**LDLS**S**WFT**V**G**VG**G**N**DIYHSVS**R**AR**T**R**HLLL**CLLLL**T**VGV
HCV4	**K**LDLS**S**WFT**V**G**AG**GGDIYHSVS**R**ARPR**YLLL**CLLLL**S**VGV
HCV5	RLDLS**S**WFT**V**G**AG**GGDIYHS**M**S**R**ARPR-**LLL**CLLLL**S**VGV
HCV6	**K**LDLSGWF**V**AGYSGGDIYHSVS**R**ARPR**LLLL**CLLLL**T**VGV

Table S7. Proportion of positions under positive selective pressure for all six HCV genotypes, according to the SLAC and FEL method. For all ten viral proteins, the proportion of positively selected positions (dN/dS ratio > 1, *p*-value < 0.05%) is listed, together with the median and IQR (Q1-Q3) over all six HCV genotypes. For each viral protein both the number of positively selected positions according to SLAC and FEL was listed per individual HCV genotype, with the upper percentage for SLAC and the lower for FEL.

%		**HCV1**	**HCV2**	**HCV3**	**HCV4**	**HCV5**	**HCV6**	**Median (IQR = Q1 – Q3)**
Core	SLAC	3.65	0.52	0	0	0	0	0.26 (0–0.52)
	FEL	2.60	0.52	0.52	0	0	1.56	0.52 (0.13–1.3)
E1	SLAC	6.77	0.52	0.52	0.52	0	0	0.52 (0.13–0.52)
	FEL	2.60	1.56	0.52	0	1.04	0.52	0.78 (0.52–1.43)
E2	SLAC	9.37	2.20	1.65	0.38	0	0.83	1.24 (0.83–2.07)
	FEL	6.89	2.20	2.75	1.10	1.93	1.93	2.07 (1.31–2.62)
p7	SLAC	4.76	0	0	0	0	0	0
	FEL	0	0	0	0	0	0	0
NS2	SLAC	3.23	0.46	0	0	0	0	0.23 (0–0.46)
	FEL	1.84	0.92	0	0	0	0	0 (0–0.69)
NS3	SLAC	0.32	0.32	0	0	0	0	0
	FEL	0.16	0.32	0.16	0	0	0	0.08 (0–0.16)
NS4A	SLAC	0	0	0	0	0	0	0
	FEL	0	0	0	0	0	0	0
NS4B	SLAC	0.77	0	0.38	0	0	0	0
	FEL	0.38	0	0.38	0	0	0	0 (0-0.29)
NS5A	SLAC	1.35	0	0.22	0	0	0	0
	FEL	0.67	0	0.22	0	0.22	0.22	0.11 (0–0.22)
NS5B	SLAC	3.37	0.51	0	0	0	0	0
	FEL	3.55	1.18	0.17	0.34	0	0	0.25 (0.04–0.97)
Full-genome	SLAC	3.12	0.53	0.30	0.13	0	0.17	0.23 (0.14–0.47)
	FEL	2.16	0.76	0.53	0.20	0.10	0.40	0.46 (0.13–1.3)

Table S8. Positions under positive selective pressure for all six individual HCV genotypes. For the six HCV genotypes, all positively selected positions (dN/dS ratio > 1, *p*-value < 0.05%), according to SLAC are listed, based on the total alignment and the H77 numbering.

HCV genotype 1	Core: 10, 16, 44, 71, 75, 158, 161<br>E1: 28, 40, 44, 65, 106, 108, 117, 139, 149, 153-154, 163, 192<br>E2: 2-3, 7, 10-12, 15, 18-19, 21-22, 25, 49, 53, 59-62, 78, 80, 91-93, 113-114, 135, 141, 170, 183, 187, 190, 193, 222, 234, 253<br>p7: 29, 38, 62<br>NS2: 22, 58, 108, 121, 140, 145-146<br>NS3: 545, 598<br>NS4A: /<br>NS4B: 36, 145<br>NS5A: 204, 215, 235, 339, 392, 395<br>NS5B: 57, 90, 102, 109, 112, 122, 173, 182, 206, 267, 303, 329, 356, 431, 508, 542-543, 548, 563, 582
HCV genotype 2	Core: 71<br>E2: 1, 12, 17, 22, 25, 61, 78, 119, 165<br>NS2: 125<br>NS3: 288, 360<br>NS5B: 396, 564, 578
HCV genotype 3	E1: 44<br>E2: 81, 113, 158, 179, 197, 343<br>NS4B: 167<br>NS5A: 12
HCV genotype 4	E2: 1, 14, 33, 51
HCV genotype 5	/
HCV genotype 6	Core: 75<br>E2: 4, 17, 60<br>NS2: 118
